# Fluorescence Recognition of Anions Using a Heteroditopic Receptor: Homogenous and Two-Phase Sensing

**DOI:** 10.3390/ijms222413396

**Published:** 2021-12-13

**Authors:** Marta Zaleskaya-Hernik, Łukasz Dobrzycki, Marcin Karbarz, Jan Romański

**Affiliations:** Faculty of Chemistry, University of Warsaw, Pasteura 1, 02-093 Warsaw, Poland; mzaleskaya@chem.uw.edu.pl (M.Z.-H.); dobrzyc@chem.uw.edu.pl (Ł.D.); karbarz@chem.uw.edu.pl (M.K.)

**Keywords:** sulfate extraction, ion pair receptors, fluorescent sensors, crown ethers, squaramides, anthracene

## Abstract

In contrast to monotopic receptor **3**, the anthracene functionalized squaramide dual-host receptor **1** is capable of selectively extracting sulfate salts, as was evidenced unambiguously by DOSY, mass spectrometry, fluorescent and ion chromatography measurements. The receptors were investigated in terms of anion and ion pair binding using the UV–vis and ^1^H NMR titrations method in acetonitrile. The reference anion receptor **3**, lacking a crown ether unit, was found to lose the enhancement in anion binding induced by the presence of cations. Besides the ability to bind anions in an enhanced manner exhibited by ion pair receptors **2** and **4**, changing the 1-aminoanthracene substituent resulted in their exhibiting a lower anion affinity than receptor **1**. By using receptor **1** and adjusting the water content in organic phase it was possible to selectively detect sulfates both by “turn-off” and “turn-on” fluorescence, and to do so homogenously and under interfacial conditions. Such properties of receptor **1** have allowed the development of a new type of sensor capable of recognizing and extracting potassium sulfate from the aqueous medium across a phase boundary, resulting in an appropriate fluorescent response in the organic solution.

## 1. Introduction

Fluorescent sensors provide a visual method for detecting a wide range of chemical species [1,2]. There is a high degree of interest in novel artificial fluorescent receptors capable of detecting ions due to the fact that anions and cations are ubiquitous in nature and play very important roles in many areas, such as biological research, clinical diagnosis, industries and environmental protection [3,4,5,6]. One of the very important classes of anions studied by many research groups is that of tetrahedral oxyanions. A typical example is sulfate anions, which are characterized by large free energy of hydration (ΔG_h_ = −1080 kJ/mol). The high free energy of hydration of superhydrophilic anions means that it is difficult to achieve strong interactions through hydrogen bonding in water. The selective determination of sulfate in an aqueous medium is very important, as this anion plays a crucial role in many biochemical and environmental processes [7,8,9]. Sulfate is the fourth most abundant anion in human plasma, is among the most important macronutrients in cells and is involved in the formation of synovial membranes in joints and mucin proteins [10,11]. Thus, monitoring of human plasma sulfate levels can be used as a marker in patients with rheumatoid arthritis or irritable bowel disease [10,11,12]. 

**Figure 1:**
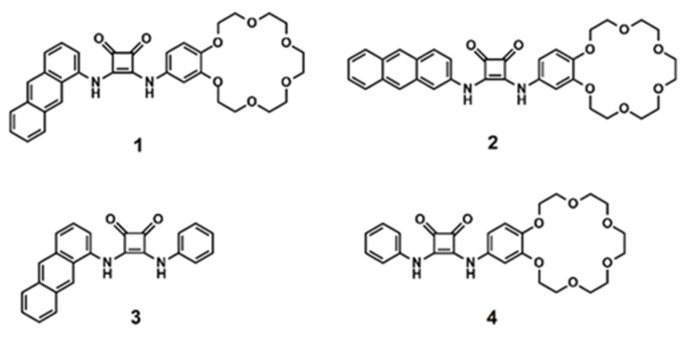
Structures of receptors **1**–**4**.

An abnormal level of sulfate in the urine is a sign of cardiovascular disease or renal failure [13,14]. Sulfate that exists in nature is also a known inorganic pollutant in the environment. For instance, in the oil industry the presence of relatively high sulfate concentrations in the seawater that is injected to increase offshore oil recovery causes problems with sulfate scale deposition. It is difficult to remove and causes severe operational problems with high repair costs. Therefore, it is important to prevent scale build-up by selectively removing sulfate from seawater [15]. In addition, the separation of sulfate anions from aqueous solution is necessary in the nuclear industry, as the anion poses a problem in waste remediation, hindering the vitrification process [16,17,18]. Clearly, solutions for both effective sulfate sensing and extraction are needed. Fluorescence methods are cheap, very selective and highly sensitive and some fluorescent squaramides capable of interacting with anions have been proposed [19,20,21,22,23,24,25,26]. However, only a few fluorescent molecular receptors have been synthesized in the context of recognizing sulfate anions, mostly utilizing anion receptors [27,28,29,30,31,32,33,34]. On the other hand, relatively little progress has been made overall in the field of sulfate salt extraction. This may reflect the intrinsic difficulties associated with the design and synthesis of receptors that extract salts from the aqueous layer to the organic phase, including selective extraction of extremely hydrophilic sulfate salts [35]. It has recently been found that a solution to this problem may lie in the use of ion pair receptors and certain squaramide derivatives have been shown to efficiently extract potassium sulfate from the aqueous to organic phase [36,37,38,39]. To the best of our knowledge, a combination of these two features, leading to molecules capable of selectively extracting sulfates and their fluorescent detection, has not yet been reported. We envisioned that such a solution would provide an opportunity to construct a new type of sensor able to operate not only homogenously but in two phase liquid–liquid conditions. Herein we report the synthesis of two novel fluorescent squaramide-based ion pair receptors **1** and **2** (Figure 1) and study their anion and ion pair binding properties. By adjusting the water content in the system, utilizing the diversity of the stoichiometry of the complexes formed and recognizing the excited-state electron transfer (eT) process, which was recently reported for the interaction of fluorogenic ureas with basic anions [40,41], we propose a solution for affecting the fluorescent response of receptors. The ability to recognize sulfates by increasing or quenching fluorescence intensity using the same molecule is demonstrated. The colour change in fluorescence response was reported as a result of deprotonation of the receptors by basic anions. We also establish the importance of key structural elements of the receptors and the substitution effect, viz. how the fluorophore reporter is connected, on the receptors’ binding properties. To identify the influence of particular functional parts of sensors, anion receptor **3**, lacking a crown ether unit, and receptor **4**, lacking a fluorescent unit, were also tested.

## 2. Results and Discussion

**Scheme 1:**
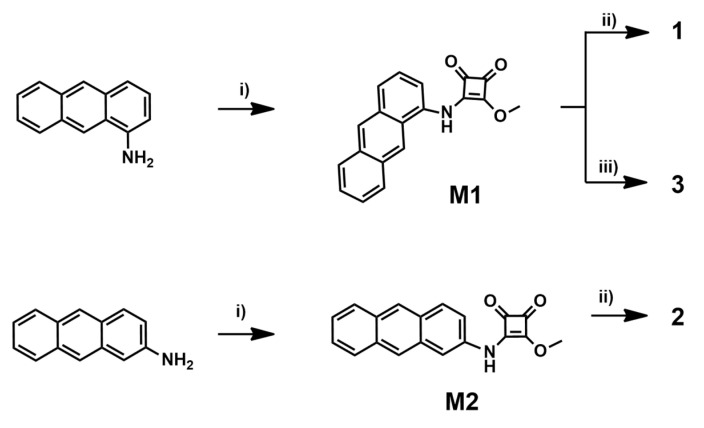
Synthesis of receptors **1**–**3**. Reagents and conditions: (i) 3,4-dimethoxy-3-cyclobutene-1,2-dione, methanol, 48 h, room temperature, yield 63% for M1 and 85% for M2; (ii) 4-aminobenzo-18-crown-6 ether, methanol, DIPEA, 12 h, room temperature, yield 60% for 1 and 70% for 2; (iii) aniline, methanol, DIPEA, 12 h, room temperature, yield 77%.

The synthesis of receptors comprised a two-step protocol relying on successive amidation of dimethyl squarate with 1-aminoanthracene for receptors **1**, **3** and 2-aminoanthracene for receptor **2**, followed by reaction of the obtained monoesters with 4-aminobenzo-18-crown-6 ether to give sensor **1** in 60% yield and receptor **2** in 70% yield. Finally, treatment of monoester **M1** with aniline afforded the anion sensor **3** in 77% yield (Scheme 1).

**Figure 2:**
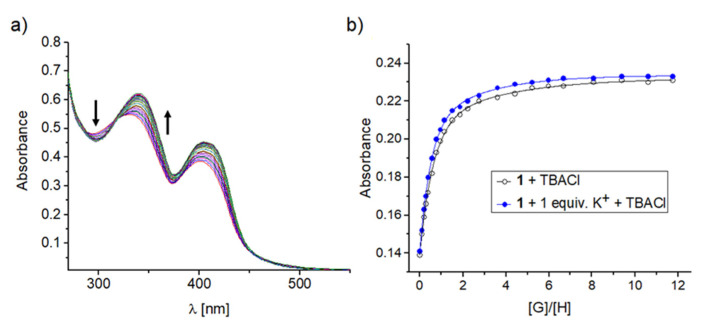
Representative binding behaviour of receptor **1** upon gradual addition of TBACl: (**a**) UV–vis titration spectra and (**b**) corresponding experimental (points) and theoretical (lines) binding isotherms (λ = 430 nm) for TBACl alone and with the presence of 1 equivalent of KPF_6_.

The binding affinities of receptors **1**–**4** for anion and ion pairs were evaluated using the UV–vis titration method in acetonitrile. By dilution experiments, we proved that in the investigated concentration range of 8 × 10^−4^ to 4 × 10^−6^ M the self-association of receptors does not take place. To verify the assumption that receptors **1**, **2** and **4** are capable of binding ion pairs with significantly enhanced affinity relative to the monotopic receptor **3**, selected titrations of receptors **1**–**4** with chloride anions (added as TBA salt) were performed in the presence and absence of cations (added as NaClO_4_ or KPF_6_). We have noticed a bathochromic shift in the UV–vis spectrum upon addition of incremental equivalents of salts to the receptor solutions as a result of complex formation (Figure 2).

Nonlinear regression analysis of the binding isotherms thus obtained allowed the stability constants to be determined, and inspection of these values revealed some interesting trends (Table 1). First, we found that the monotopic receptor **3** is not able to bind anions more strongly in the presence of cations. The enhancement in chloride anion binding by **1** or **2** was found to be greater with the assistance of potassium rather than sodium cations. Remarkably, ion pair receptors **2** and **4** were found to be less effective in chloride anion binding than receptors **1** or **3**.

The latter finding shows that the close proximity of the aromatic protons to the squaramide function in the case of 1-aminoanthracene-based receptors **1** and **3** supports the interaction with anions. This behaviour was maintained when binding studies were extended for other salts for receptors **1** and **2** (Table 2), with the exception of carboxylates, dihydrogenphosphate and fluoride anions, for which a deprotonation event was found. Specifically, both ion pair receptors **1** and **2** could recognize anions more strongly with the assistance of cations, although receptor **2** did so less effectively in the case of both anions and in situ generated salts.

As was recently recognized for the squaramide-based receptors, more complex equilibria were found for the interaction of **1** or **2** with sulfates involved in the formation of a complex with 4:1 stoichiometry (receptor: SO_4_^2−^) [36,37]. The existence of this complex was confirmed by MS experiments carried out in conjunction with the extraction experiments, discussed later. To shed more light on the binding mechanism and explain the lower affinity towards anions in the case of receptor **2** rather than **1**, titration experiments were performed under ^1^H NMR control in MeCN-d_3_. Although we selected the interaction of receptors with bromide salts which should fall in the proper range of estimated Ka’s suitable to be determined by ^1^H NMR technique, we could not fit the obtained titration data and calculate stability constants. This was attributed to the self-association of receptor **1** and **2** in the range of concentration required for ^1^H NMR analyses or other competitive processes [42]. Indeed, in the ^1^H NMR spectrum recorded for 1.4 mM solution of **2** in MeCN-d_3_ the signals corresponding to squaramide protons were shifted remarkably downfield, ca. 11.7 ppm, suggesting the participation of this group in hydrogen bond formation (Supplementary Materials, Figure S47). In the case of receptor **1**, on the other hand, no remarkable changes in the positions of the signals corresponding to the squaramide protons were noted, even with dilution experiments. Thus, we tested receptors **1** and **2** using DOSY experiments that showed a decrease of diffusion coefficients with increasing concentrations of receptors (Supplementary Materials, Table S1). This supports the aforementioned assumptions about the self-association of receptors at higher concentrations.

Nevertheless, analyzing the chemical shifts induced by the addition of bromide ani-ons to the receptors’ solutions shed more light on their binding mode. For titration of **1**, apart from downfield changes in the signals corresponding to the squaramide function, complex formation was reflected by affecting the anthracene proton located at the C-9 position. This signal was remarkably shifted downfield, by Δδ = 0.77 ppm. upon titration of receptor **1** with bromide anions. On the other hand, the signals corresponding to the proton located on the C-2 position of the anthracene unit, as well as both aromatic protons neighboring the squaramide function, were also shifted, albeit less distinctly (Supplementary Materials, Figure S44). Such anion induced downfield shifts of aromatic protons were previously recognized for the interaction of halide anions with diphenyl squaramides and were attributed to the CH···X− interaction rather than a magnetic anisotropy effect [43]. Differentiation of anion-induced changes upon titration of **1** with bromide anions suggests an unequal contribution to anion binding by all plausible CH···X− interactions with the predominance of that at the C-9 position. The change in the linking of the anthracene unit, as in the structure of **2**, or the lack of the anthracene unit in receptor **4**, results in the formation of less stable complexes with anions (Table 1 and Table 2). This shows that the preorganized structure of **1**, which provides additional supports for the interaction with anions, is necessary to effectively recognize salts.

On the other hand, the binding isotherms obtained after titration of **1** with sulfate anions show a multistep binding profile, which confirms more complex equilibria in solution. Although the system lacks strongly coordinating cations, the addition of sulfates to the solution of **1** also induced an upfield chemical shift of the protons assigned to the crown ether unit and this trend was reversed after reaching ca. 0.8 equivalents of anions added (Supplementary Materials, Figure S46). This suggests tight packing of ligands around the sulfate anion required for the initially formed 4:1 complex and its destruction induced by the addition of excess anions. 

**Figure 3:**
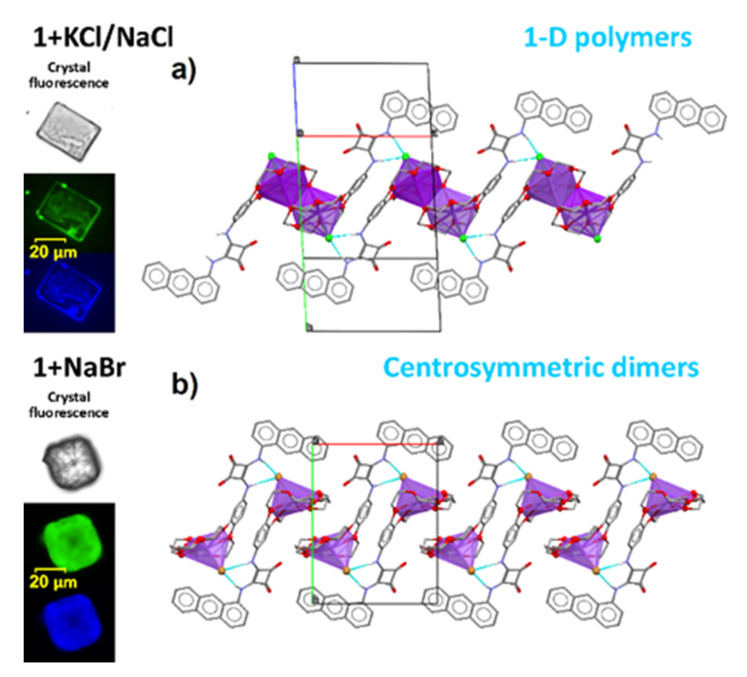
Image presenting fluorescence of the crystals of **1** with KCl/NaCl and **1** with NaBr salts, together with the molecular arrangements of moieties in the solid state: 1-D polymers in **1 +** KCl/NaCl (**a**) and centrosymmetric dimmers in **1** + NaBr (**b**). All C-H hydrogen atoms omitted for clarity.

Our findings were supported by solid state experiments carried out for ion pair complexes with **1**. Although crystallization experiments to obtain complexes with sulfate failed, it proved possible to grow crystals containing chloride and bromide anions. Surprisingly, as receptor **1** has 18-crown-6 moiety targeted to complex K^+^ cations, the obtained crystals, apart from potassium, contain Na^+^ species (**1 +** KCl/NaCl) or are solely composed of NaBr ionic pairs (**1 +** NaBr). In both cases the source of Na^+^ ions is most probably the glassware used in the experiments. The presence of the fluorophore marker, regardless on the salt, causes noticeable fluorescence in UV light, as presented in Figure 3. In both crystal structures, halogen species are bound by the NH groups of the squaramide unit from one side and simultaneously are engaged in coordination of the alkali ion caught in the crown ether ring. However, due to the presence of potassium in **1** + KCl/NaCl which is located in the crown moiety, cations are additionally coordinated by one of the ether O atoms from neighbouring receptor, leading to formation of 1-D polymers (see Figure 3a). This is not the case for the smaller sodium species, where the overly large ether ring is slightly folded around the cation preventing Na^+…^O interactions between adjacent species, and thus only centrosymmetric dimers are formed (see Figure 3b). Interestingly, in both salts there is evident interatomic contact present between the anthracene H atom at position 9 in the ring and the halogen anion; such distance yields 2.74 Å and 2.98 Å for mixed KCl/NaCl and single NaBr salts, respectively.

Next, we shifted our attention and attempted to apply receptors **1**–**3** as salt extractants under liquid–liquid conditions. We found that the presence of the crown ether unit and a proper installation of fluorophore assured not only strong interaction of receptors with ion pairs but provided the required solubility in chloroform. Specifically, only receptor **1** was found to be sufficiently soluble to be applied in extraction experiments. First, we extracted a series of 5 mM aqueous solution of potassium salts of chlorides, bromides, nitrates and sulfates with 5 mM solution of **1** in chloroform, keeping the same volumes of both phases (1 mL). Based on an ion chromatography control, we found that the drop in salt concentration in aqueous phase was 0.46, 0.43, 0.33 and 0.22 mM, respectively. Assuming the formation of 1:1 complexes of **1** with monovalent salts and 4:1 for sulfates, this gives an extraction yield of 9.2, 8.6, 6.6% for aq. KCl, KBr, KNO_3_, and 17.6% for potassium sulfate extraction. This assumption was supported by MS experiments carried out with the organic phase of **1** after extraction of potassium chloride and sulfate, and confirms the presence of characteristics peaks (*m*/*z*) appearing at 633.2 (**1** ⸦ Cl^−^) and 1244.4 (4 × **1** ⸦ SO_4_^2−^), differentiating the complex stoichiometry for both cases (Supplementary Materials, Figure S57). The same conclusion, namely, the formation of the complexes with a higher order of stoichiometry, may be drawn from monitoring the species formed after extraction in the organic phase by DOSY measurements. Specifically, the diffusion coefficient measured for **1** in wet chloroform was found to be D = 0.56 × 10^−9^ m^2^ s^−1^, which is estimated to be too low and suggests some degree of association. After contact with an aqueous solution of KCl the diffusion coefficient dropped to D = 0.53 × 10^−9^ m^2^ s^−1^, and even more in the case of K_2_SO_4_ extraction, to D = 0.48 × 10^−9^ m^2^s^−1^. Competitive extraction experiments carried out with a mixture of potassium salts in aqueous solution (5 mM each) and 20 mM of **1** in chloroform revealed the highest drop in sulfate concentration in the aqueous phase, thus showing the selectivity towards this salt and the ability to overcome the Hofmeister series. After extraction experiments, the concentration of salt was found to be 4.51, 4.50, 4.19 and 4.06 mM for nitrate, chloride, bromide and sulfate salts, respectively. On the other hand, the extraction of sodium salts was found to be both less effective and less selective (Supplementary Materials, Figure S64). With these findings in mind, we anticipated that the formation of 4:1 complexes of receptors with sulfates would open the door to detecting this salt selectively. To verify this hypothesis, we carried out fluorescence experiments in acetonitrile.

**Figure 4:**
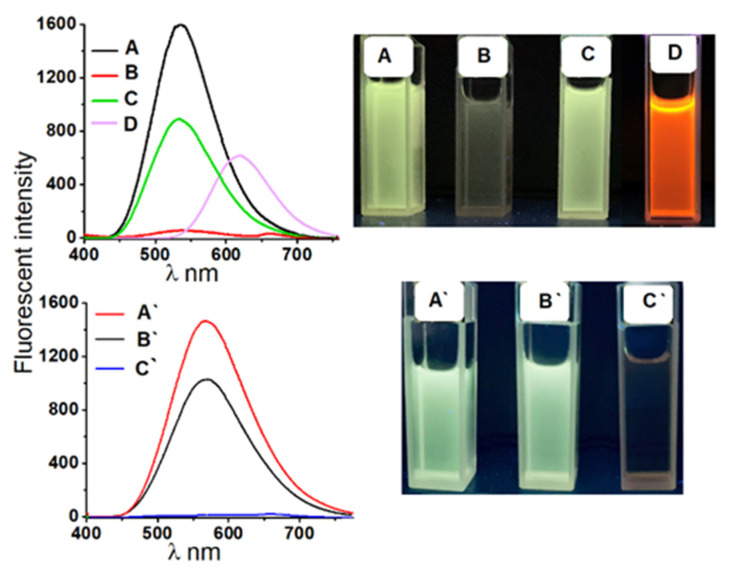
**Top**: Changes observed in the emission spectra of **1** in CH_3_CN (c = 1.05 × 10^−4^ M; excitation at 327 nm). A: receptor **1**; B: upon addition of 100 equivalents of TBA_2_SO_4_; C: upon addition of 100 equivalents of TBACl; D: upon addition of 100 equivalents TBAF, and the corresponding changes seen after UV illumination (λ = 254 nm). **Bottom**: A’: receptor **1** (c = 1.01 × 10^−4^ M) in CH_3_CN; B’: upon addition of TBABr, TBACl, TBANO_3_ (33 equivalents each); C’: upon addition of TBABr, TBACl, TBANO_3_, TBA_2_SO_4_ (25 equivalents each), and the corresponding changes seen after UV illumination (λ = 254 nm).

We found that the addition of anions and in situ generated potassium salts resulted in a decrease in the fluorescence intensity of receptors **1** and **2**. However, upon gradual addition of sulfates to the 1 mM solution of **1** or **2** a decrease in fluorescence intensity was found, and the addition of 100 equivalents of this salt nearly “turned off” the fluorescence of **1** or **2**. The addition of basic anions to the solution of **1** or **2** in acetonitrile produced distinctly emissive deprotonated species (orange fluorescence). (Figure 4 Top, Supplementary Materials, Figures S73 and S80). The effective quenching of locally excited sulfate complexes was attributed to the occurrence of the electron transfer (eT) process recently reported for the interaction of fluorogenic ureas with basic anions [40,41]. Their conversion to excited tautomers was also recognized by the appearance of a poorly detectable emission band at 690 nm, clearly visible in the normalized spectrum (Supplementary Materials, Figure S67). Importantly, the addition of mixtures of various salts (100 equivalents in total) to the receptor solution could detect the presence of sulfates by “turning off” fluorescence. Apart from changes in the emission spectra, this could be observed with the naked eye at higher concentrations, using a UV lamp with λ = 254 nm (Figure 4, Bottom).

**Figure 5:**
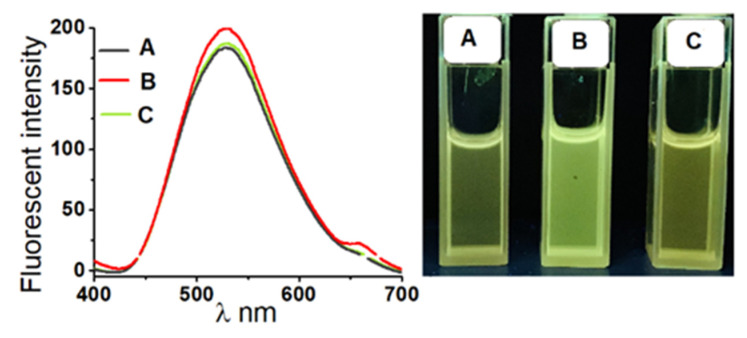
Changes observed in the emission spectrum of **1** in wet CH_3_CN (1% of H_2_O; c = 2.4 × 10^−4^ M, excitation at 327 nm). A: receptor **1**; B: upon addition of 5 equivalents of TBA_2_SO_4_; C: upon addition of 5 equivalents of TBACl; and the corresponding changes seen after UV illumination (λ = 254 nm).

Interestingly, the addition of water to the solution of ion pair receptors **1** and **2** in acetonitrile also decreased their fluorescence intensity, suggesting the interaction of water molecules with the squaramide unit (Supplementary Materials, Figure S76). This was verified by 2D NMR experiments which confirmed exchange coupling of squaramide protons with water molecules in the system (Supplementary Materials, Figure S50a). The addition of sulfate anions to the solution of 2.4 mM of **1** in acetonitrile containing 1% water initially increased the fluorescence intensity, while after 5 equivalents of this salt was exceeded, the trend changed (Supplementary Materials, Figure S76). Similar behaviour was found for the addition of other monovalent salts, albeit requiring the addition of more equivalents of salts to reach the same changes as in the case of sulfate (Figure 5).

**Figure 6:**
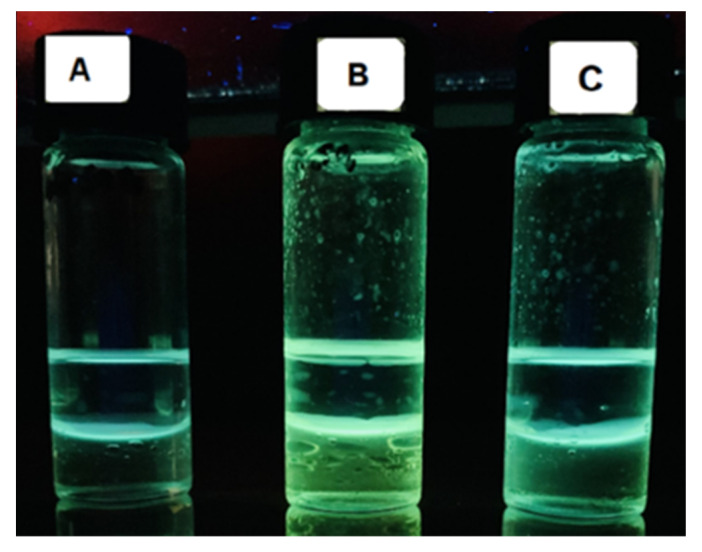
Fluorescence response of solution of receptor **1** (c = 2.3 mM) in CHCl_3_ (under UV light, λ = 254 nm). A: after contact with water; B: after extraction with aqueous K_2_SO_4_ (50 mM); C: after extraction with aqueous KCl (50 mM).

The initial increase in fluorescence in the wet solvent was attributed to the switching of the interaction of receptors with water molecules to the formation of complexes with salts, and this process was found to be more effective for sulfate salts. This was confirmed by 2D NMR measurements, which revealed the cessation of the signal attributed to exchange coupling with water molecules after the addition of salts to the solution of **1** together with a downfield shift of signals attributed to the squaramide protons (Supplementary Materials, Figure S50b). Analogous changes in fluorescence intensity of **1** were found in chloroform. This behaviour allowed for the construction of a unique two-phase fluorescent sensor utilizing the dual functions of the receptors, viz. their capacity for salt extraction and fluorescent visualization. Specifically, selective detection of sulfates in aqueous phase was achieved after contact with the solution of receptor **1** in chloroform, inducing “turn-on” fluorescence (Figure 6).

## 3. Materials and Methods

### 3.1. General Methods

Unless specifically indicated, all other chemicals and reagents used in this study were purchased from commercial sources and were used as received. If necessary, product purification was performed using column chromatography on silica gel (Merck Kieselgel 60, 230–400 mesh) with mixtures of chloroform/methanol. Thin-layer chromatography (TLC) was performed on silica gel plates (Merck Kieselgel 60 F254). The ^1^H and ^13^C NMR spectra used for product characterization were recorded on a Bruker 300 spectrometer (Bruker Corporation, Billerica, MA, USA) using a residual protonated solvent as the internal standard. DOSY, ROESY and HSQC experiments were conducted at 298 K on Varian VNMRS 600 MHz instrument (Varian Inc., Palo Alto, CA, USA) with a residual solvent signal as an internal standard. High resolution mass spectra (HRMS) were measured on a Quattro LC Micromass unit (Waters Corporation, Milford, CT, USA) using the ESI technique. UV–vis analyses were performed using a Thermo Spectronic Unicam UV500 Spectrophotometer (Thermo Fisher Scientific, Waltham, MA, USA). High-performance ion chromatography (HPIC) analyses were performed using a 930 Compact IC Flex apparatus (Metrohm AG, Herisau, Switzerland). All reagents and chemicals were of reagent grade quality and purchased commercially. 

### 3.2. Synthetic Details

**Preparation of 4-nitrobenzo-18-crown-6 ether.** To a 100 mL flask were added (1.0 g, 3.2 mmol) of benzo-18-crown-6 ether, 20 mL of chloroform and the mixture was cooled to 0 °C. Concentrated nitric acid (5 mL) and 2.5 mL of acetic acid was added dropwise while the solution was being stirred and the temperature was being kept low. The mixture was stirred for 24 h at room temperature. Afterwards, the suspension was diluted with chloroform (20 mL) and shaken twice with ice distilled water (40 mL). The organic phases were collected and dried over anhydrous MgSO_4_. Evaporation of the solvent resulted in a gel-like residue which was washed with diethyl ether and kept in a freezer for 1 h. The solid was filtered, washed with diethyl ether and dried in vacuo (1.1 g, 3.1 mmol, yield 96%).

HRMS (ESI) calcd for C_16_H_23_NO_8_Na [M + Na]^+^: 380.1321, found: 380.1330.

^1^H NMR (300 MHz, DMSO-d_6_) δ 7.92–7.85 (d, 1H), 7.75 (s, 1H), 7.22–7.15 (d, 1H), 4.32–4.15 (s, 4H), 3.75–3.65 (s, 4H), 3.62–3.45 (m, 12H).

^13^C NMR (75 MHz, DMSO-d_6_) δ 154.5, 148.3, 141.0, 118.1, 112.1, 107.7, 70.4, 70.3, 70.1, 69.3, 69.1, 68.8, 68.7.

**Preparation of 4-aminobenzo-18-crown-6 ether.** To a 100 mL flask containing degassed solution of (0.6 g, 1.68 mmol) 4-nitrobenzo-18-crown 6-ether in 15 mL of a THF/MeOH (1:4) mixture was added 12 mg of 10% Pd/C. The reaction mixture was kept under a H_2_ atmosphere (balloon pressure) at room temperature for 24 h. The catalyst was removed by filtration through a pad of Celite and washed with methanol. The filtrate was concentrated under reduced pressure to give the crude product in near quantitative yield (0.55 g). The obtained 4-aminobenzo-18-crown-6 ether was used in the next step without further purification.

HRMS (ESI): calcd for C_16_H_25_NO_6_Na [M + Na]^+^: 350.1579, found: 350.1575.

**Preparation of compound M1.** To a 100 mL flask were added (0.80 g, 4.1 mmol) of 1-aminoanthracene, (0.58 g, 4.1 mmol) of 3,4-dimethoxy-3-cyclobutene-1,2-dione and 20 mL 20 mL of methanol. After being stirred for 48 h at room temperature, the reaction mixture was concentrated and purified by silica gel column chromatography (2% methanol in chloroform) to give compound **M1** as a brown solid (0.78 g, 2.6 mmol, 63% yield).

HRMS (ESI): calcd for C_19_H_13_NO_3_Na [M + Na]^+^: 326.0793, found: 326.0782.

^1^H NMR (300 MHz, DMSO-d_6_) δ 11.12 (s, 1H), 8.78 (s, 1H), 8.65 (s, 1H), 8.20–7.90 (m, 3 H), 7.64–7.29 (m, 4H), 4.33 (s, 3H).

^13^C NMR (75 MHz, DMSO-d_6_) δ 185.2, 179.2, 171.4, 167.7, 133.2, 132.0, 131.7, 131.6, 128.9, 128.4, 127.0, 126.8, 126.6, 126.5, 126.4, 125.3, 122.3, 120.4, 60.8.

**Preparation of compound M2.** To a 100 mL flask were added (0.50 g, 2.6 mmol) of 2-aminoanthracene, (0.37 g, 2.6 mmol) dimethyl squarate and 15 mL of methanol. After being stirred for 48 h at room temperature, the reaction mixture was concentrated and purified by silica gel column chromatography (5% methanol in chloroform) to give a yield of compound **M2** as a dark yellow solid (0.66 g, 2.2 mmol, 85% yield).

HRMS (ESI): calcd for C_19_H_13_NO_3_Na [M + Na]^+^: 326.0793, found: 326.0804.

^1^H NMR (300 MHz, DMSO-d_6_) δ 11.02 (s, 1H), 8.54 (s, 1H), 8.46 (s, 1H), 8.16–7.94 (m, 4H), 7.60–7.42 (m, 3H), 4.45 (s, 3H).

^13^C NMR (75 MHz, DMSO-d_6_) δ 188.4, 184.5, 179.6, 169.6, 135.6, 132.3, 131.7, 131.1, 129.9, 129.0, 128.6, 128.2, 126.6, 126.3, 125.7, 125.6, 120.9, 115.0, 61.2.

**Preparation of receptor 1.** To a 100 mL flask were added (0.55 g, 1.68 mmol) of 4-aminobenzo-18-crown 6-ether, (0.51 g, 1.68 mmol) of compound M2 and (1.09 g, 8.4 mmol) of DIPEA in 30 mL of freshly distilled methanol. The reaction mixture was stirred under an argon atmosphere for 12 h, after which the mixture was concentrated and purified by silica gel column chromatography (5% methanol in chloroform) to give receptor **1** as a yellow solid (0.60 g, 1.00 mmol, 60% yield).

HRMS (ESI): calcd for C_34_H_34_N_2_O_8_Na [M + Na]^+^: 621.2213, found: 621.2206.

^1^H NMR (300 MHz, DMSO-d_6_) δ 10.19 (s, 1H), 10.03 (s, 1H), 8.84 (s, 1H), 8.68 (s, 1H), 8.30–8.15 (m, 2H), 8.00–7.88(d, 1H), 7.65–7.35 (m, 4H), 7. 33 (s, 1H), 7.10–6.90 (m, 2H), 4.18–4.00 (m, 4H), 3.85–3.65 (m, 4H), 3.64–3.48 (m, 12H).

^13^C NMR (75 MHz, DMSO-d_6_) δ 183.3, 181.3, 166.7, 166.6, 148.6, 144.6, 133.4, 132.9, 132.2, 131.7, 131.5, 127.1, 126.6, 126.5, 125.6, 125.3, 113.5, 105.0, 70.1, 70.0, 69.1, 68.9, 68.2, 67.9, 65.4.

**Preparation of receptor 2.** To a 100 mL flask were added (0.36 g, 1.1 mmol) of 4-aminobenzo-18-crown 6-ether, (0.33 g, 1.1 mmol) of compound **M2** in 20 mL of freshly distilled methanol. The reaction mixture was stirred under an argon atmosphere for 12 h, after which the precipitate formed was filtered and washed with methanol (5 mL). The yellow solid was recrystallized from methanol (0.46 g, 0.77 mmol, 70% yield).

HRMS (ESI): calcd for C_34_H_34_N_2_O_8_Na [M + Na]+: 621.2213, found: 621.2225.

^1^H NMR (300 MHz, DMSO-d_6_) δ 10.89 (s, 1H), 10.71 (s, 1H), 8.54 (s, 1H), 8.45 (s, 1H), 8.25–7.95 (m, 4H), 7.90–7.79 (m, 1H), 7.50–7.40 (m, 3H), 6.98 (s, 2H), 4.16–4.05 (m, 4H), 3.84–3.68 (m, 4H), 3.69–3.50 (m, 12H).

^13^C NMR (75 MHz, DMSO-d_6_) δ 182.2, 181.1, 166.4, 165.7, 148.7, 144.6, 136.7, 132.9, 132.4, 132.1, 130.9, 130.1, 128.7, 128.6, 126.7, 126.3, 125.2, 120.4, 113.6, 113.0, 104.8, 70.1, 70.1, 69.1, 68.9, 68.3, 68.0.

**Preparation of receptor 3.** To a 50 mL flask were added (0.076 g, 0.82 mmol) of aniline and (0.25 g, 0.82 mmol) of compound **M1** in 15 mL of degassed methanol. The reaction mixture was stirred under an argon atmosphere for 12 h, after which the precipitate formed was filtered and washed with methanol (5 mL). The yellow solid was recrystallized from methanol (0.46 g, 0.77 mmol, 70% yield). The dark green solid was recrystallized by slow diffusion of diethyl ether into methanol solution (0.23 g, 0.63 mmol, 77% yield).

HRMS (ESI): calcd for C_24_H_16_N_2_O_2_ [M + H]^+^: 363.1133, found: 363.1145.

^1^H NMR (300 MHz, DMSO-d_6_) δ 10.27 (s, 1H), 10.13 (s, 1H), 8.85 (s, 1H), 8.68 (s, 1H), 8.24–7.94 (m, 3H), 7.60–7.34 (m, 8H), 7.20–7.05 (m, 1H).

^13^C NMR (75 MHz, DMSO-d_6_) δ 183.2, 182.4, 167.0, 166.8, 139.1, 133.2, 132.2, 131.8, 131.6, 129.9, 128.5, 127.4, 126.7, 126.7, 125.6, 125.5, 123.8, 121.1, 119.1, 118.0.

**Preparation of receptor 4.** To the solution of (0.27 g, 0.83 mmol) of 4-aminobenzo-18-crown-6 ether solution in freshly distilled methanol (10 mL), (0.118 g, 0.83 mmol) of 3,4-dimethoxy-3-cyclobutene-1,2-dione was added. The reaction mixture was stirred under an argon atmosphere for 12 h. The resulting precipitate was isolated by filtration and the obtained solid material was washed several times with methanol. The white solid was dried in vacuo to give monoamide with 86% yield [44]. The resulting monoamide (0.33 g, 0.83 mmol) was suspended in 15 mL of freshly distilled methanol and (0.079 g, 0.85 mmol) of aniline was added under an argon atmosphere. The reaction mixture was stirred under an argon atmosphere for 12 h. The obtained precipitate was filtered and recrystallized from methanol to yield receptor **4** as a yellow solid (0.37 g, 0.60 mmol, 72% yield).

HRMS (ESI): calcd for C_26_H_30_N_2_O_8_Na [M + Na]^+^: 521.1900, found: 521.1888.

^1^H NMR (300 MHz, DMSO-d_6_) δ 11.30 (s, 2H), 7.75–7.55 (d, 2H), 7.50 (s, 1H), 7.45–7.28 (m, 2H), 7.15–6.85 (m, 3H), 4.20–4.00 (m, 4H), 3.90–3.70 (m, 4H), 3.70–3.50 (m, 12H).

^13^C NMR (75 MHz, DMSO-d_6_) δ 181.9, 180.9, 166.0, 165.9, 148.5, 144.3, 139.6, 133.1, 129.7, 123.4, 118.6, 113.1, 110.4, 104.3, 70.0, 69.9, 69.0, 68.8, 67.8, 67.7.

### 3.3. UV–vis Titration Experiments

UV–vis titration experiments were performed in CH_3_CN solution at 298 K. To 10 mm cuvette was added 2.5 mL of freshly prepared (Receptor **1**: c = 2.6 × 10^−5^ M; Receptor **2**: c = 1.7 × 10^−5^ M; Receptor **3**: c = 3.1 × 10^−5^ M; Receptor **4**: c = 2.1 × 10^−5^ M) solution of the studied receptor, and in the case of ion pair binding studies 1 mol equivalent of cation (KPF_6_ or NaClO_4_) was added prior to titrations. Small aliquots of ca. 1.4 × 10^−3^ M TBAX solution containing receptor **1**, receptor **2** or receptor **3**, at the same concentration as in the cuvette, were added and a spectrum was acquired after each addition. The resulting titration data were analyzed using the BindFit (v0.5) package, available online at http://supramolecular.org (accessed on 20 June 2021). Each titration was carried out in duplicate. Reported values are calculated as weighted arithmetic means, where the weights were the errors obtained for each value separately. The given uncertainty of the association constants is the largest of the variance (external or internal).

### 3.4. NMR Titration Experiments

The ^1^H NMR titration was conducted at 298 K in CD_3_CN. In each case, a 500 μL of freshly prepared 1.6 mM solution of Receptor **1** (1.4 mM of Receptor **2**) was added to a 5 mm NMR tube. In the case of ion pair titration, the receptor was firstly pretreated with one equivalent of KPF_6_. Then small aliquots of a solution of TBAX, containing the receptor at constant concentration, were added and a spectrum was acquired after each addition. The resulting titration data were analyzed using the BindFit (v0.5) package, available online at http://supramolecular.org (accessed on 20 June 2021).

### 3.5. Extraction Experiments

A solution of receptor **1** in chloroform (2 mL, 20 mM or 5 mM) was obtained by intensive shaking with an aqueous mixture (no pH adjustment, pH depending on the salts used; above pH 8 there is no phase separation, probably due to receptor deprotonation, eliminating the direct use of basic salts, such as carboxylates, hydrogen phosphates or phosphates) of suitable salts 5 mM each (2 mL) for 30 min. Then, 1 mL of aqueous phase was taken and diluted tenfold. The concentrations of chloride, bromide, nitrite, nitrate, dihydrogenphosphate and sulfate anions in aqueous phase were determined by high performance ion chromatography (HPIC).

### 3.6. Emission Spectra

Solutions of receptor **1** and **2** (c = 1.0 × 10^−4^ M) in CH_3_CN or CHCl_3_ were titrated with small aliquots of TBAX solution containing **1** or **2** at the same concentration as in the cuvette. Successive scans were performed measuring fluorescence (λ _ex_ = 327 nm for **1** and 345 nm for **2**) emission between 300 and 700 nm.

## 4. Conclusions

In conclusion, a family of squaramide-based receptors was synthesized. An anthracene unit located in close proximity to the anion binding domain of receptors provided the ability to detect salts optically. Ion pair sensor **1** was found to interact with salts most effectively as a result of its ditopic feature and properly oriented anthracene moiety. It is capable of selectively extracting extremely hydrophilic sulfate salts from the aqueous to organic phase. By adjusting the content of water in receptor solutions, different fluorogenic responses towards sulfates were achieved. The addition of sulfate salts to the solution of **1** in dry acetonitrile induced fluorescence quenching, while in wet solvents the formation of sulfate complexes with **1** promoted an increase in fluorescent intensity. These two features, i.e., the ability to extract and detect ions, have been combined and used to build a new type of optical sensor capable of working under interfacial conditions. Thus, homogenous and two-phase sensing systems were developed. The interaction of receptors with basic anions, which promoted deprotonation of the receptors, allowed for the construction of another system which involved changing the fluorescence colour.

## Figures and Tables

**Table 1:** Apparent association constants (K_a_) for interactions between receptors **1**–**4** and chloride anions in the absence or presence of 1 equivalent of sodium perchlorate or potassium hexafluorophosphate in acetonitrile *^(a)^*.

L	1	2	3	4
K_TBACl_	2.07 × 10^5^	1.14 × 10^5^	2.06 × 10^5^	9.24 × 10^4^
K_NaCl_	2.45 × 10^5^	1.76 × 10^5^	1.60 × 10^5^	1.13 × 10^5^
K_KCl_	3.10 × 10^5^	2.23 × 10^5^	1.59 × 10^5^	1.90 × 10^5^

*^(a)^* UV–vis, solvent CH_3_CN, temperature 298 K; [**1**] = 2.6 × 10^−5^ M; [**2**] =1.7 × 10^−5^ M; [**3**] =3.1 × 10^−5^ M; [**4**] = 2.1 × 10^−5^ M; TBACl ∼ 1.4 × 10^−3^ M; 1:1 binding; the resulting titration data were analyzed using the BindFit (v0.5) package, available online at http://supramolecular.org; errors < 10%.

**Table 2:** Apparent association constants (K_a_) for interactions between receptors **1** or **2** and anions in the absence or presence of 1 equivalent of potassium hexafluorophosphate *^(a)^*.

	1	1 +1 Equivalent of K^+^	2	2 +1 Equivalent of K^+^
Br^−^	2.73 × 10^4^	4.50 × 10^4^	2.16 × 10^4^	3.15 × 10^4^
NO_2_^−^	2.89 × 10^4^	6.50 × 10^4^	2.11 × 10^4^	5.66 × 10^4^
NO_3_^−^	1.85 × 10^4^	2.56 × 10^4^	1.02 × 10^4^	1.72 × 10^4^
SO_4_^2−^	*^(b)^*	*^(b)^*	*^(b)^*	*^(b)^*

*^(a)^* UV–vis, solvent CH_3_CN, temperature 298 K; [**1**] = 2.6 × 10^−5^ M; [**2**] = 1.7 × 10^−5^ M; TBAX ∼ 1.5 × 10^−3^ M; 1:1 binding; the resulting titration data were analyzed using the BindFit (v0.5) package, available online at http://supramolecular.org; errors < 10%. *^(b)^* The data obtained could not be fitted to an appropriate binding model.

## Data Availability

All data generated or analysed during this study are included in this published article and its Supplementary Materials file.

## Supplementary Materials

The following are available online at https://www.mdpi.com/article/10.3390/ijms222413396/s1.
