# Supplementary material for: Fluorescence Recognition of Anions Using a Heteroditopic Receptor: Homogenous and Two-Phase Sensing
Source: Int J Mol Sci. 2021 Dec 13;22(24):13396. doi: 10.3390/ijms222413396 (PMC8703701; doi:10.3390/ijms222413396)
Supplement: Supplementary file 1 [file ijms-22-13396-s001.zip › ijms-1502225-supplementary.pdf]

Supporting Information

Fluorescence recognition of anions using a heteroditopic receptor: homogenous and two-phase sensing

Marta Zaleskaya-Hernik[1], Łukasz Dobrzycki[1], Marcin Karbarz[1] and Jan Romański[1]

[1] Faculty of Chemistry, University of Warsaw, Pasteura 1, PL 02-093 Warsaw, Poland
* Correspondence: E-mail: jarom@chem.uw.edu.pl

Table of Contents

1. **General information** S1
2. **NMR spectra** S2
3. **Dilution studies** S8
4. **UV-vis titration experiments** S11
5. **NMR Titration experiments** S23
6. **^{1}H DOSY and 2D experiments** S27
7. **Extraction experiments – mass spectrometry and ^{1}H NMR** S30
8. **Extraction experiments - ion chromatography** S31
9. **Emission spectra** S35
10. **Crystal data** S46
11. **References** S52

General information

Unless specifically indicated, all other chemicals and reagents used in this study were purchased from commercial sources and used as received. If necessary purification of products was performed using column chromatography on silica gel (Merck Kieselgel 60, 230-400 mesh) with mixtures of chloroform/methanol. Thin-layer chromatography (TLC) was performed on silica gel plates (Merck Kieselgel 60 F254).

^{1}H and ^{13}C NMR spectra used in the characterization of products were recorded on Bruker 300 spectrometer using a residual protonated solvent as internal standard. DOSY experiments were conducted at 298 K on Varian VNMRS 600 MHz instruments with a residual solvent signal as an internal standard.

Mass spectra were measured on Quattro LC Micromass or Shimadzu LCMS-IT-TOF unit.

UV-vis analyses were performed using Thermo Spectronic Unicam UV500 Spectrophotometer.

Fluorescence emission spectra were measured on a Hitachi F-7100 Fluorescence Spectrophotometer at 298K.

High-performance ion chromatography (HPIC) analyses were performed using a 930 Compact apparatus.

NMR spectra

^{1}H and ^{13}C NMR spectra were recorded on a Bruker 300 MHz spectrometer. ^{1}H NMR chemical shifts δ are reported in ppm referenced to residual solvent signal (DMSO-d_6).

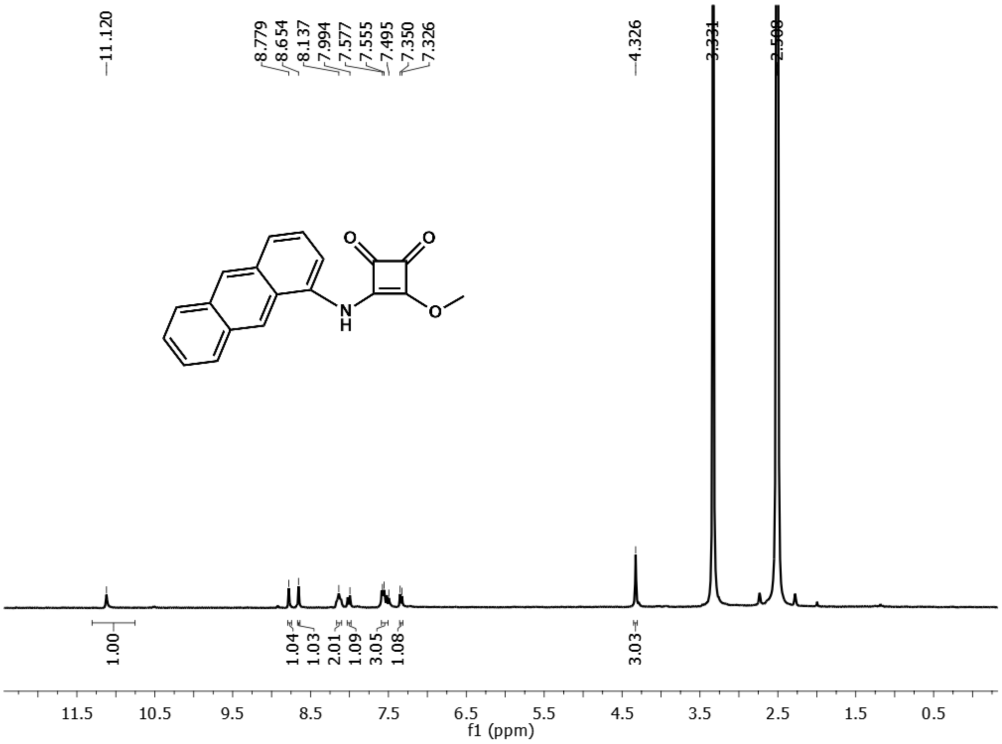

Fig. S1. ^{1}H NMR spectrum of compound **M1** in DMSO-d_6.

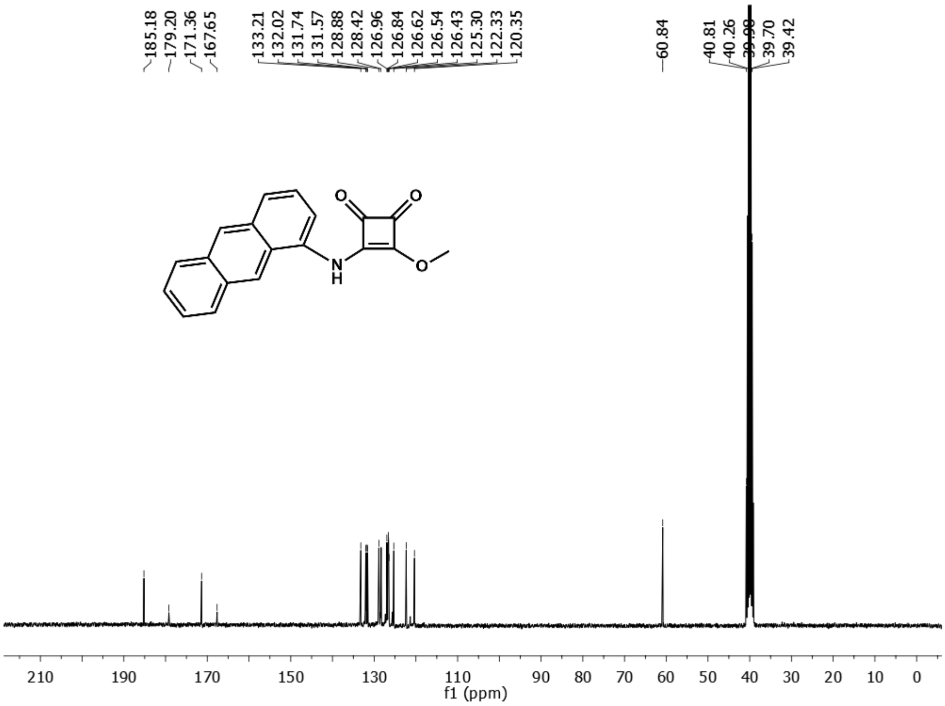

Fig. S2. ^{13}C NMR spectrum of compound **M1** in DMSO-d_6.

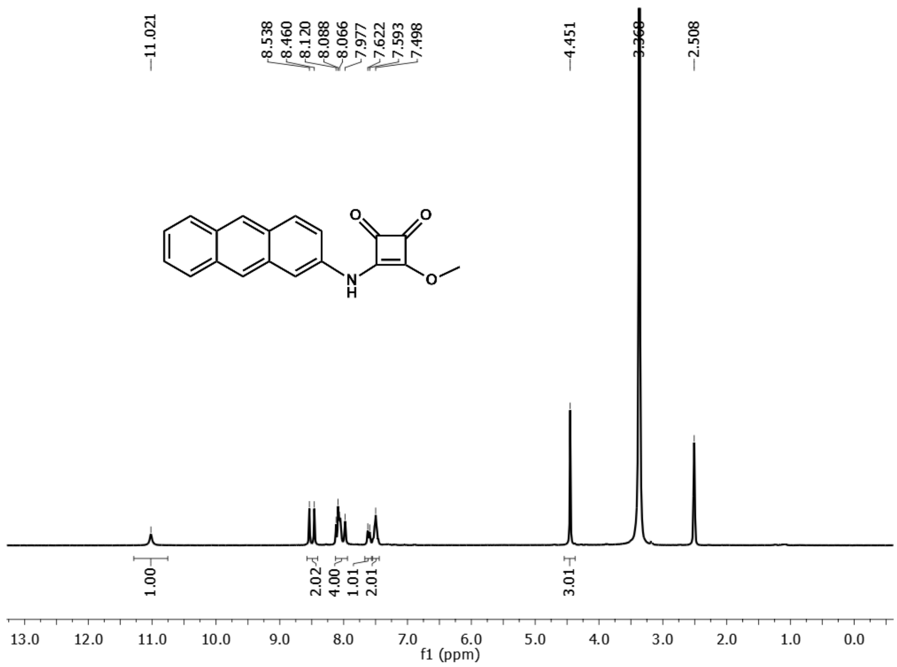

Fig. S3. ^{1}H NMR spectrum of compound **M2** in DMSO-d_6.

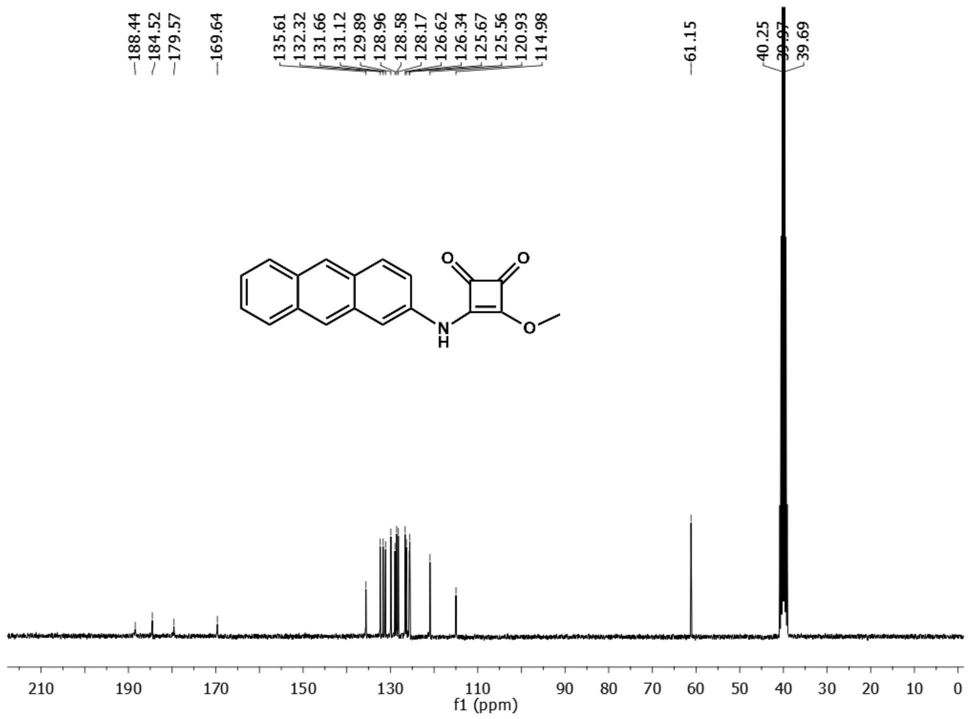

Fig. S4. ^{13}C NMR spectrum of compound **M2** in DMSO-d_6.

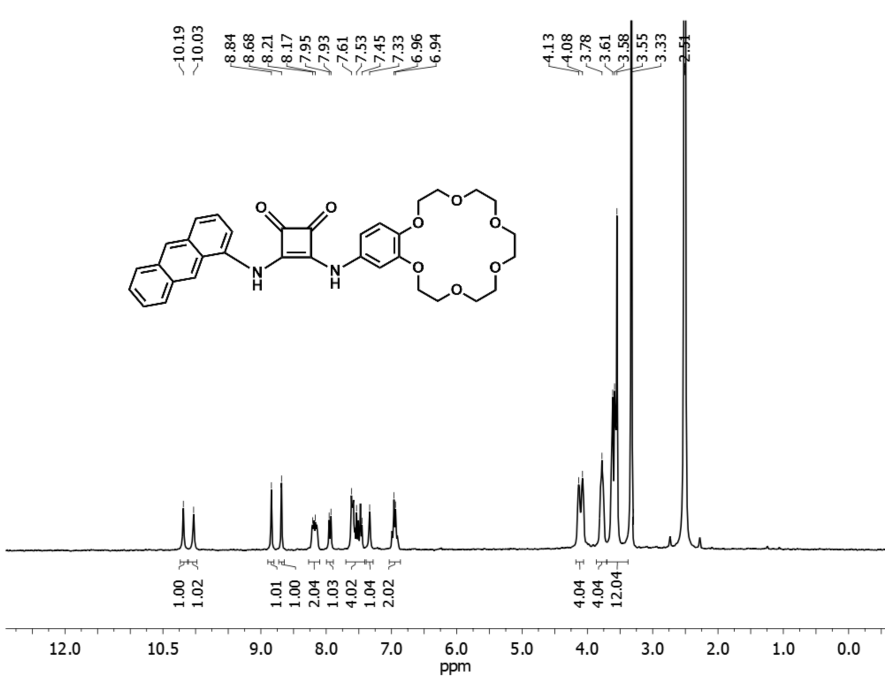

Fig. S5. ^{1}H NMR spectrum of Receptor **1** in DMSO-d_6.

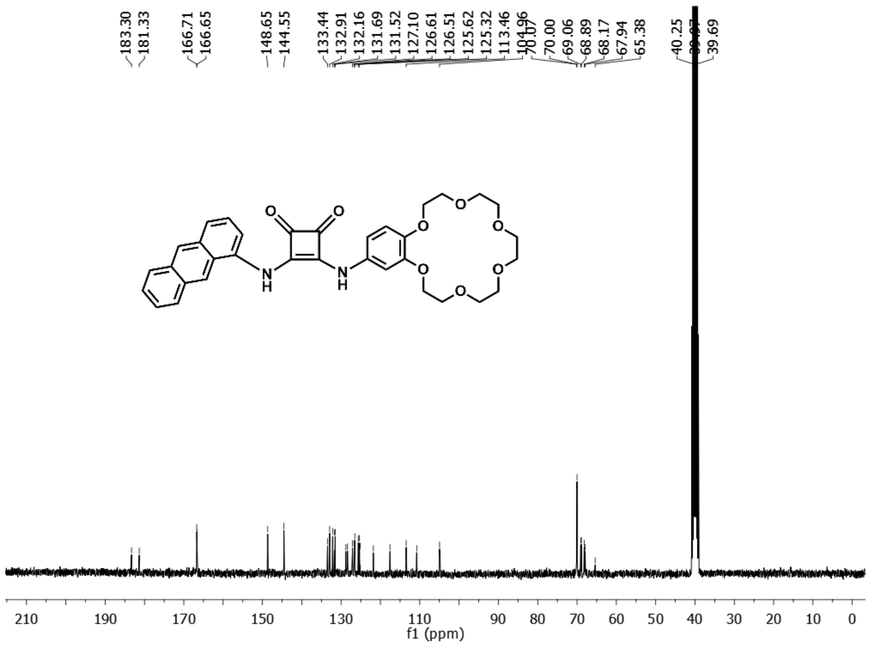

Fig. S6. ^{13}C NMR spectrum of Receptor **1** in DMSO-d_6.

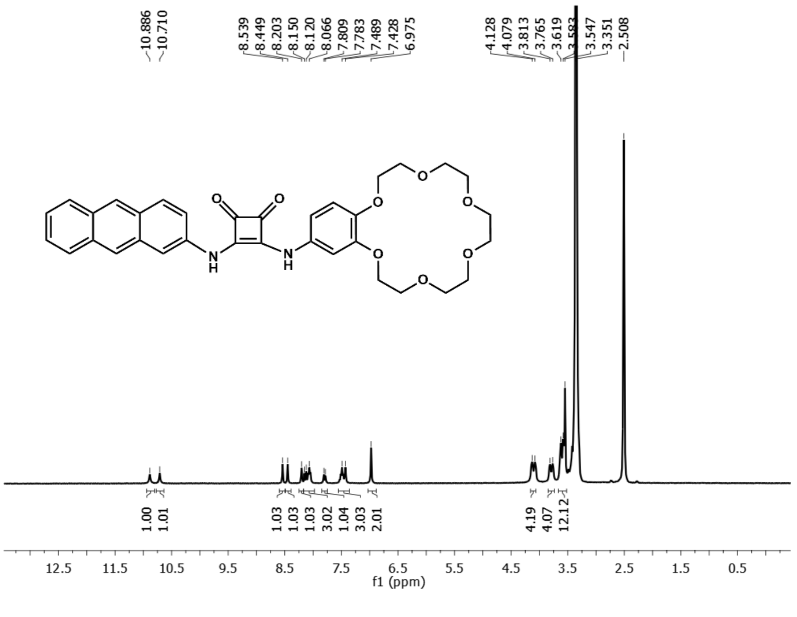

Fig. S7. ^{1}H NMR spectrum of Receptor **2** in DMSO-d_6.

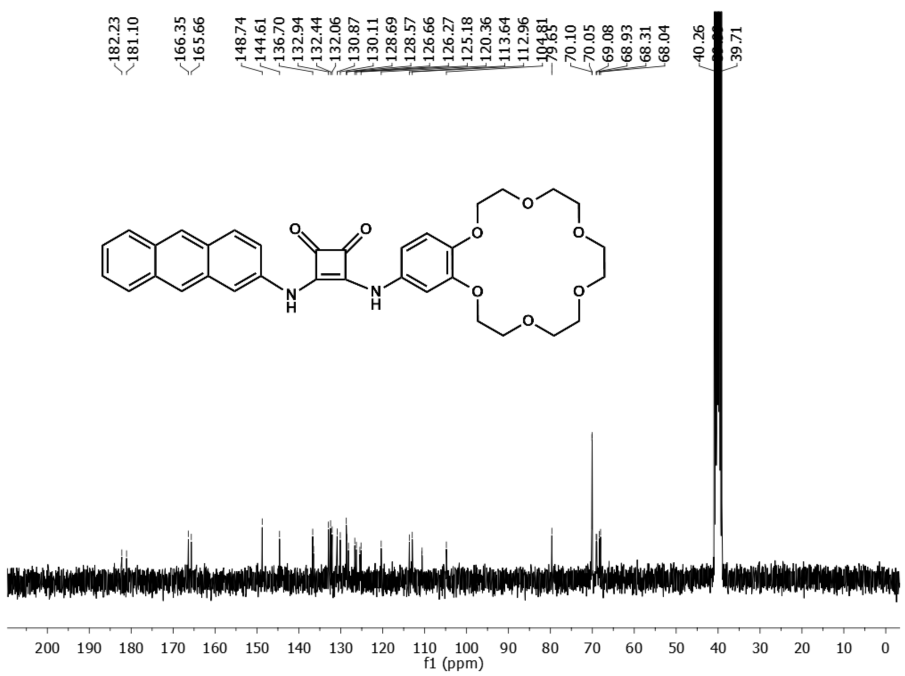

Fig. S8. ^{13}C NMR spectrum of Receptor **2** in DMSO-d$_6$.

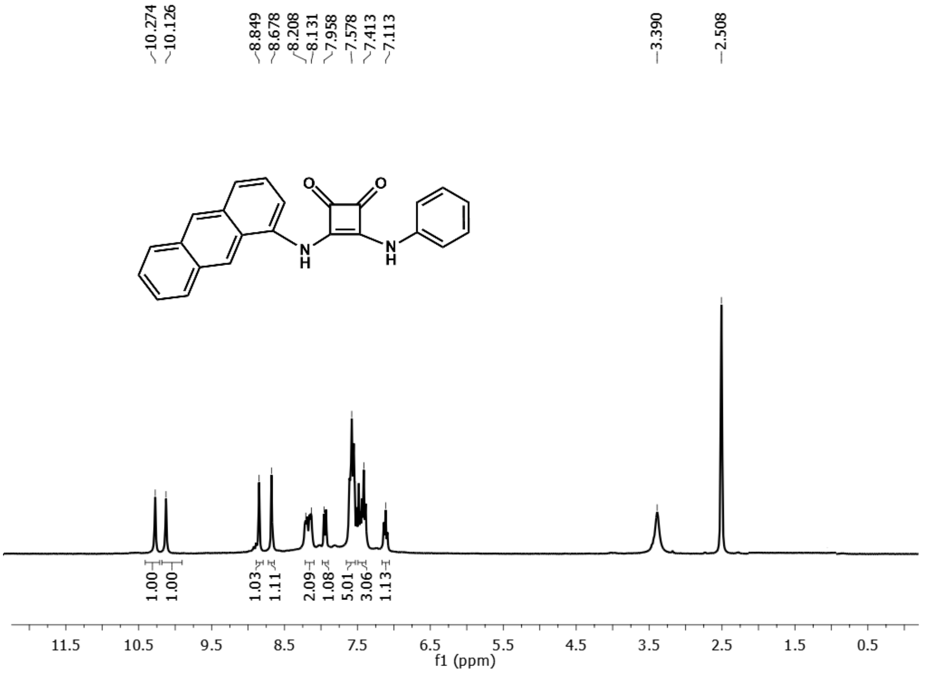

Fig. S9. ^{1}H NMR spectrum of Receptor **3** in DMSO-d$_6$.

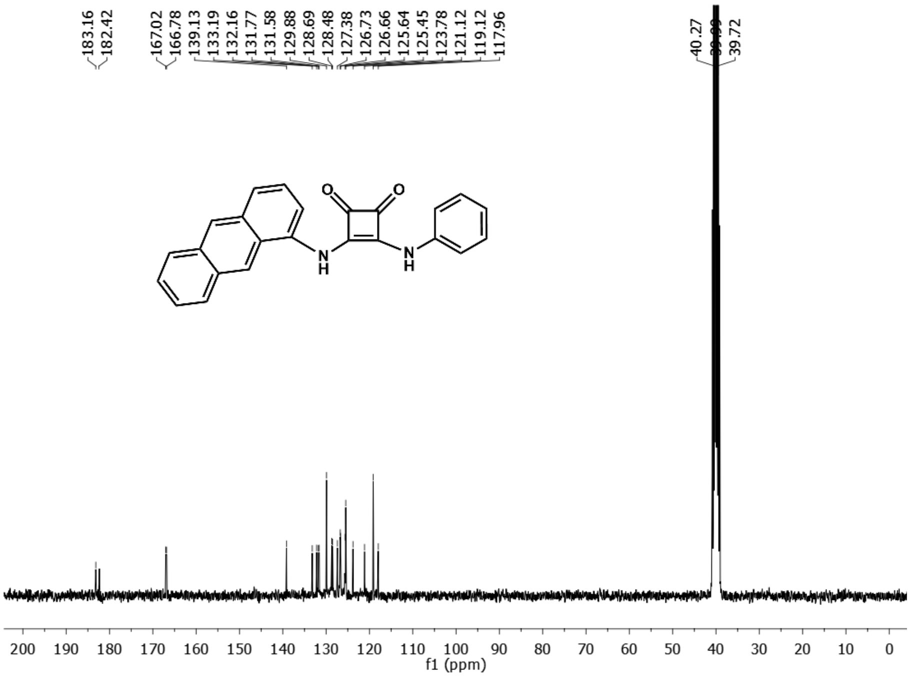

Fig. S10. ^{13}C NMR spectrum of Receptor **3** in DMSO-d_6.

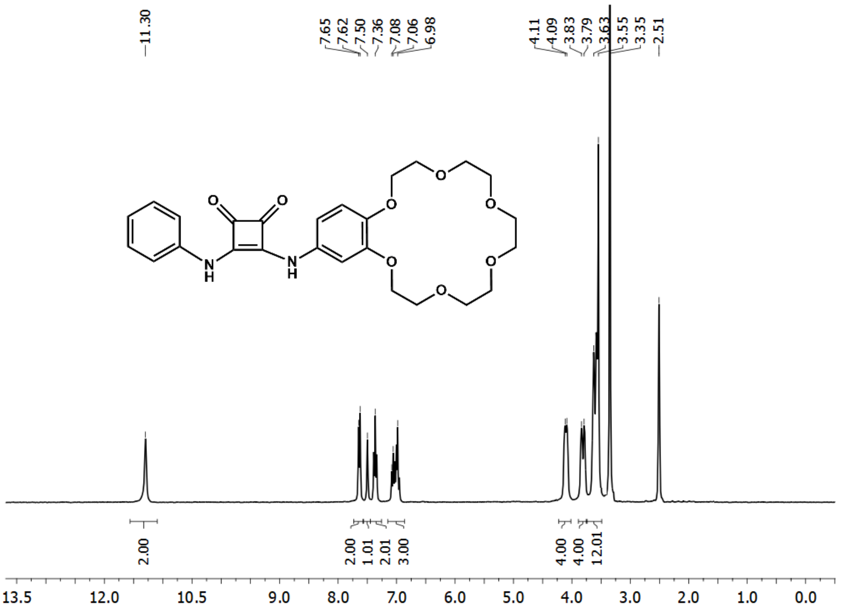

Fig. S11. ^{1}H NMR spectrum of Receptor **4** in DMSO-d_6.

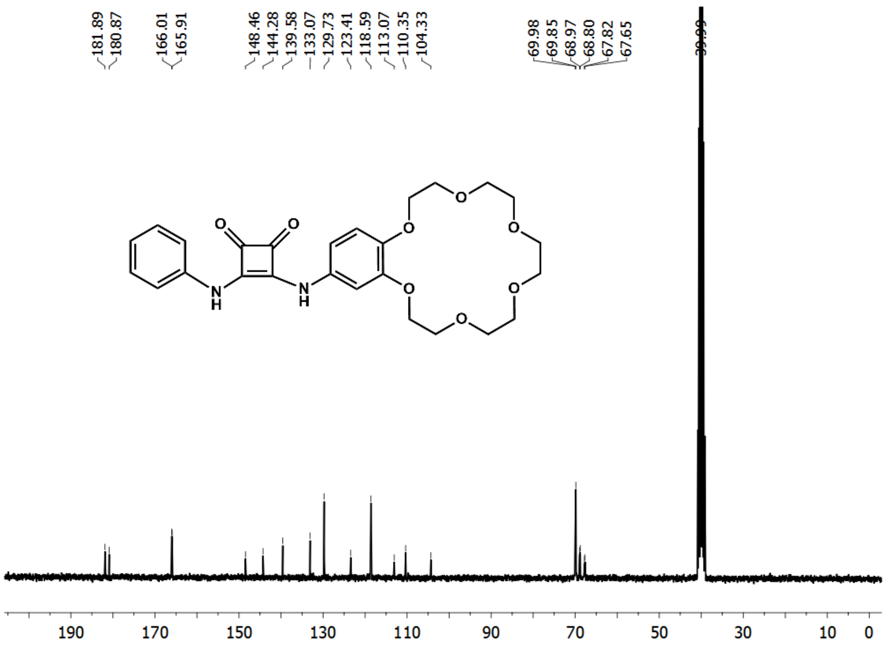

Fig. S12. ^{13}C NMR spectrum of Receptor **4** in DMSO-d_6.

Dilution studies

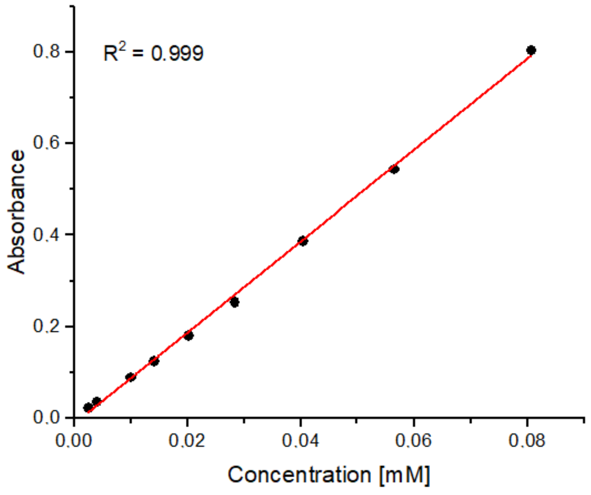

Fig. S13. Dilution curve of Receptor **1** in CH_3CN.

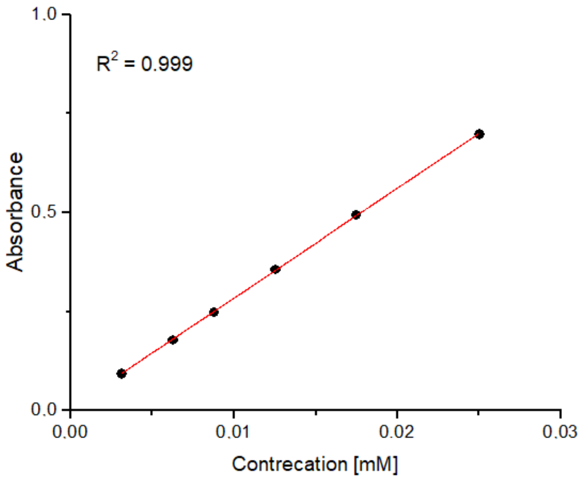

Fig. S14. Dilution curve of Receptor **2** in CH_3CN.

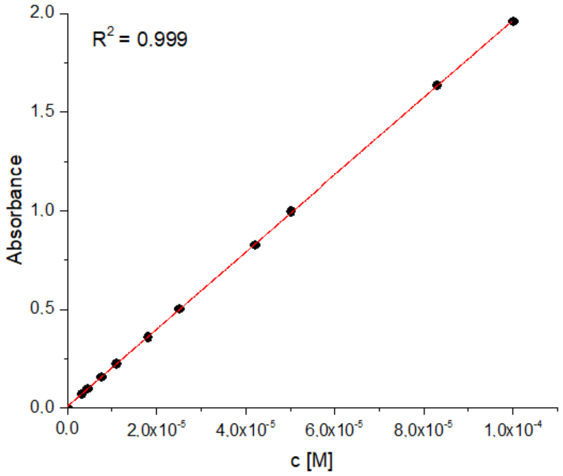

Fig. S15. Dilution curve of Receptor **4** in CH_3CN.

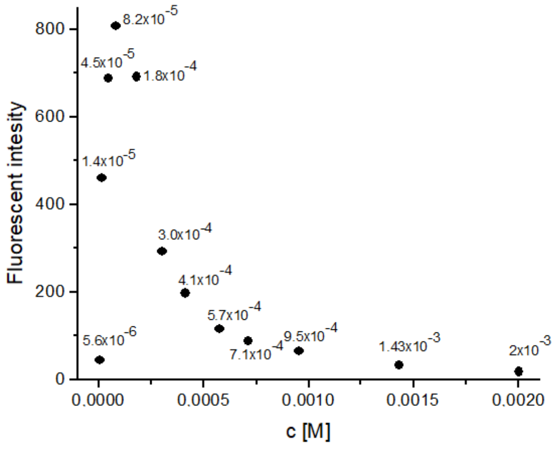

Fig. S16. Dilution curve of Receptor **1** in CH_3CN (excitation at 327 nm).

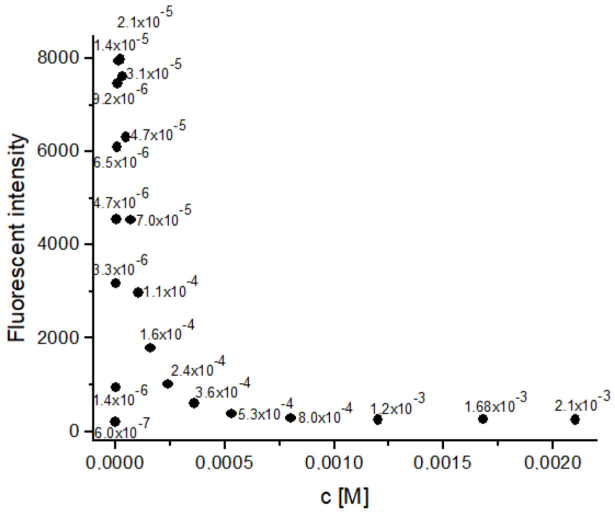

Fig. S17. Dilution curve of Receptor **2** in CH_3CN (excitation at 345 nm).

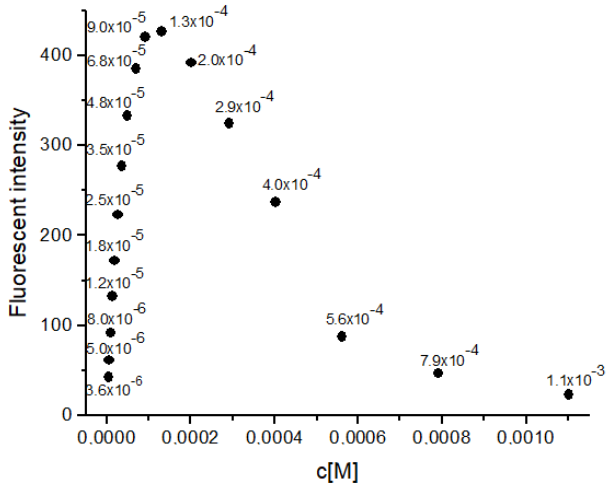

Fig. S18. Dilution curve of Receptor **3** in CH_3CN (excitation at 390 nm).

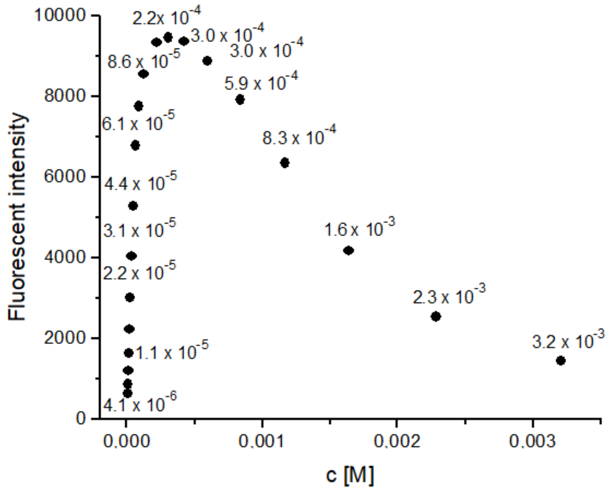

Fig. S19. Dilution curve of 1-aminoanthracene in CH_3CN (excitation at 396 nm).

UV-vis titration experiments

UV-vis titration experiments were performed on a Thermo Spectronic Unicam UV 500 spectrophotometer in CH_3CN solution at 298K. To 10 mm cuvette was added 2.5 mL of freshly prepared (Receptor **1** – c = 2.6 ×10^{-5} M , Receptor **2** – c = 1.7 ×10^{-5} M, Receptor **3** - c = 3.1×10^{-5} M; Receptor **4** - c = 2.1×10^{-5} M) solution of studied receptor and in case of ion pair binding studies 1 mol equivalent of cation (KPF_6 or $NaClO_4$) was added prior titrations. Small aliquots of ca. 1.4 ×10^{-3} M TBAX solution containing receptor **1**, receptor **2** or receptor **3** at the same concentration as in cuvette, were added and a spectrum was acquired after each addition. The resulting titration data were analyzed using BindFit (v0.5) package, available online at http://supramolecular.org. Each titration was carried out in duplicate. Reported values are calculated as weighted arithmetic mean, where the weights were the errors obtained for each value separately. The given uncertainty of the association constants is the largest of the variance (external or internal).

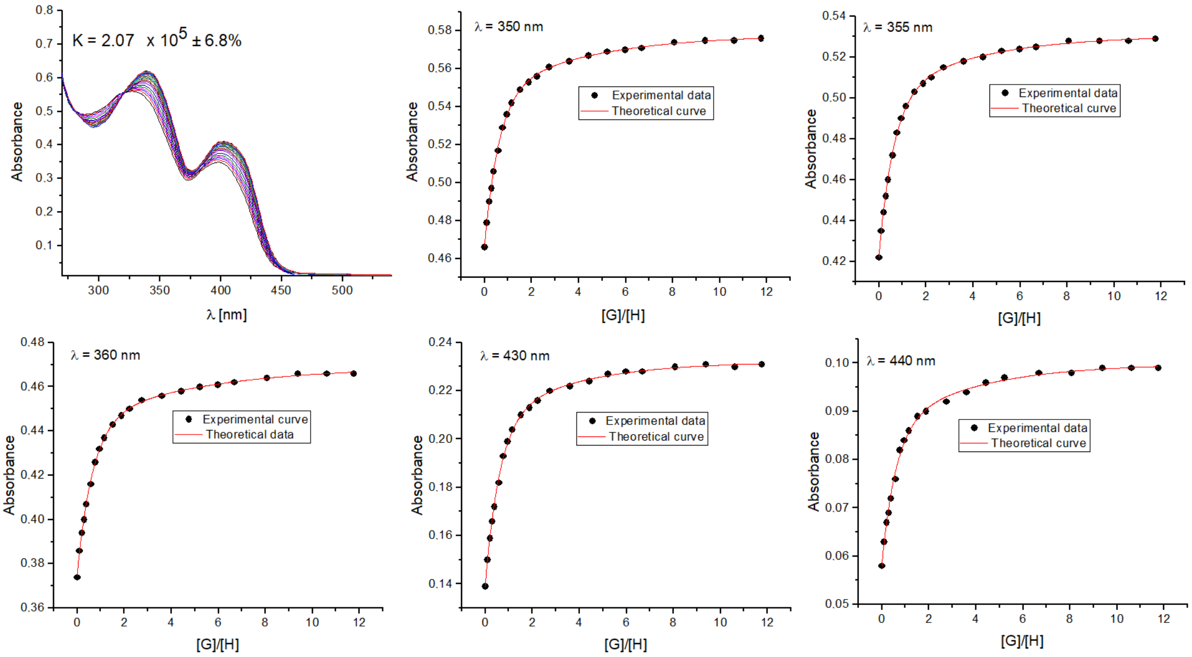

Fig. S20. UV-vis titration of Receptor **1** with TBACl in CH_3CN and selected binding isotherms.

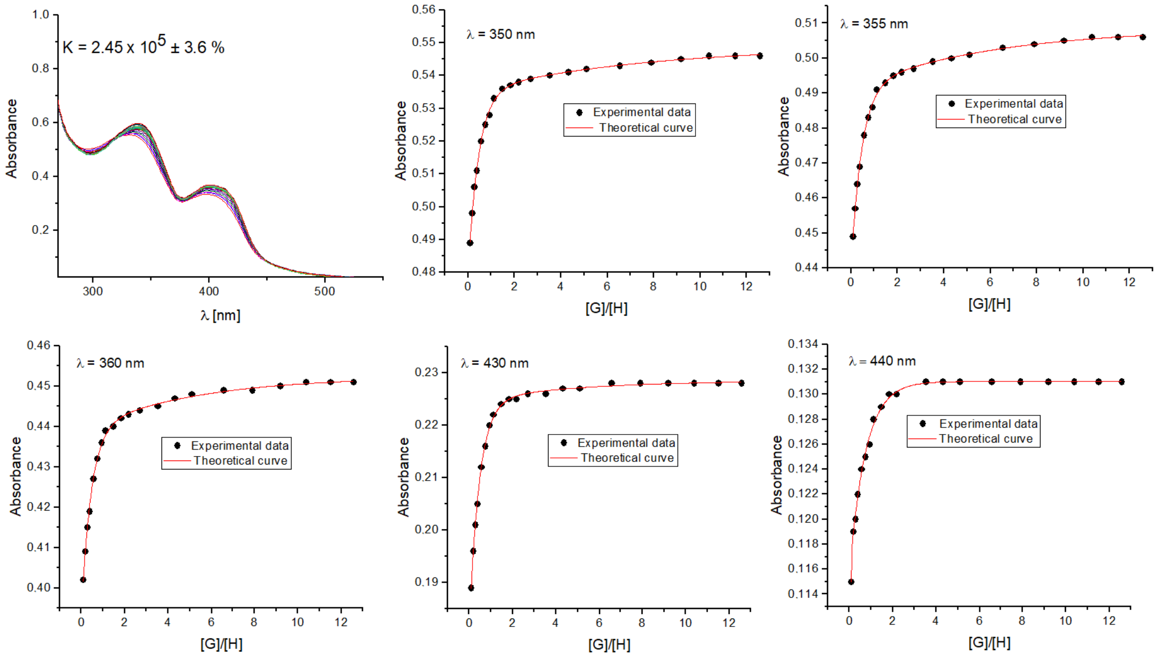

Fig. S21. UV-vis titration of Receptor **1** with TBACl in the presence of 1 equivalent of $NaClO_4$ in CH_3CN and selected binding isotherms.

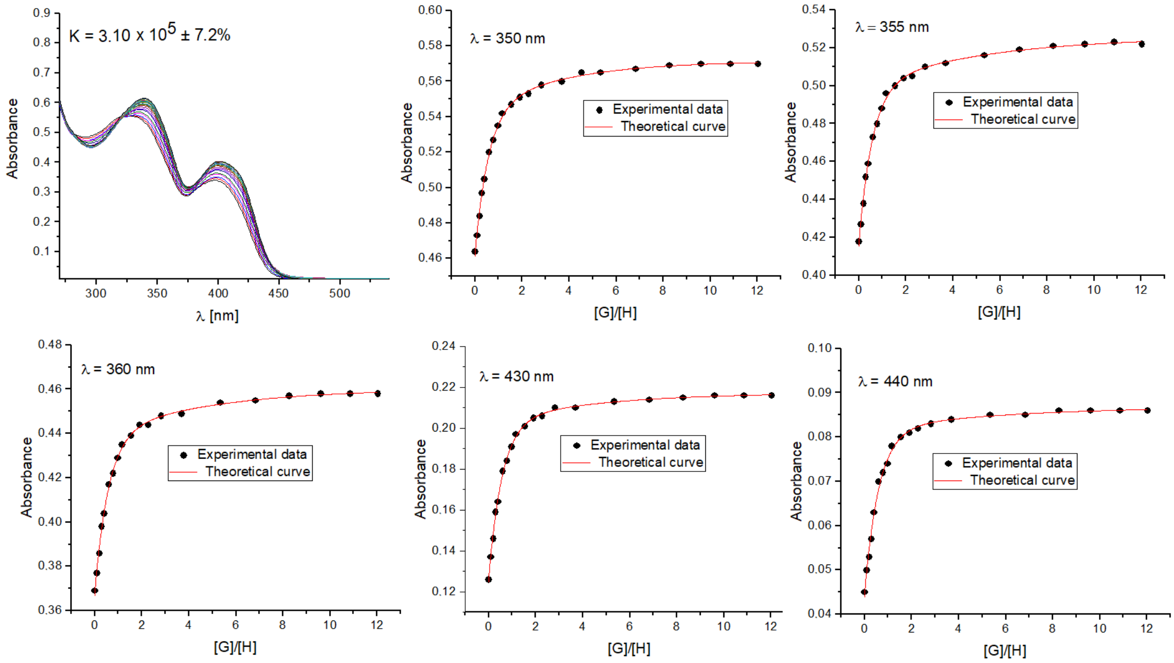

Fig. S22. UV-vis titration of Receptor **1** with TBACl in the presence of 1 equivalent of KPF_6 in CH_3CN and selected binding isotherms.

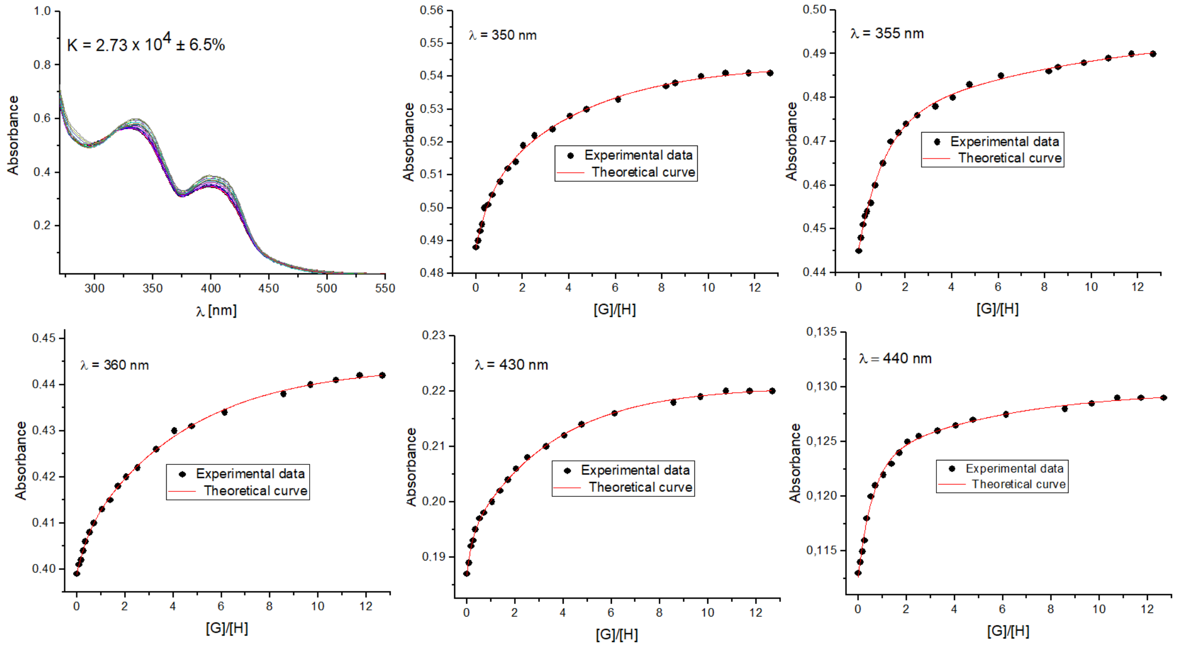

Fig. S23. UV-vis titration of Receptor **1** with TBABr in CH_3CN and selected binding isotherms.

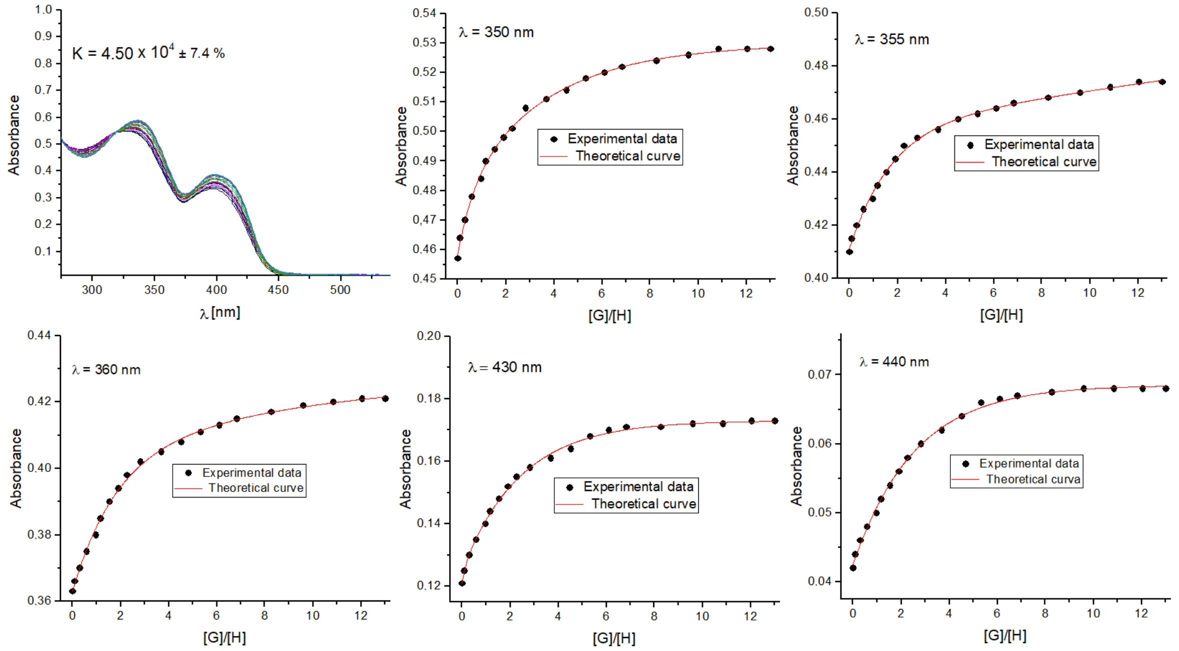

Fig. S24. UV-vis titration of Receptor **1** with TBABr in the presence of 1 equivalent of KPF_6 in CH_3CN and selected binding isotherms.

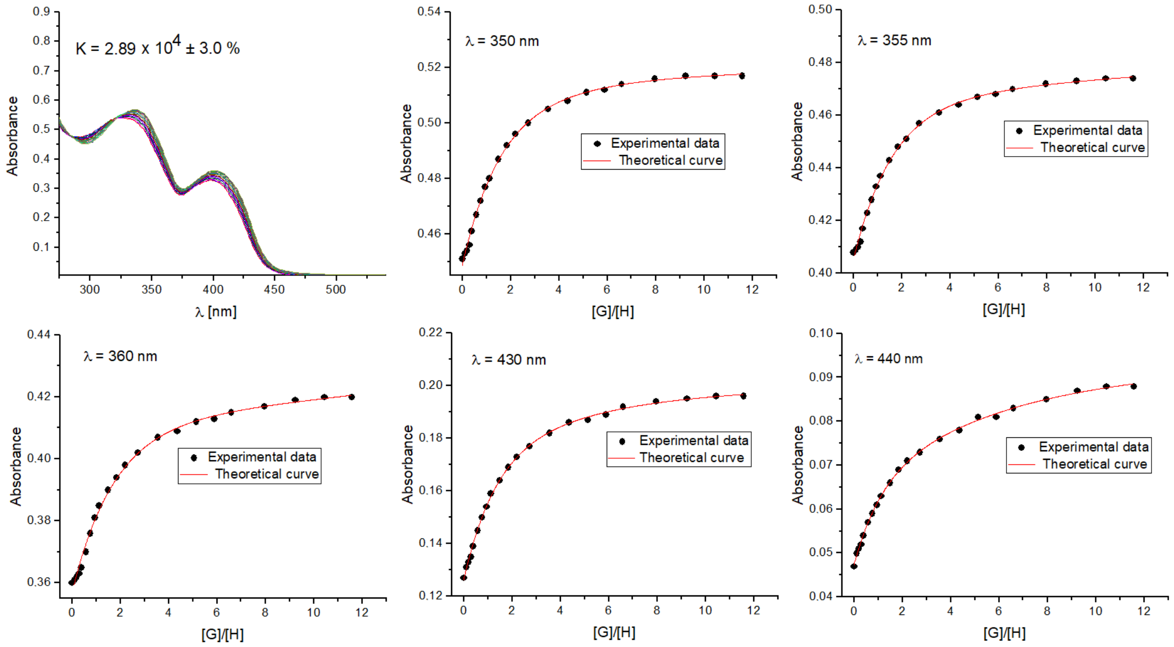

Fig. S25. UV-vis titration of Receptor **1** with $TBANO_2$ in CH_3CN and selected binding isotherms.

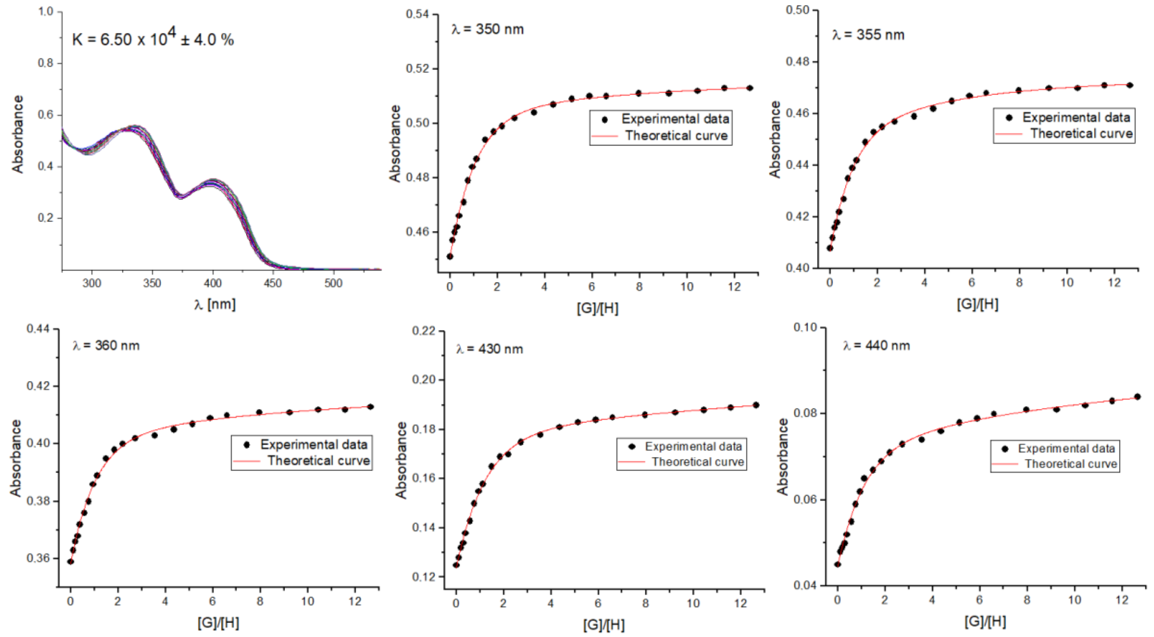

Fig. S26. UV-vis titration of Receptor **1** with $TBANO_2$ in the presence of 1 equivalent of KPF_6 in CH_3CN and selected binding isotherms.

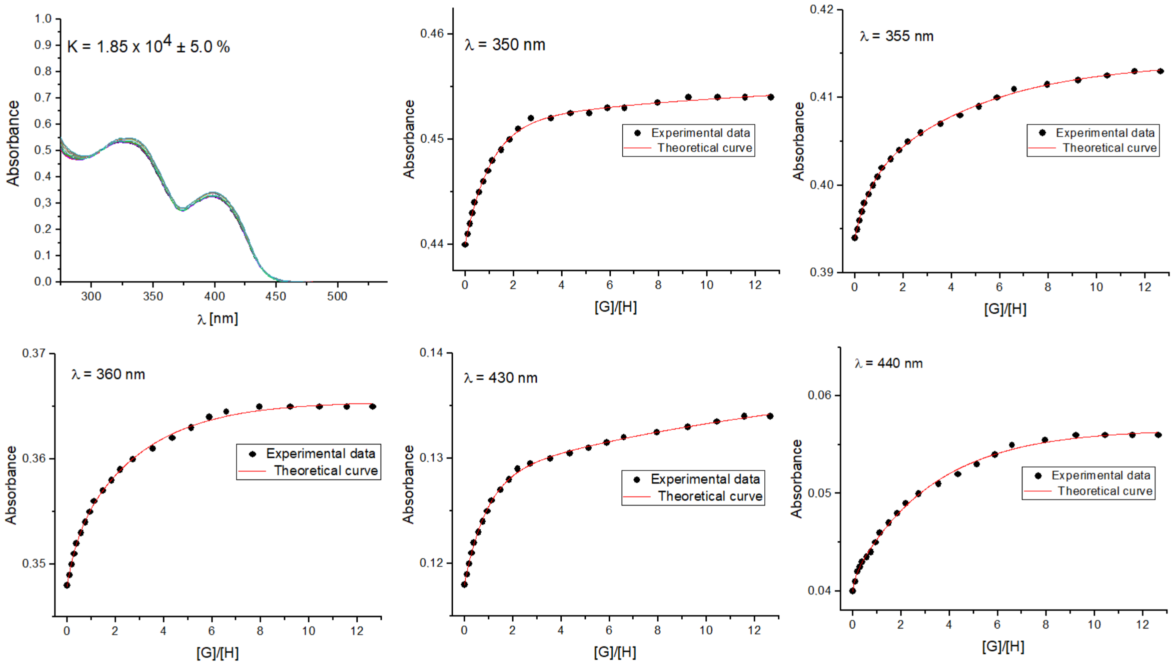

Fig. S27. UV-vis titration of Receptor **1** with $TBANO_3$ in CH_3CN and selected binding isotherms.

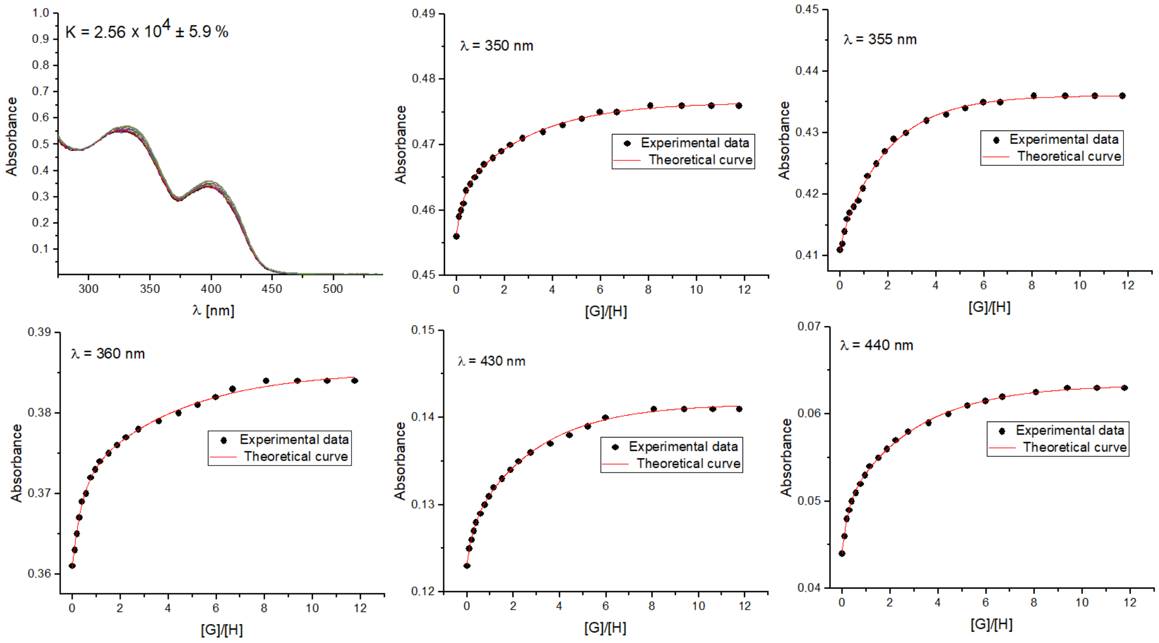

Fig. S28. UV-vis titration of Receptor **1** with $TBANO_3$ in the presence of 1 equivalent of KPF_6 in CH_3CN and selected binding isotherms.

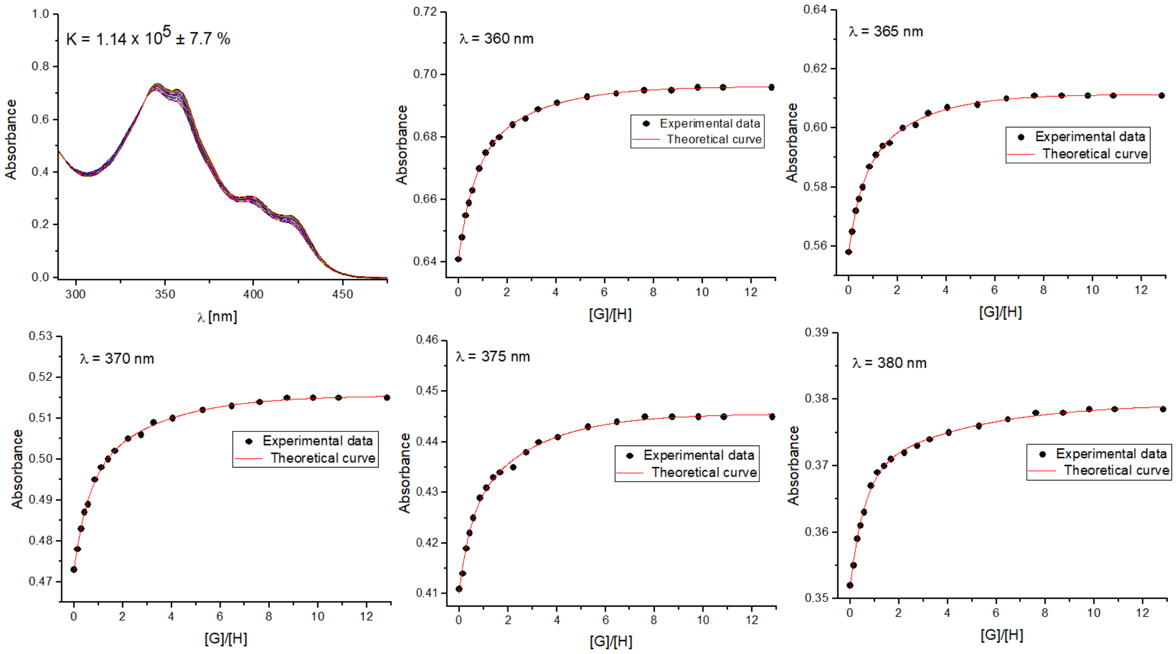

Fig. S29. UV-vis titration of Receptor **2** with TBACl in CH_3CN and selected binding isotherms.

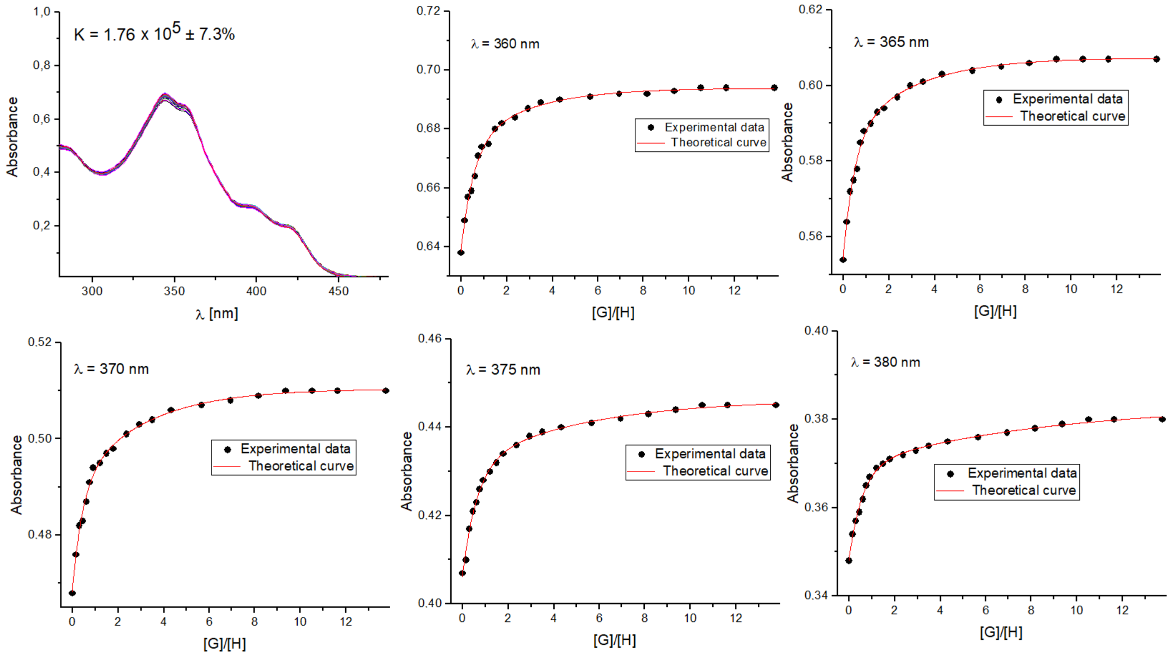

Fig. S30. UV-vis titration of Receptor **2** with TBACl in the presence of 1 equivalent of $NaClO_4$ in CH_3CN and selected binding isotherms.

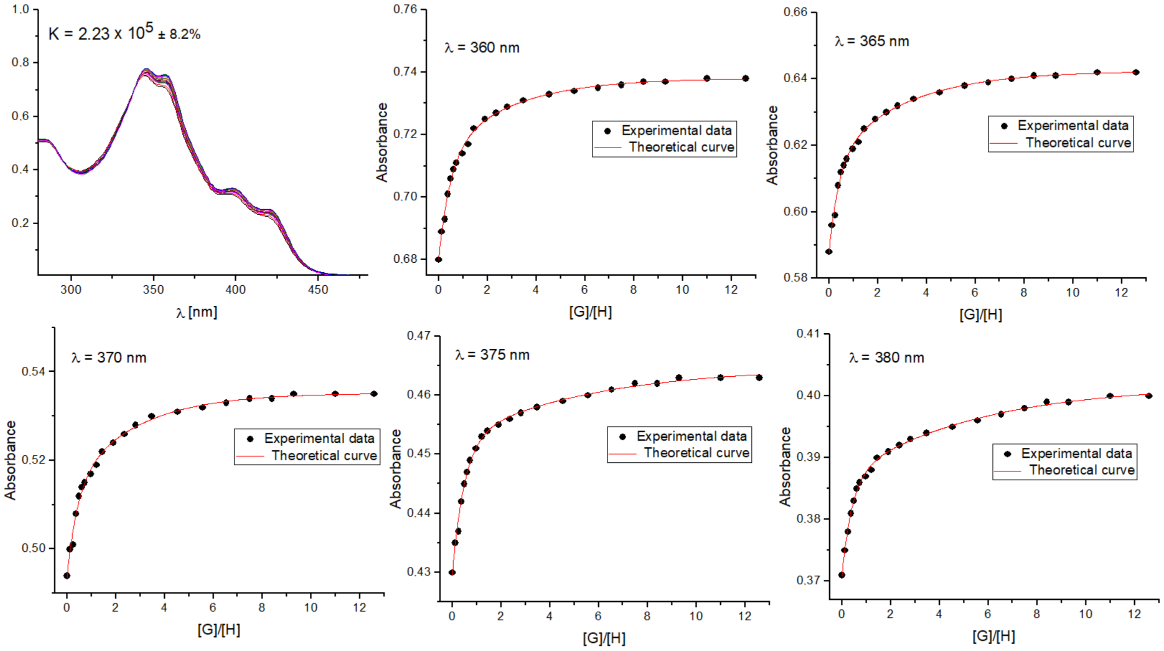

Fig. S31. UV-vis titration of Receptor **2** with TBACl in the presence of 1 equivalent of KPF_6 in CH_3CN and selected binding isotherms.

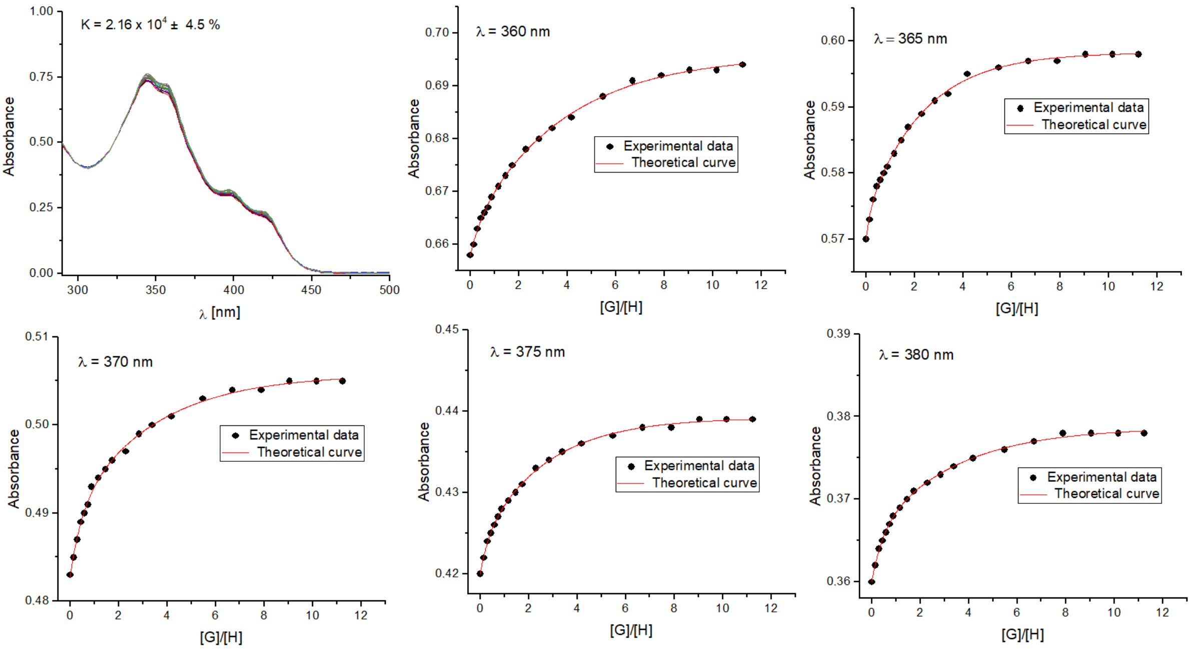

Fig. S32. UV-vis titration of Receptor **2** with TBABr in CH_3CN and selected binding isotherms.

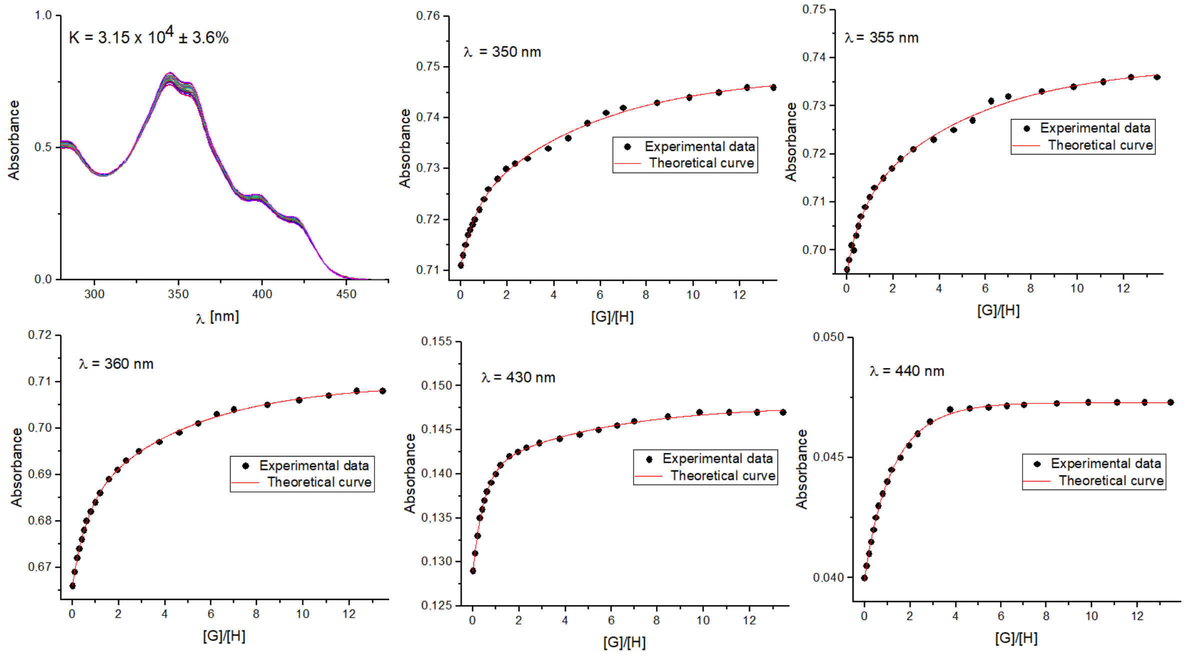

Fig. S33. UV-vis titration of Receptor **2** with TBABr in the presence of 1 equivalent of KPF_6 in CH_3CN and selected binding isotherms.

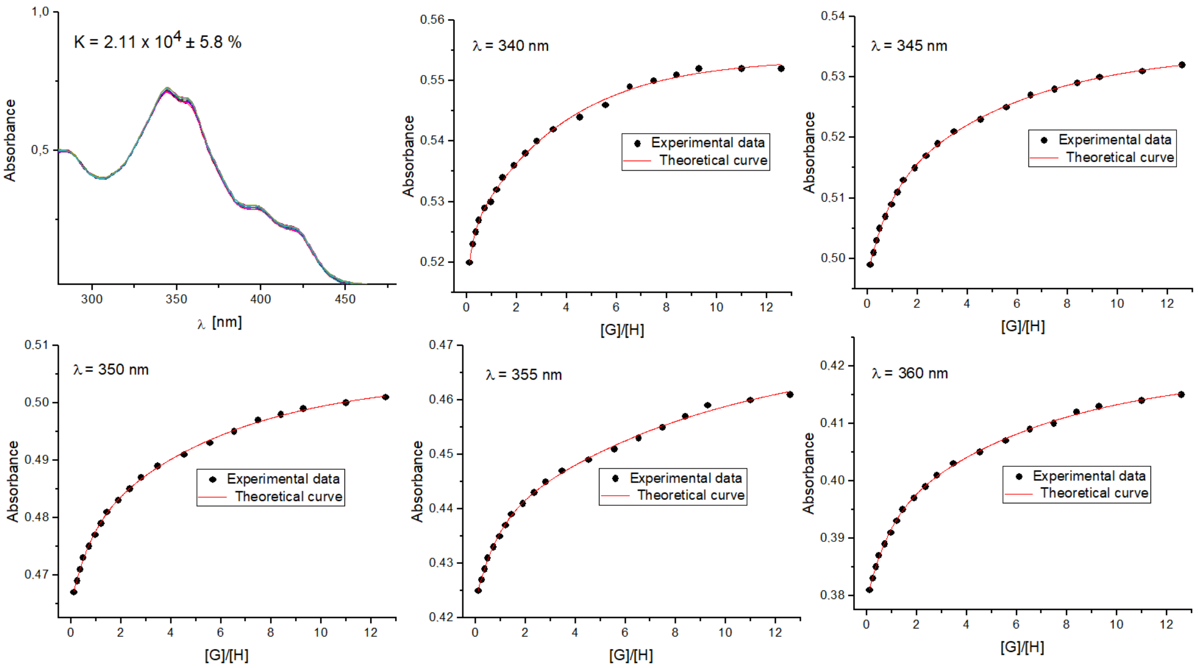

Fig. S34. UV-vis titration of Receptor **2** with $TBANO_2$ in CH_3CN and selected binding isotherms.

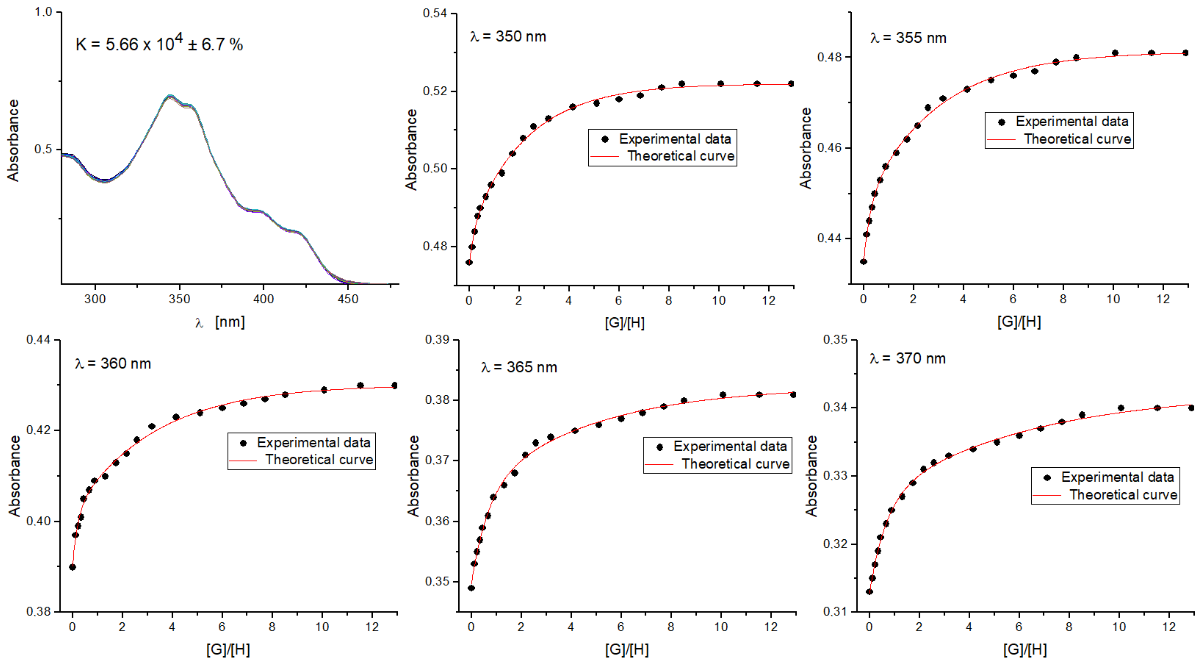

Fig. S35. UV-vis titration of Receptor **2** with $TBANO_2$ in the presence of 1 equivalent of KPF_6 in CH_3CN and selected binding isotherms.

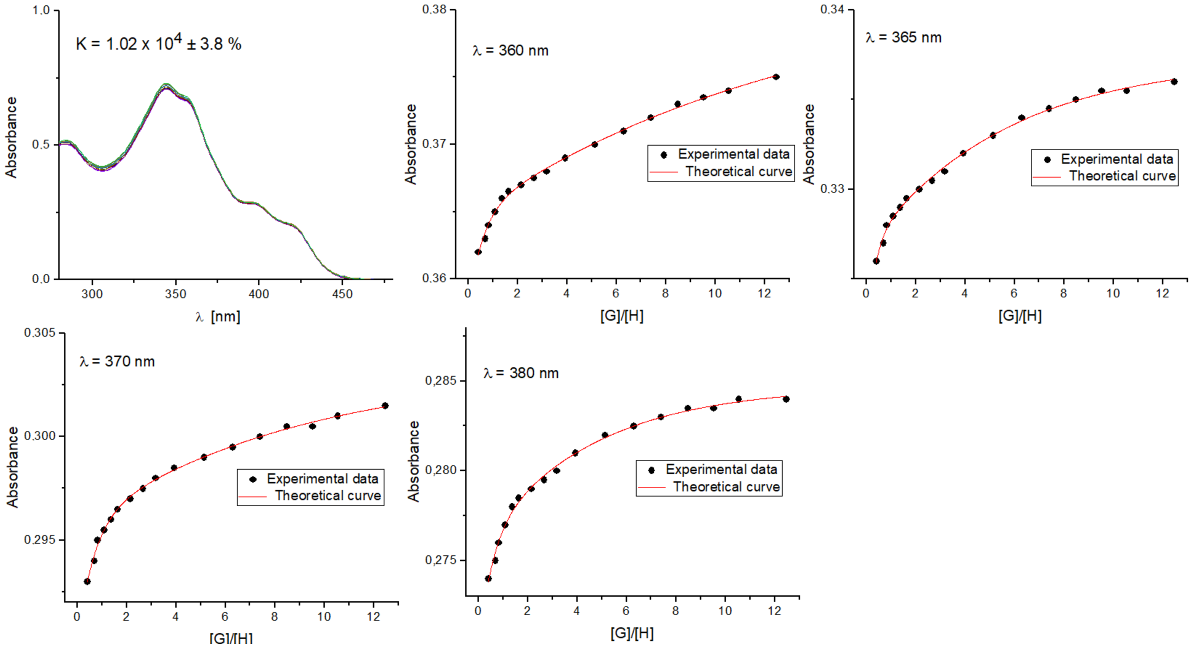

Fig. S36. UV-vis titration of Receptor **2** with $TBANO_3$ in CH_3CN and selected binding isotherms.

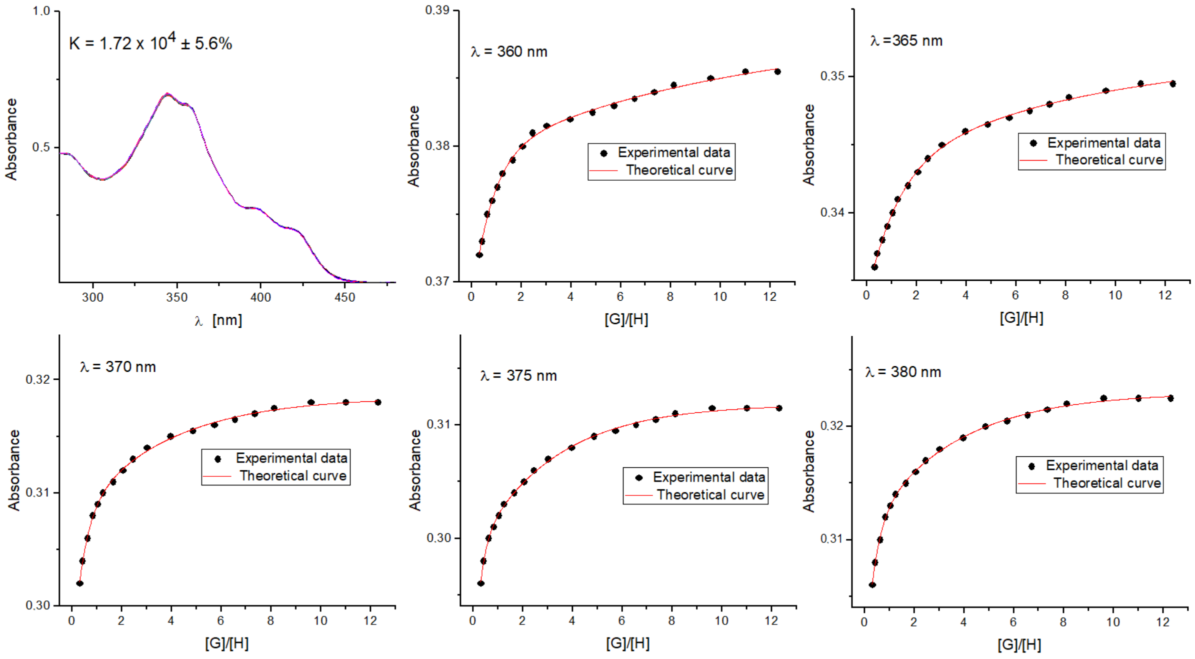

Fig. S37. UV-vis titration of Receptor **2** with $TBANO_3$ in the presence of 1 equivalent of KPF_6 in CH_3CN and selected binding isotherms.

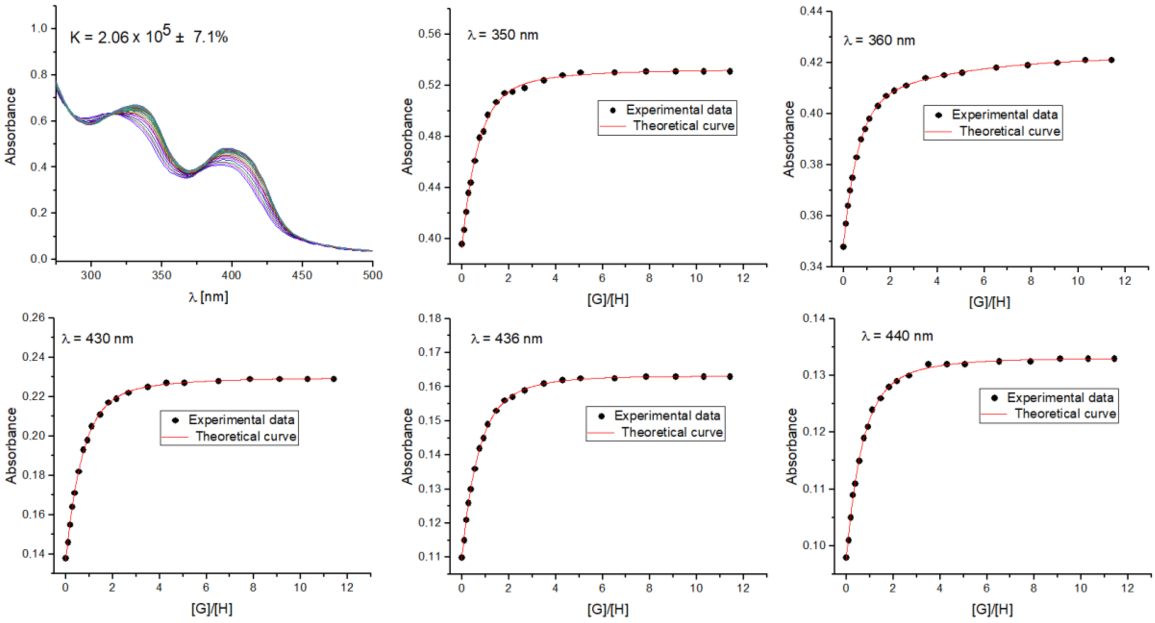

Fig. S38. UV-vis titration of Receptor **3** with TBACl in CH_3CN and selected binding isotherms.

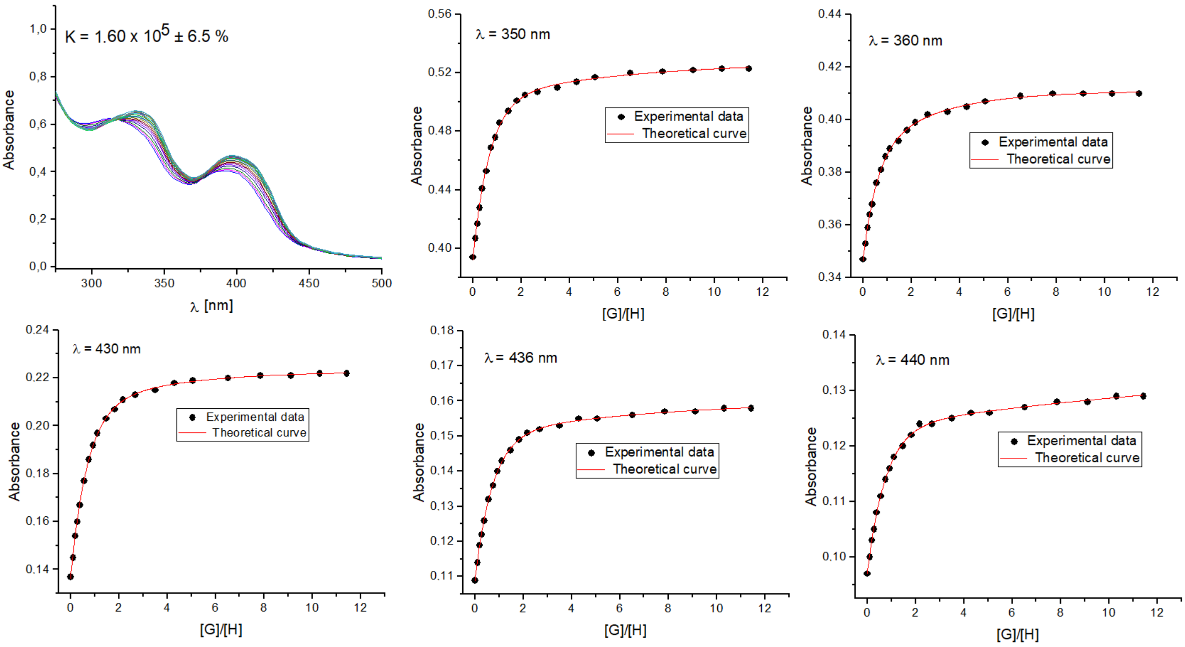

Fig. S39. UV-vis titration of Receptor **3** with TBACl in the presence of 1 equivalent of $NaClO_4$ in CH_3CN and selected binding isotherms.

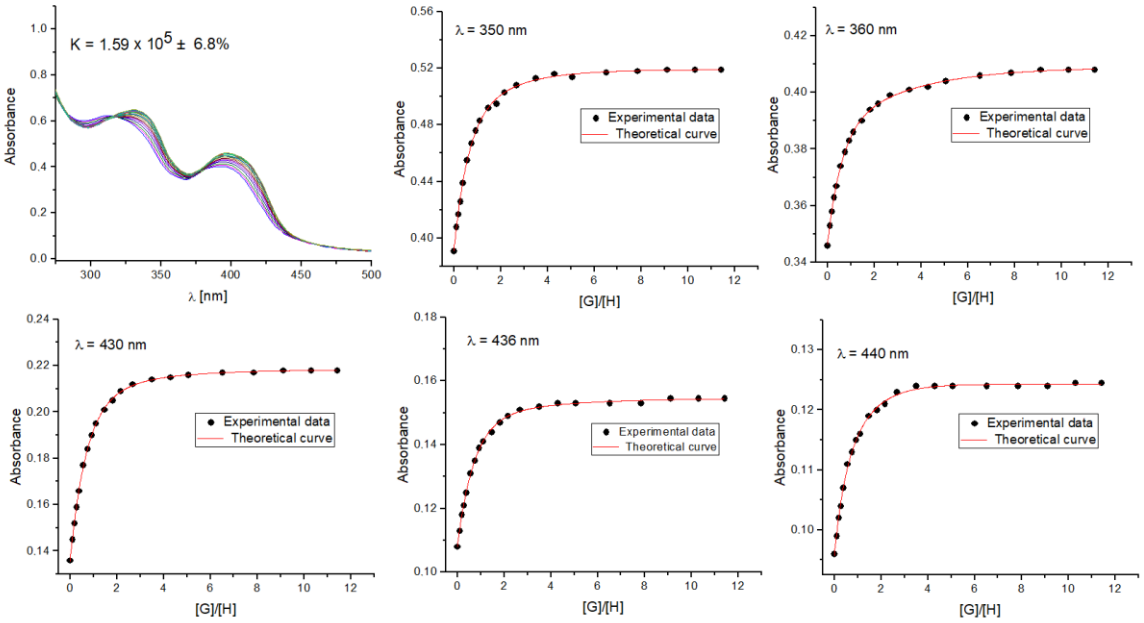

Fig. S40. UV-vis titration of Receptor **3** with TBACl in the presence of 1 equivalent of KPF_6 in CH_3CN and selected binding isotherms.

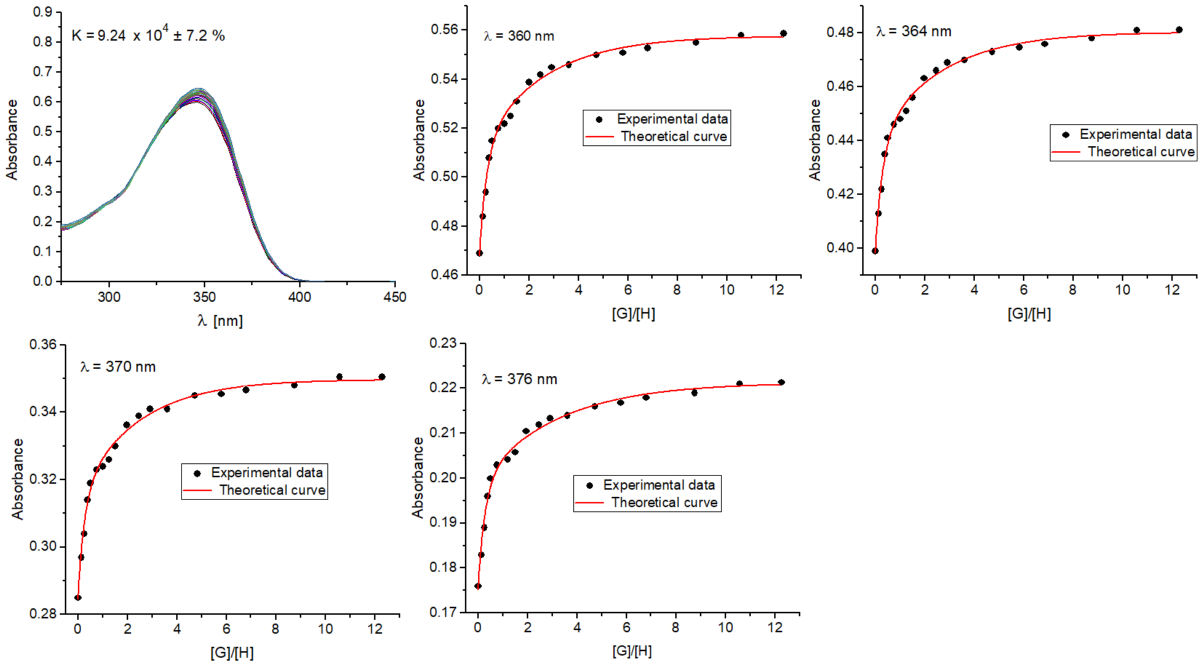

Fig. S41. UV-vis titration of Receptor **4** with TBACl in CH_3CN and selected binding isotherms.

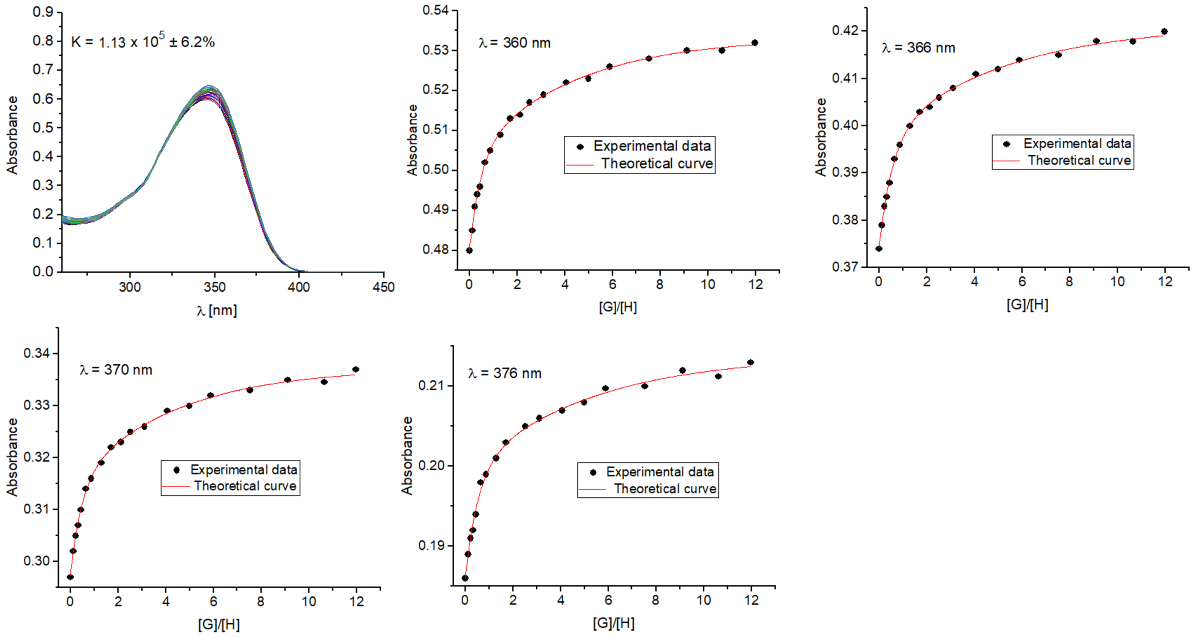

Fig. S42. UV-vis titration of Receptor **4** with TBACl in the presence of 1 equivalent of $NaClO_4$ in CH_3CN and selected binding isotherms.

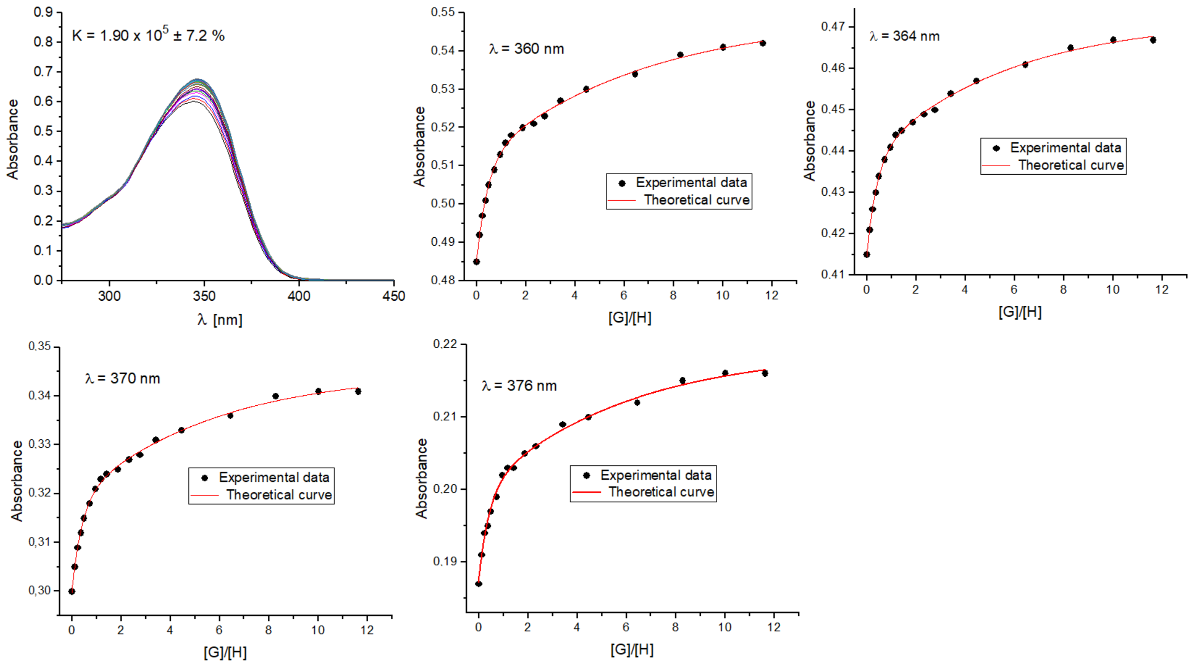

Fig. S43. UV-vis titration of Receptor **4** with TBACl in the presence of 1 equivalent of KPF_6 in CH_3CN and selected binding isotherms.

NMR titration experiments

The ^1H NMR titration was conducted at 298K in CD_3CN. In each case, a 500 μL of freshly prepared 1.6 mM solution of Receptor **1** (1.4 mM of Receptor **2**) was added to a 5 mm NMR tube. In the case of ion pair titration, the receptor was firstly pretreated with one equivalent of KPF_6. Then small aliquots of solution of TBAX, containing receptor at constant concentration, were added and a spectrum was acquired after each addition. The resulting titration data were analyzed using BindFit (v0.5) package, available online at http://supramolecular.org.

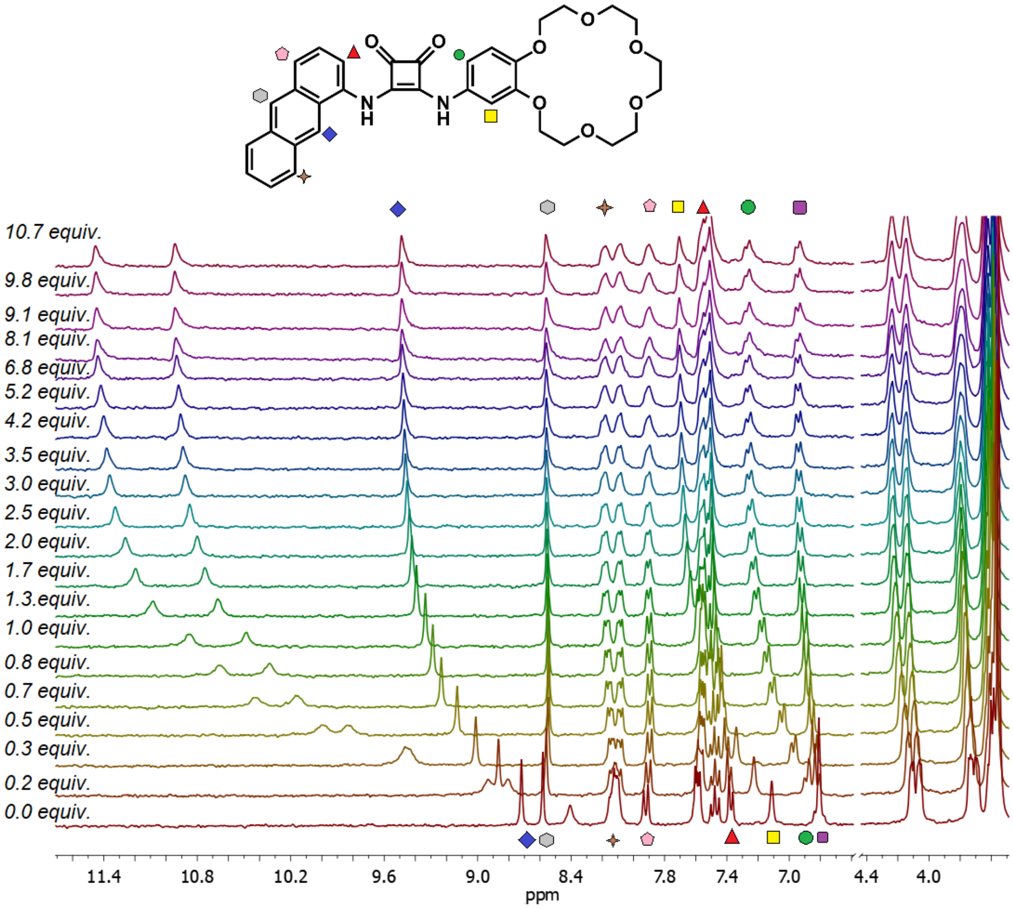

Fig. S44. Partial ^{1}HNMR spectra recorded upon titration of **1** in CD_3CN with TBABr.

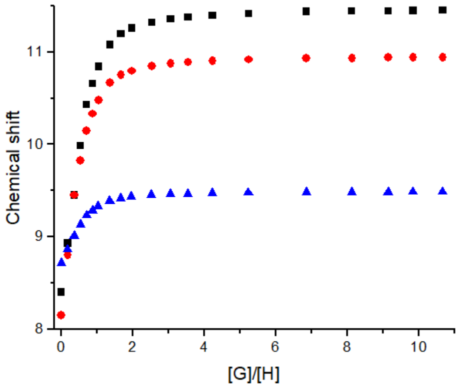

Fig. S45. ^{1}H NMR titration binding isotherms of **1** in CD_3CN upon addition of increasing amounts of TBABr.

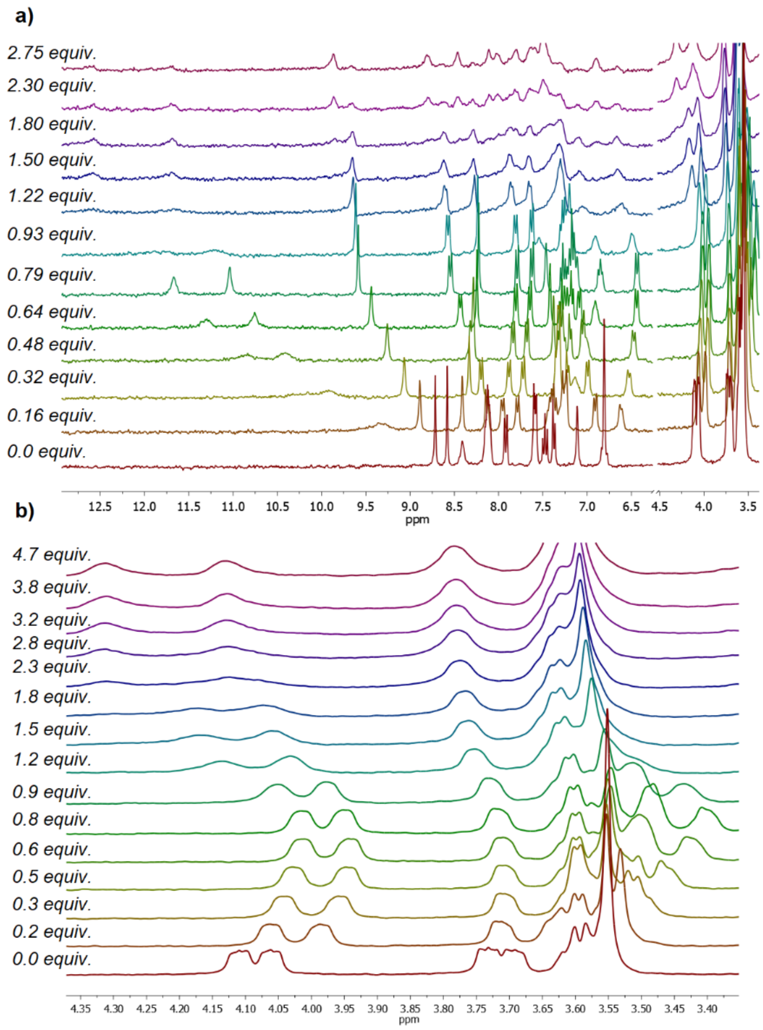

Fig. S46. ^{1}H NMR spectra recorded upon titration of receptor **1** in CD_3CN with TBA_2SO_4 titration performed in the range from 0.0 equiv. to 2.75 equiv. - a) (signals corresponding to the crown ether - b).

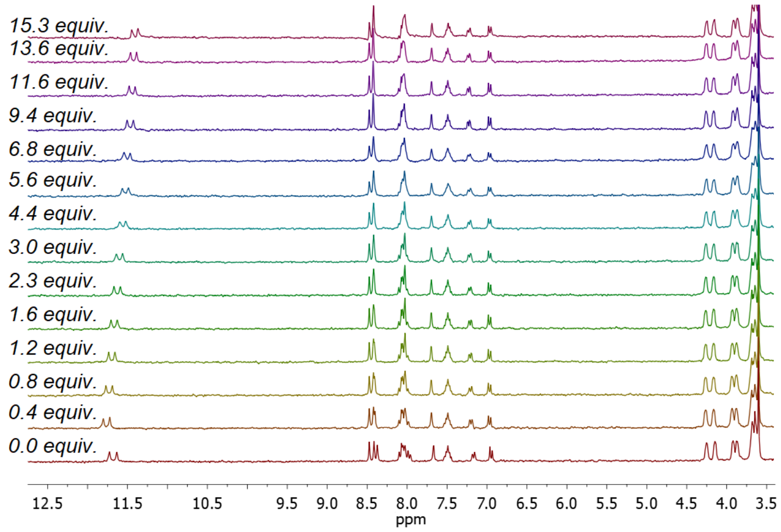

Fig. S47. Partial ^{1}HNMR spectra were recorded upon titration of **2** in CD_3CN with TBABr.

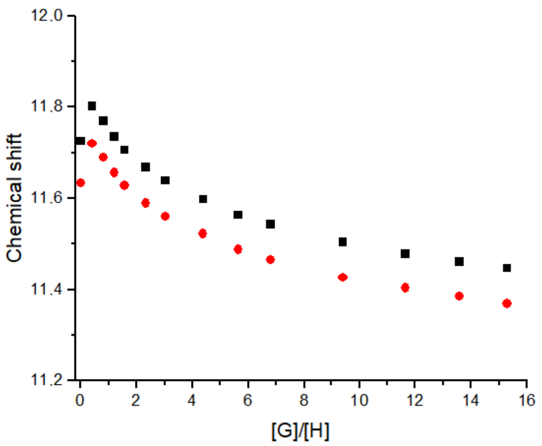

Fig. S48. ^{1}H NMR titration binding isotherms of **2** in CD_3CN upon addition of increasing amounts of TBABr.

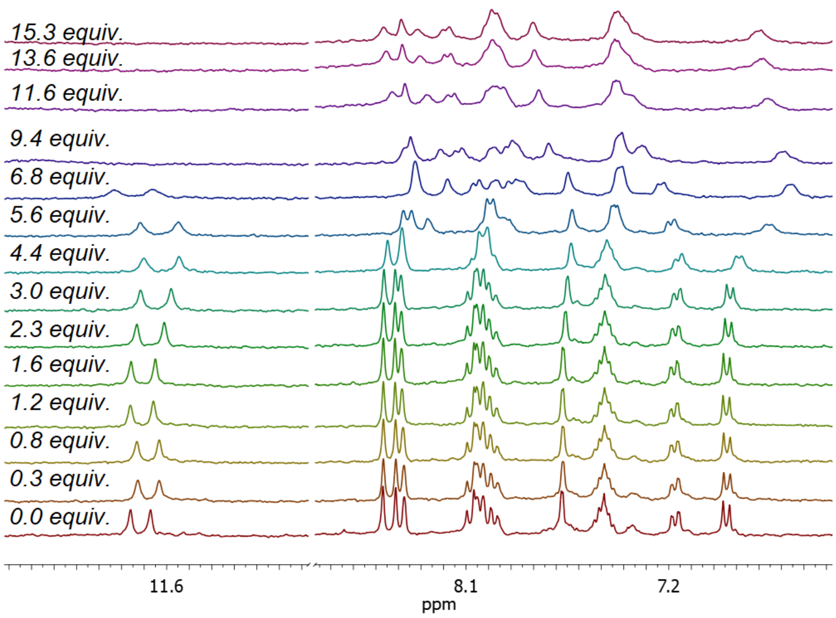

Fig. S49. Partial ^{1}HNMR spectra were recorded upon titration of **2** in CD_3CN with TBA_2SO_4 (signals corresponding to amide and phenyl protons).

^{1}H DOSY and 2D experiments

Table S1. Diffusion coefficients during dilutions for Receptors **1** and **2** in CD_3CN.

c [mM]	Receptor 1	Receptor 2
**3.9**	0.954 e-5 (1e-7) cm^2s^{-1}	0.984e-5 (1e-7) cm^2s^{-1}
**1.2**	1.000 e-5 (2e-7) cm^2s^{-1}	1.014e-5 (2e-7) cm^2s^{-1}
**0.6**	1.035 e-5 (3e-7) cm^2s^{-1}	1.031e-5 (3e-7) cm^2s^{-1}

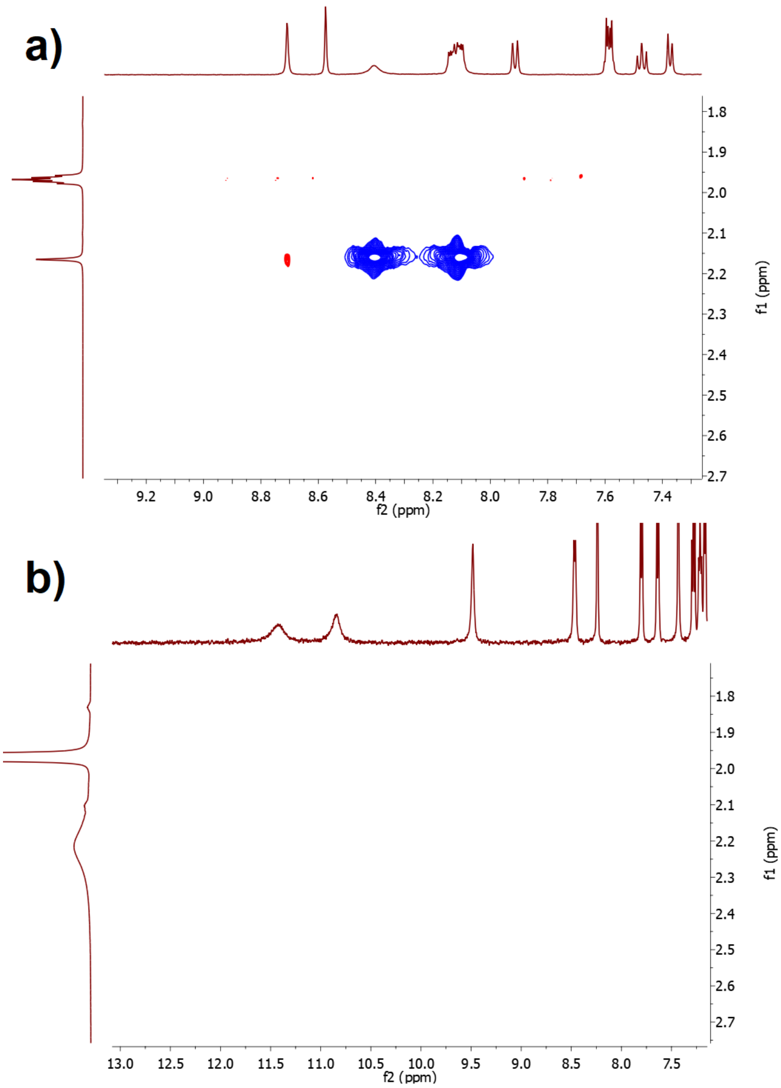

Fig. S50. ROESY NMR spectrum of receptor **1** (2.4 mM) in CD_3CN (a) and after addition of 0.6 equiv. TBA_2SO_4 (b).

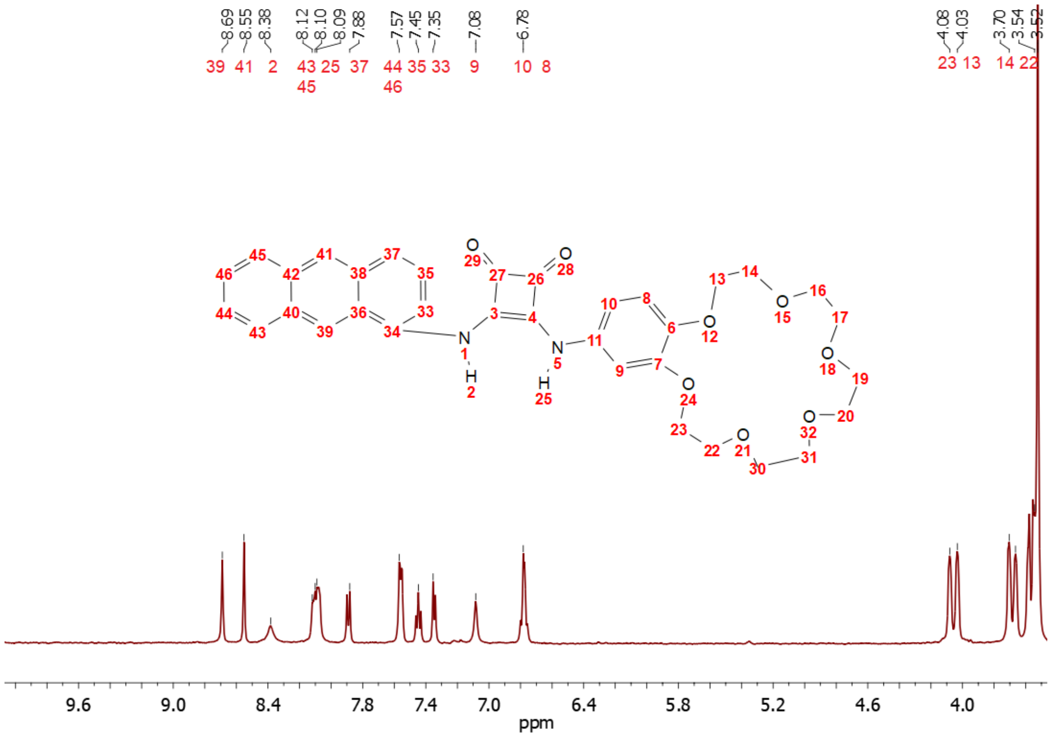

Fig. S51. ^{1}H NMR spectrum of Receptor **1** (3.9 mM) in CD_3CN. Individual signals were assigned based on 2D NMR spectra.

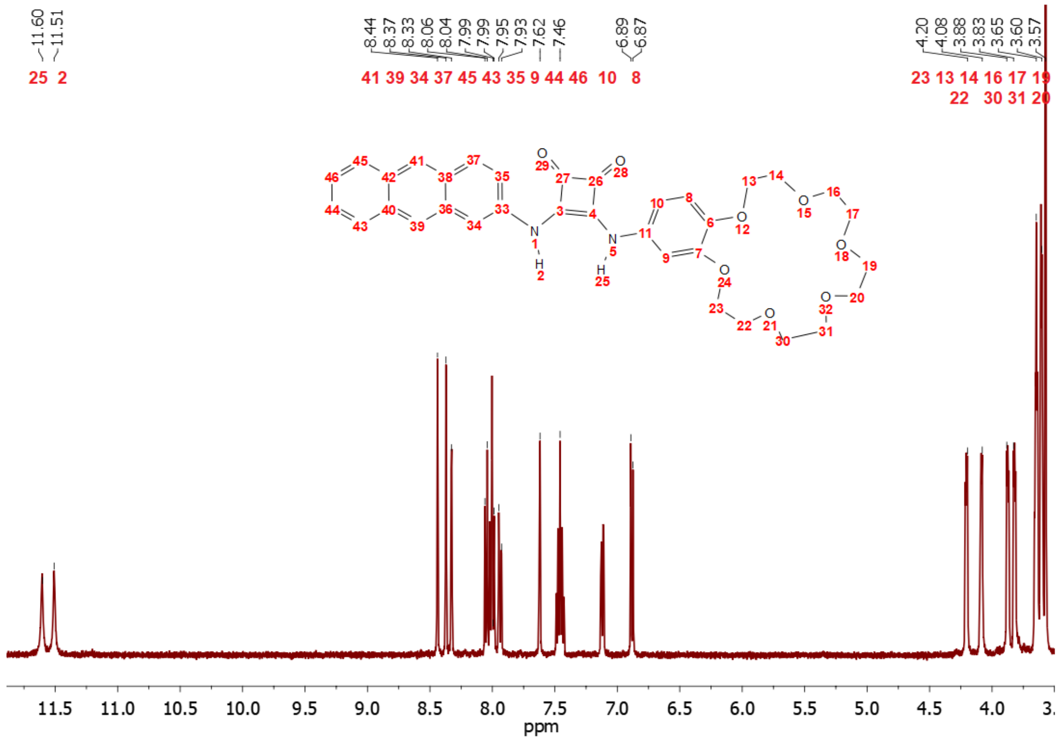

Fig. S52. ^{1}H NMR spectrum of Receptor **2** (3.9 mM) in CD_3CN. Individual signals were assigned based on 2D NMR spectra.

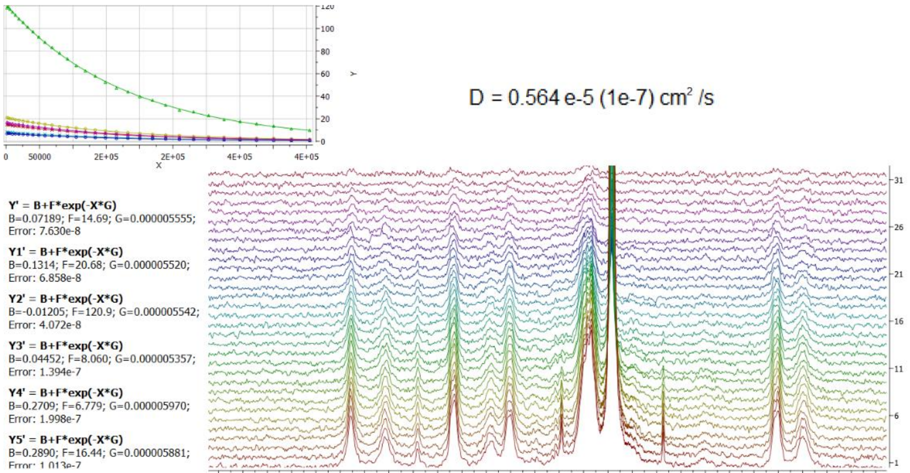

Fig. S53. DOSY NMR spectrum of receptor **1** (2.0 mM) in wet $CDCl_3$.

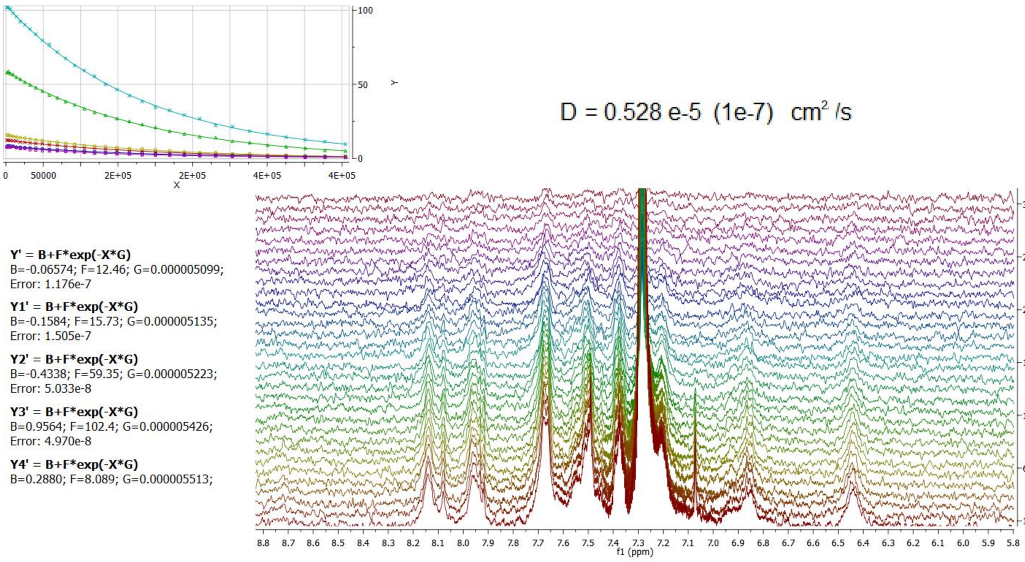

Fig. S54. DOSY NMR spectrum of receptor **1** (2.0 mM) in wet $CDCl_3$ after extraction of aq. solution of KCl.

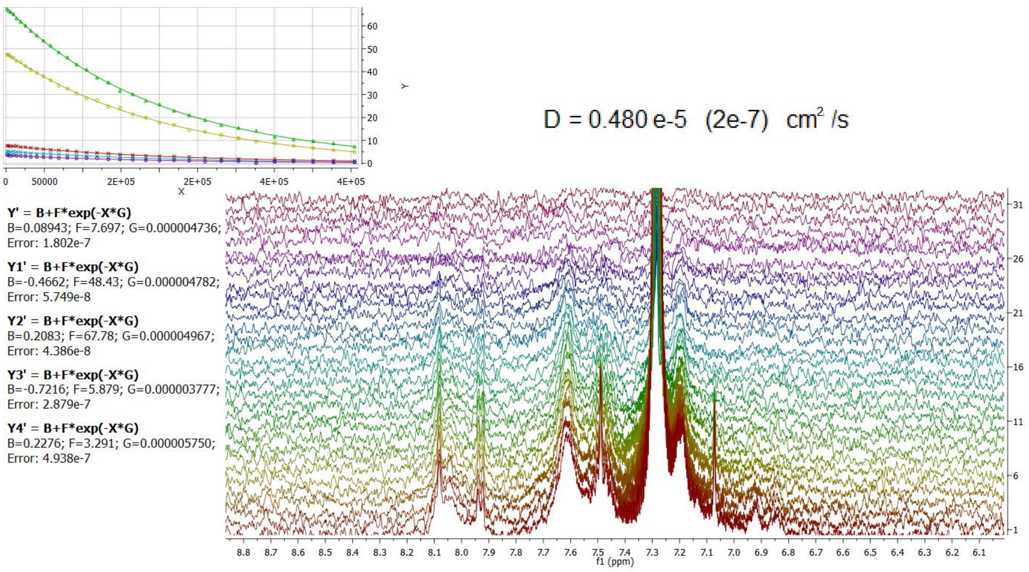

Fig. S55. DOSY NMR spectrum of receptor **1** (2.0 mM) in wet $CDCl_3$ after extraction of aq. solution of K_2SO_4.

Extraction experiments – mass spectrometry and ^{1}H NMR

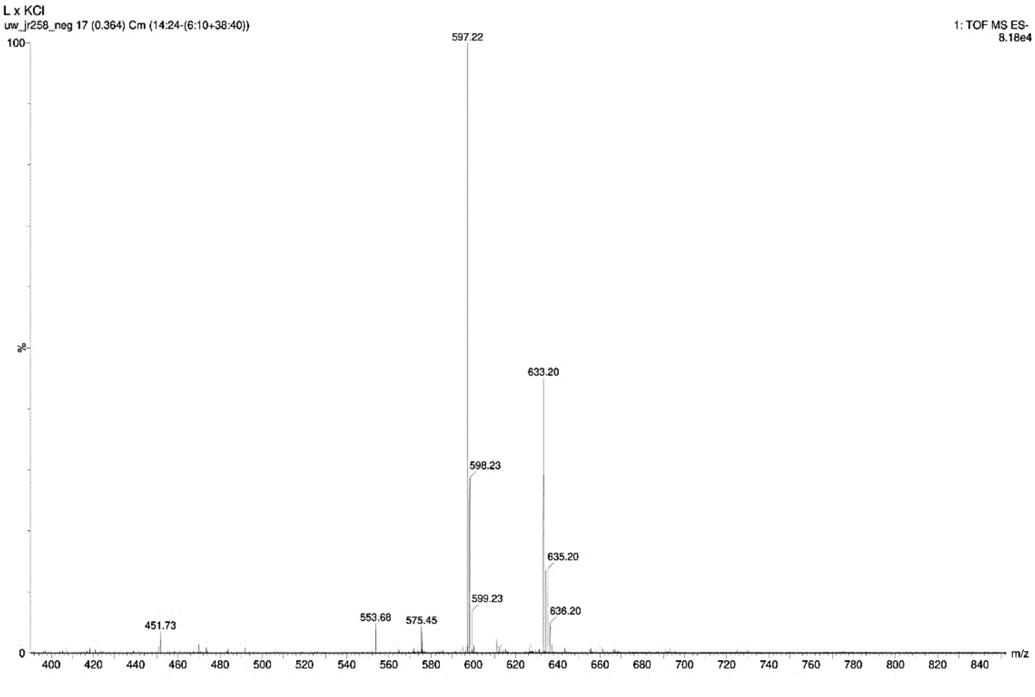

Fig. S56. MS of CH_2Cl_2 solution of Receptor **1** after aq. KCl extraction (negative ion mode).

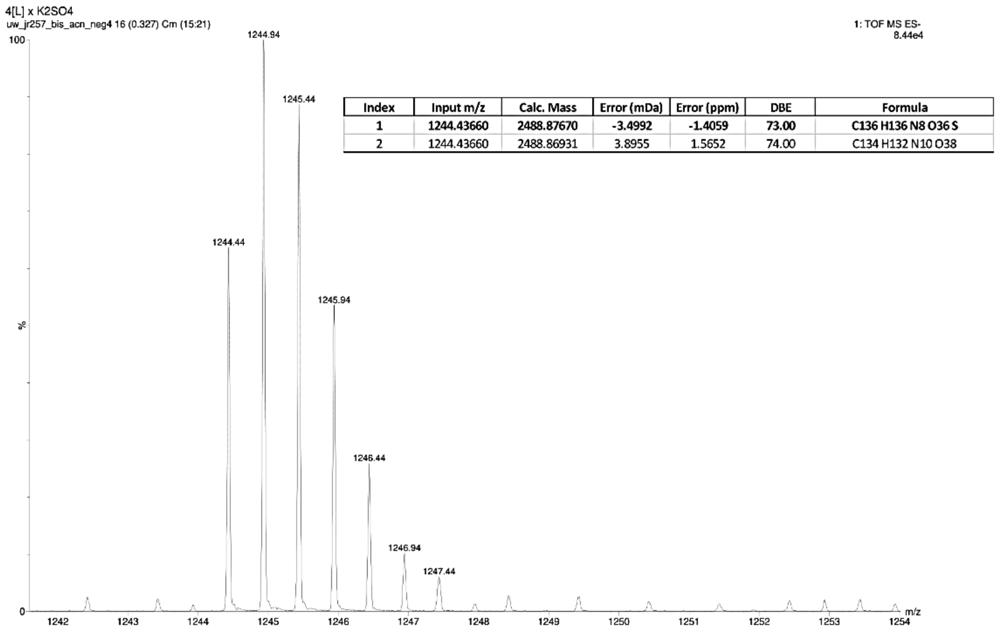

Index	Input m/z	Calc. Mass	Error (mDa)	Error (ppm)	DBE	Formula
1	1244.43660	2488.87670	-3.4992	-1.4059	73.00	C136 H136 N8 O36 S
2	1244.43660	2488.86931	3.8955	1.5652	74.00	C134 H132 N10 O38

Fig. S57. MS of CH_2Cl_2 solution of Receptor **1** after aq. K_2SO_4 extraction (negative ion mode).

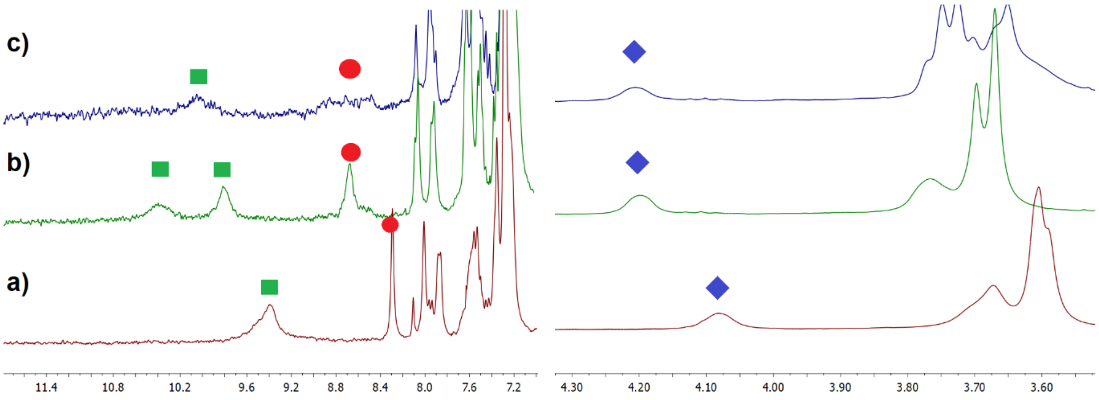

Fig. S58. Partials ^{1}H NMR spectra of Receptor **1** at 3 mM (a) in wet $CDCl_3$; (b) after KCl (50 mM) extraction from the aqueous phase; (c) after K_2SO_4 (50 mM) extraction from the aqueous phase.

Extraction experiments - ion chromatography

A solution of Receptor **1** in chloroform (2 ml, 20 mM or 5 mM) was intensive shaking with an aqueous mixture (no pH adjustment, pH depending on the salts used; above pH 8 there is no phase separation probably due to the receptor deprotonation. This eliminates direct use of basic salts such as carboxylates, hydrogen phosphates or phosphates) of suitable salts 5 mM each (2 ml) for 30 minutes. Then 1 mL of the aqueous phase was taken and tenfold diluted. The concentration of chloride, bromide, nitrite, nitrate, dihydrogenphosphate and sulfate anions in the aqueous phase was determined by high performance ion chromatography (HPIC).

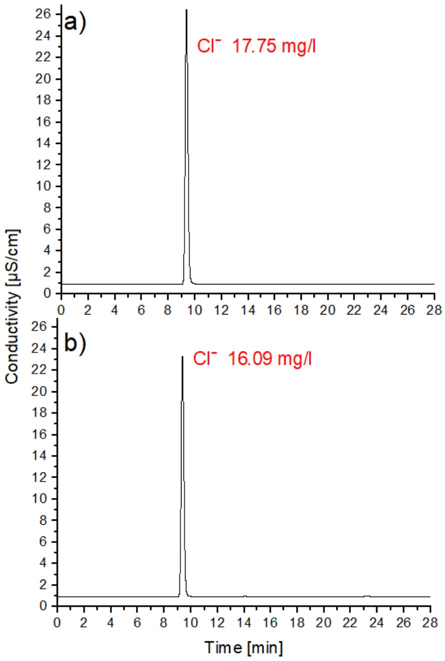

Fig. S59. The chromatogram was obtained during the extraction experiment after tenfold dilution (a) source phase - aqueous KCl salt (b) after extraction with 5 mM of Receptor **1** in $CHCl_3$.

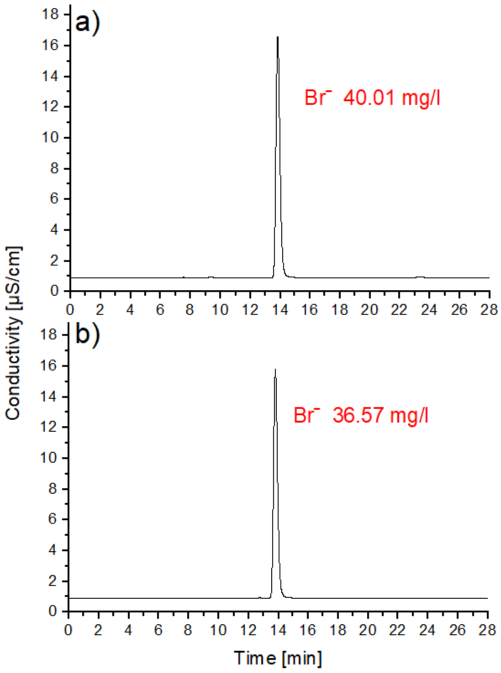

Fig. S60. The chromatogram was obtained during the extraction experiment after tenfold dilution (a) source phase - aqueous KBr salt (b) after extraction with 5 mM of Receptor **1** in $CHCl_3$.

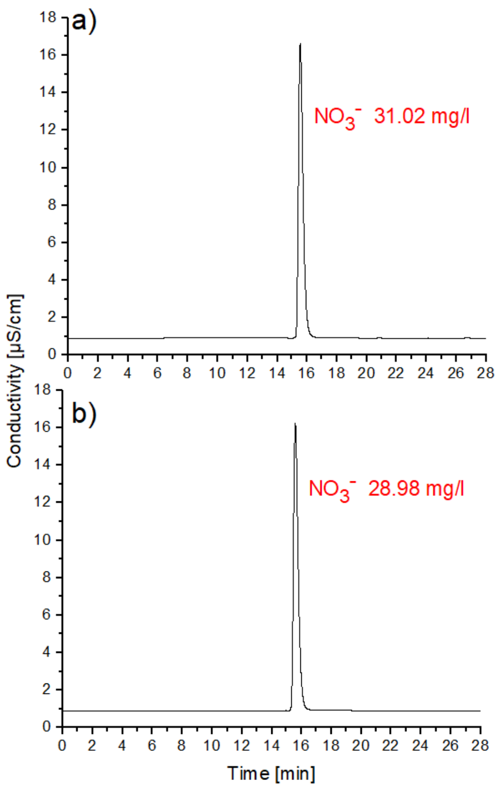

Fig. S61. The chromatogram was obtained during the extraction experiment after tenfold dilution (a) source phase - aqueous KNO_3 salt (b) after extraction with 5 mM of Receptor **1** in $CHCl_3$.

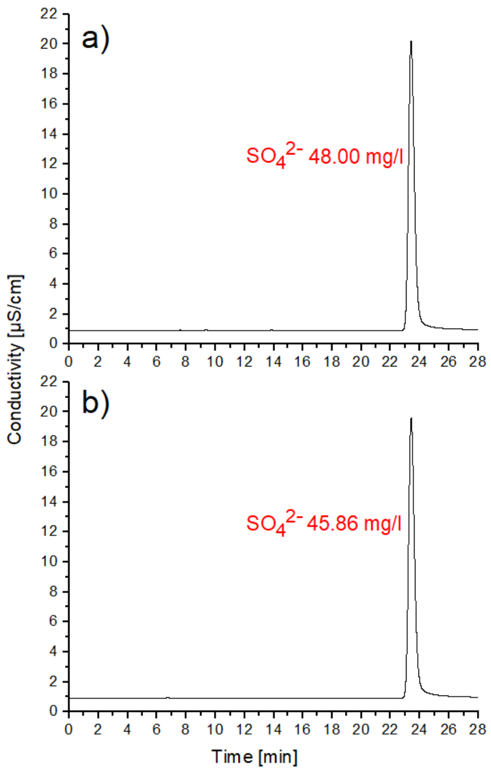

Fig. S62. The chromatogram was obtained during the extraction experiment after tenfold dilution (a) source phase - aqueous K_2SO_4 salt (b) after extraction with 5 mM of Receptor **1** in $CHCl_3$.

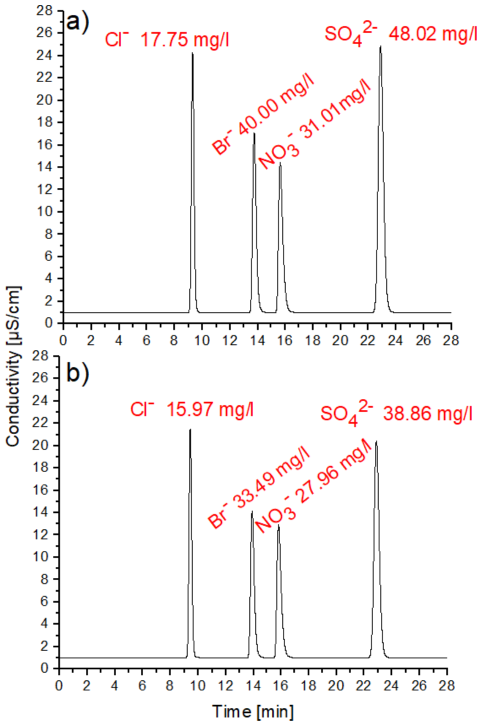

Fig. S63. Chromatograms obtained during extraction experiments after tenfold dilution (a) source phase – an aqueous mixture of potassium salts (b) after extraction with 20 mM of Receptor **1** in $CHCl_3$.

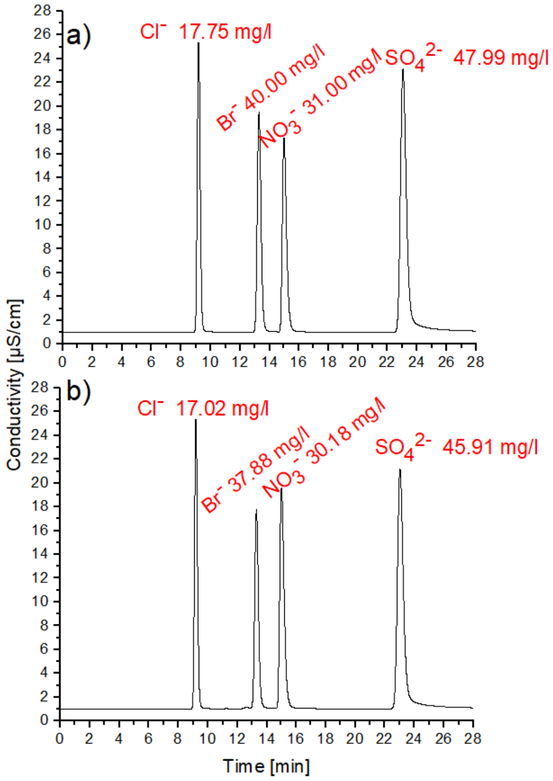

Fig. S64. Chromatograms obtained during extraction experiments after tenfold dilution (a) source phase – an aqueous mixture of sodium salts (b) after extraction with 20 mM of Receptor **1** in $CHCl_3$.

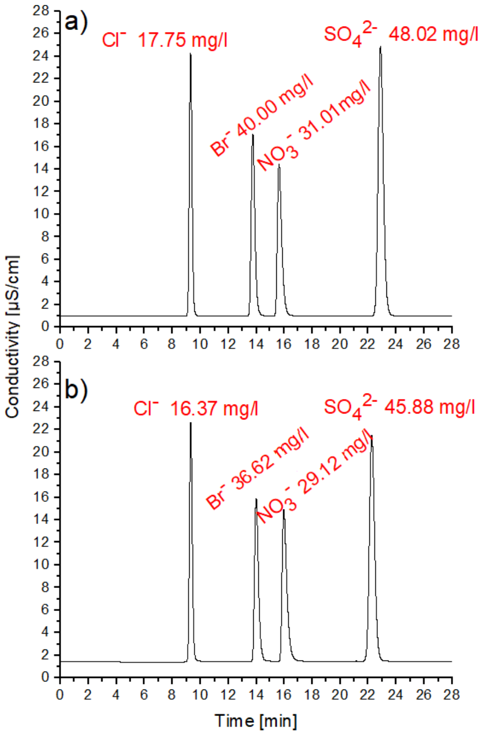

Fig. S65. Chromatograms obtained during extraction experiments after tenfold dilution (a) source phase – an aqueous mixture of potassium salts (b) after extraction with 5 mM of Receptor **1** in $CHCl_3$.

Emission spectra

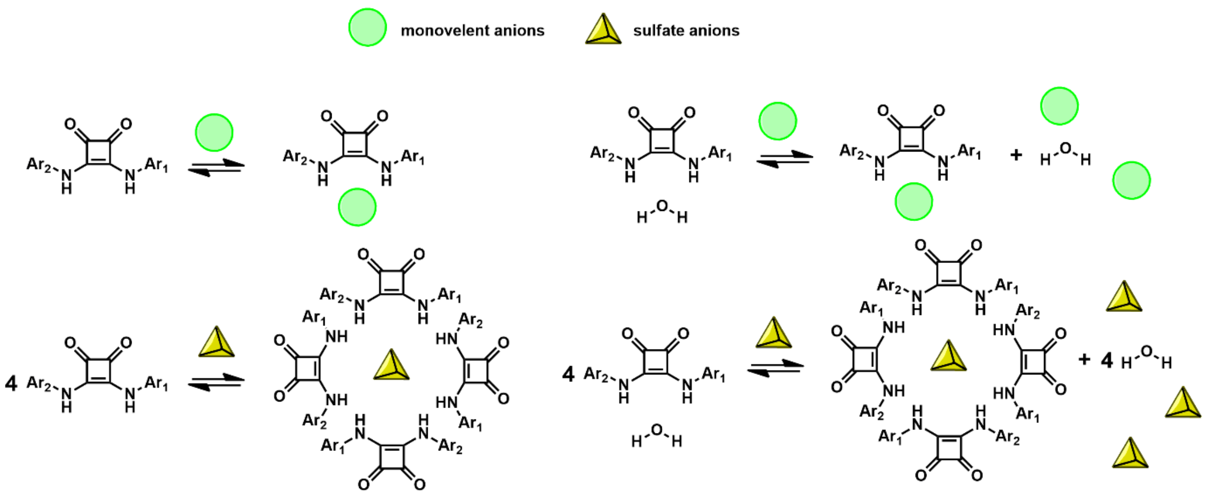

Fig. S66. Schematic illustration of the difference in binding mode by receptors.

Fluorescence emission spectra were measured on a Hitachi F-7100 Fluorescence Spectrophotometer. Solution of Receptor **1** and **2**(c=1.0 × 10^{-4} M) in CH_3CN or $CHCl_3$ were titration with small aliquots of

TBAX solution containing **1** or **2** at the same concentration as in cuvette. Successive scans were performed measuring fluorescence (λ_{ex}= 327 nm for **1** and 345 nm for **2**) emission between 300 and 700 nm.

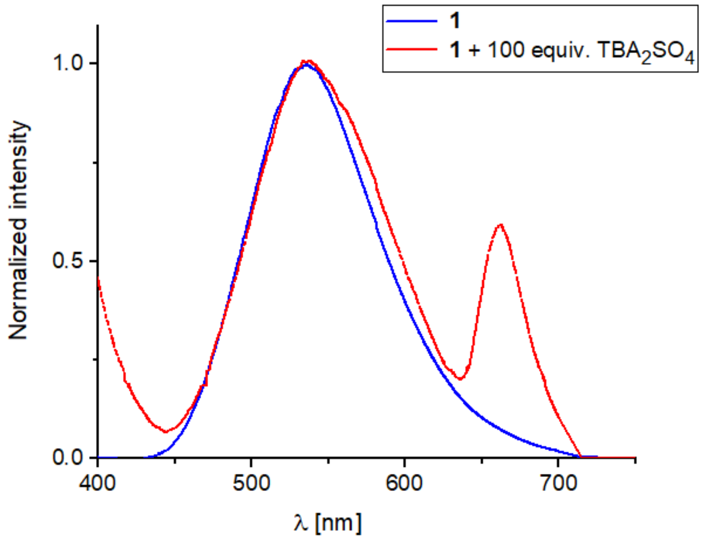

Fig. S67. Normalized spectra of Receptor **1** (1.0×10^{-4}M) in the absence and the presence of an excess of TBA_2SO_4 (excitation at 327 nm) in dry CH_3CN.

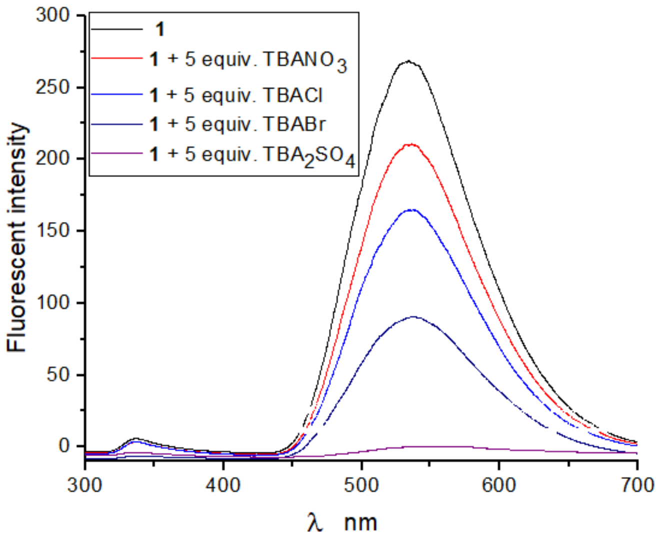

Fig. S68. Emission spectra of Receptor **1** (4.0×10^{-4}M) and upon addition of TBA salts (excitation at 327 nm) in dry CH_3CN.

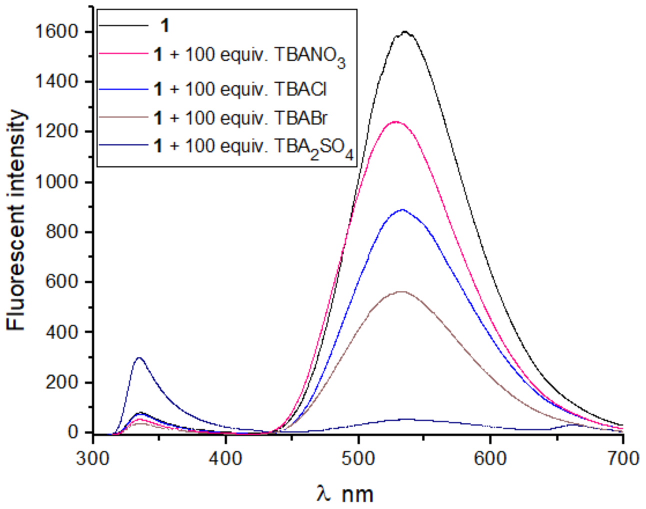

Fig. S69. Emission spectra of Receptor **1** (1.0×10^{-4}M) and upon addition of TBA salts (excitation at 327 nm) in dry CH_3CN.

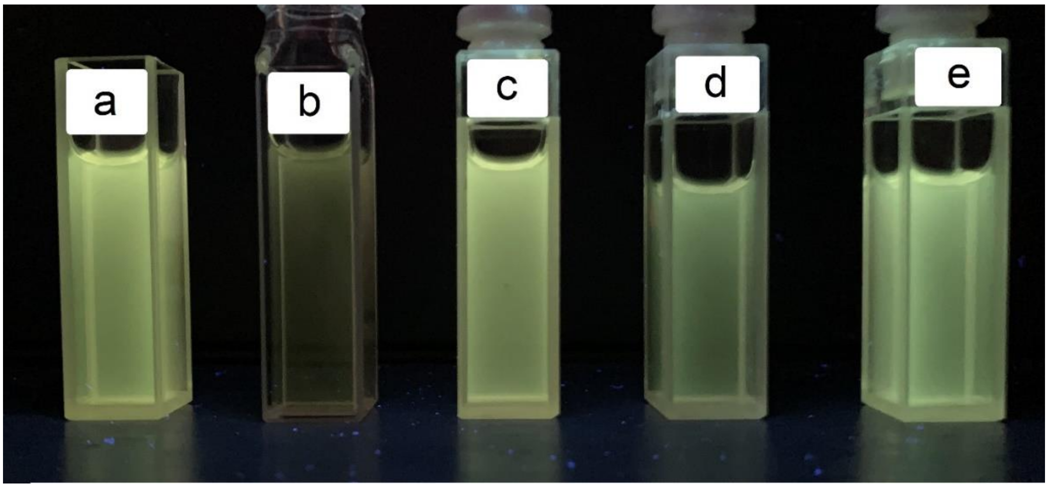

Fig. S70. Changes of fluorescence intensity under UV light, λ = 254 nm : a - Receptor **1** (1.0×10^{-4}M) in dry CH_3CN, b - upon the addition of TBA_2SO_4 (100 equiv.), c - upon the addition of TBACl (100 equiv.), d - upon the addition of TBABr (100 equiv.), e - upon the addition of $TBANO_3$ (100 equiv.).

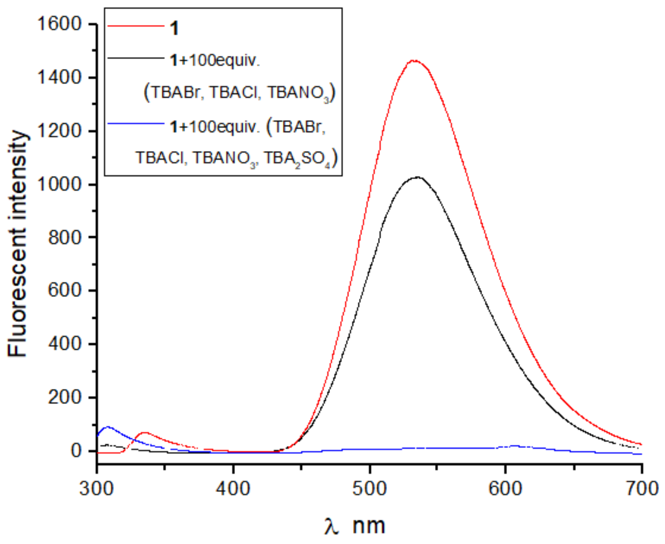

Fig. S71. Emission spectra of Receptor **1** (1.0×10^{-4}M) and upon addition of TBA salts (excitation at 327 nm) in dry CH_3CN.

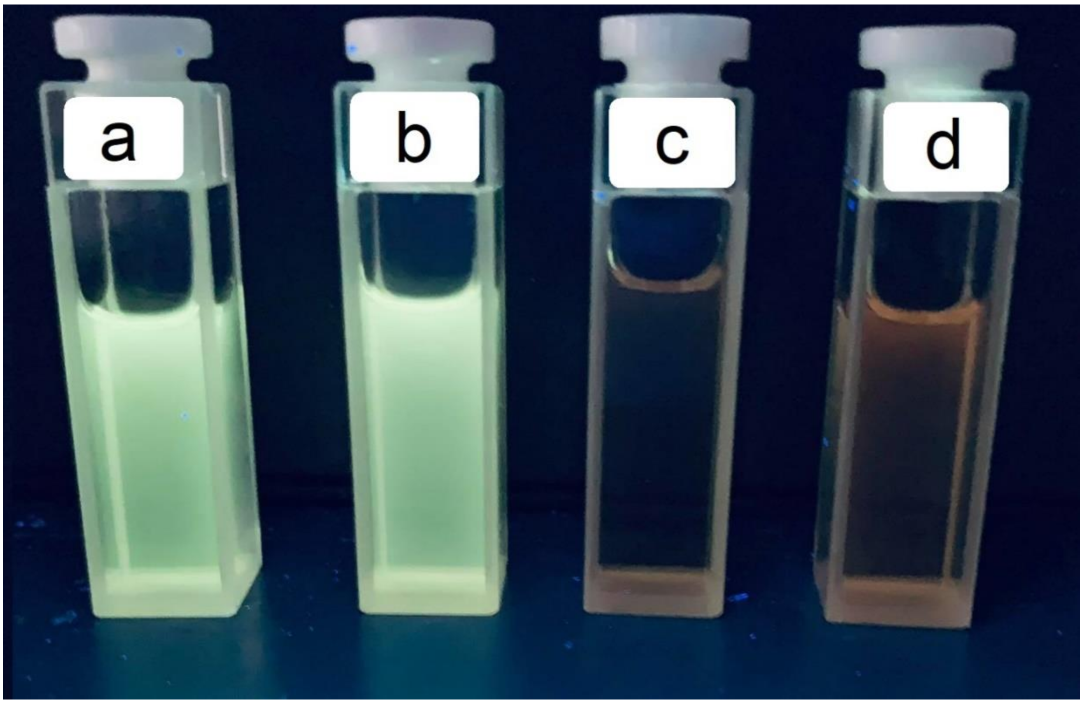

Fig. S72. Changes observed fluorescence intensity under UV light, $\lambda = 254$ nm : a - Receptor **1** (1.0×10^{-4}M) in dry CH_3CN, b - upon the addition of 100 equiv. of TBABr, TBACl, $TBANO_3$ (33.3 equiv. each salt), c - upon the addition of 100 equiv. of TBABr, TBACl, $TBANO_3$, TBA_2SO_4 (25 equiv. each salt), d - upon the addition of 100 equiv. TBA_2SO_4.

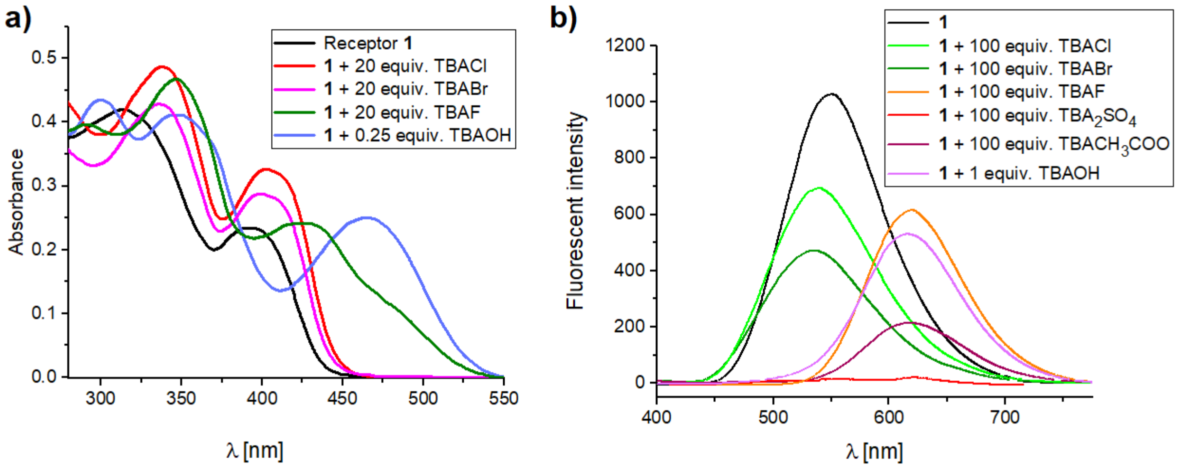

Fig. S73. UV-vis spectra of Receptor **1** (2.0×10^{-5} M) (a) and Emission spectra (b) of Receptor **1** (1.0×10^{-4} M) and upon addition of TBA salts (excitation at 327 nm) in dry CH_3CN.

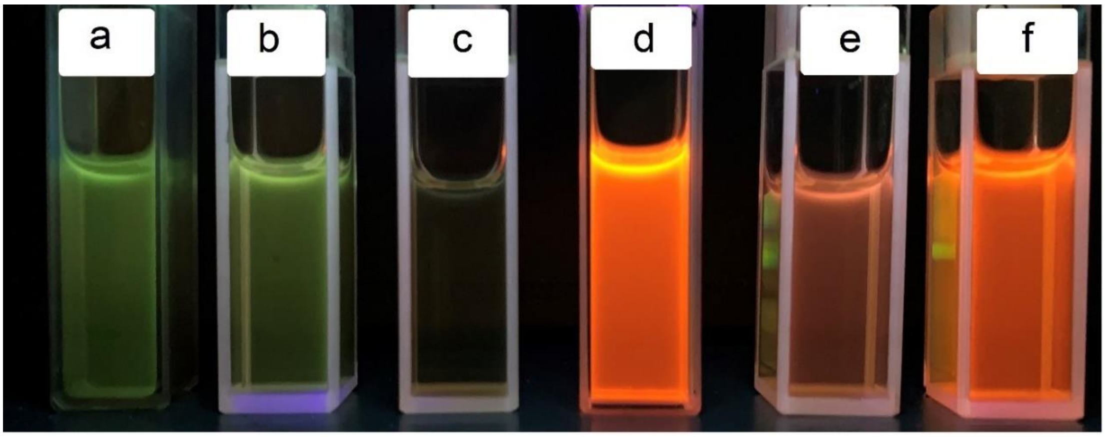

Fig. S74. Changes observed fluorescence intensity under UV light, λ = 254 nm : a - Receptor **1** (1.0×10^{-4}M) in dry CH_3CN, b - upon the addition of 100 equiv. of TBACl, c - upon the addition of 100 equiv. of TBA_2SO_4, d - upon the addition of 100 equiv. TBAF, e - upon the addition of 100 equiv. of $TBACH_3COO$, f - upon the addition of 1 equiv. of TBAOH .

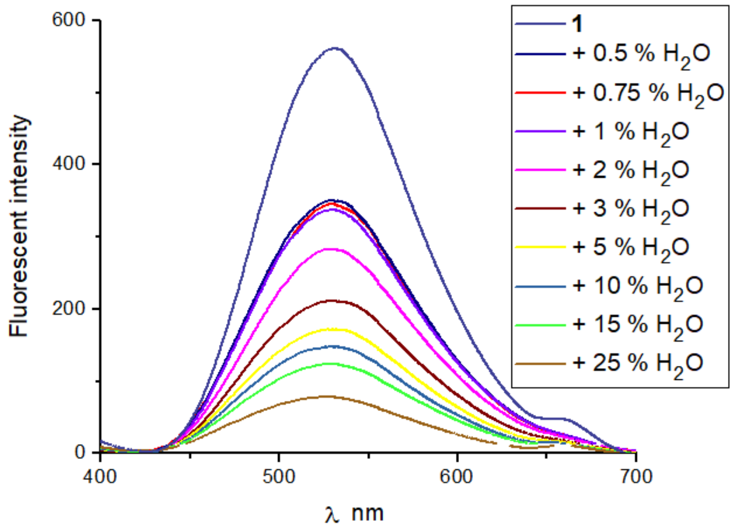

Fig. S75. Emission spectra of Receptor **1** (1.5×10^{-4}M) and upon addition of deionized water (excitation at 327 nm) in CH_3CN.

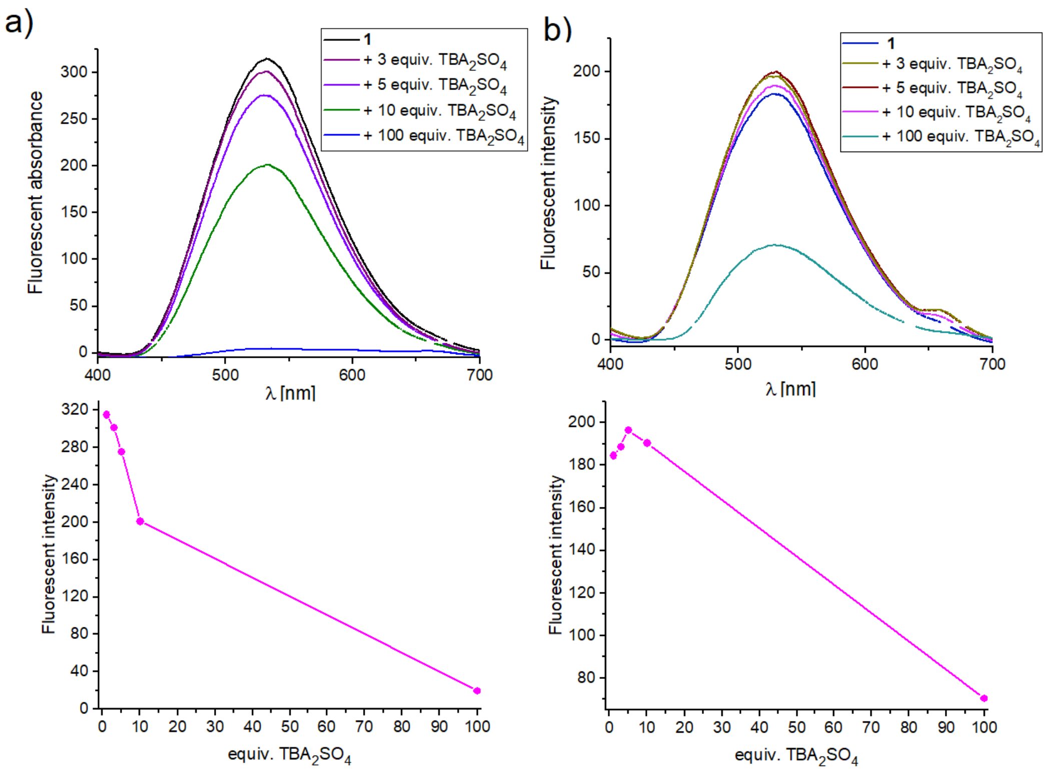

Fig. S76. Comparison of emission spectra of Receptor **1** (2.4×10^{-4}M) upon addition of TBA_2SO_4 (excitation at 327 nm) in dry CH_3CN (a) and in 1% of H_2O in CH_3CN (b).

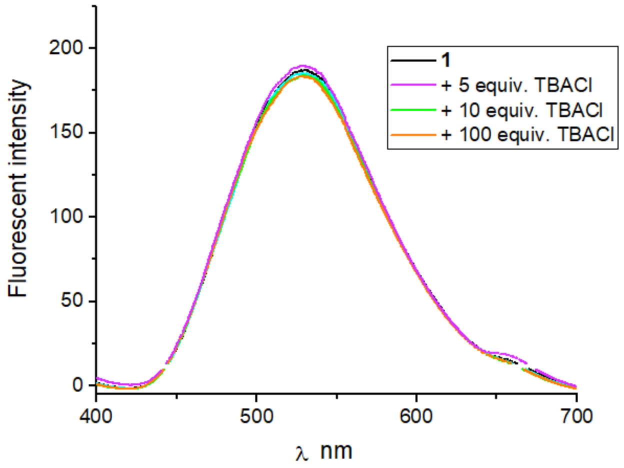

Fig. S77. Emission spectra of Receptor **1** (2.4×10^{-4}M) and upon addition of TBACl (excitation at 327 nm) in 1% of H_2O in CH_3CN.

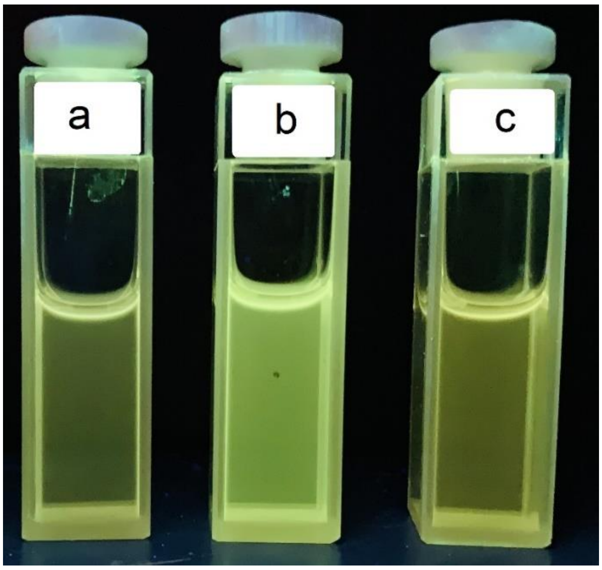

Fig. S78. Changes observed fluorescence intensity under UV light, λ = 254 nm : a - **1** (2.4×10^{-4}M) in CH_3CN upon the addition of 1 % H_2O, b - Receptor **1** (2.4×10^{-4}M) in 1% of H_2O in CH_3CN upon the addition of and 5 equiv. TBA_2SO_4, c - Receptor **1** (2.4×10^{-4}M) in 1% of H_2O in CH_3CN upon the addition of and 5 equiv. TBACl.

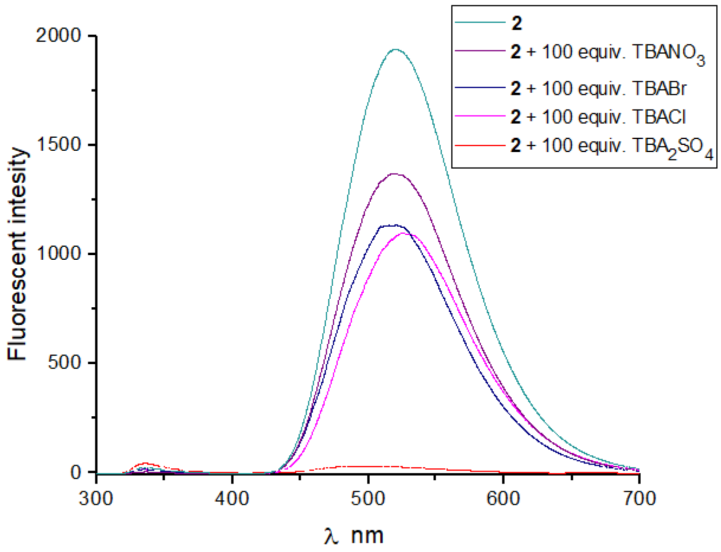

Fig. S79. Emission spectra of Receptor **2** (1.0×10^{-4}M) and upon addition of TBA salts (excitation at 345 nm) in CH_3CN.

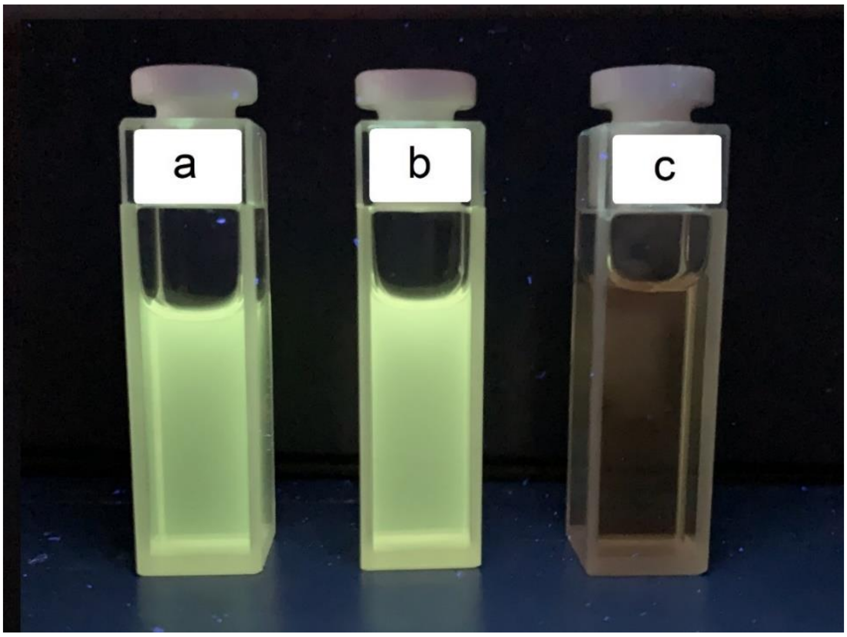

Fig. S80. Changes of fluorescence intensity under UV light, λ = 254 nm: a - Receptor **2** (1.0×10^{-4}M) in dry CH_3CN, b - upon the addition of TBACl (100 equiv.), c - upon the addition of TBA_2SO_4 (100 equiv.).

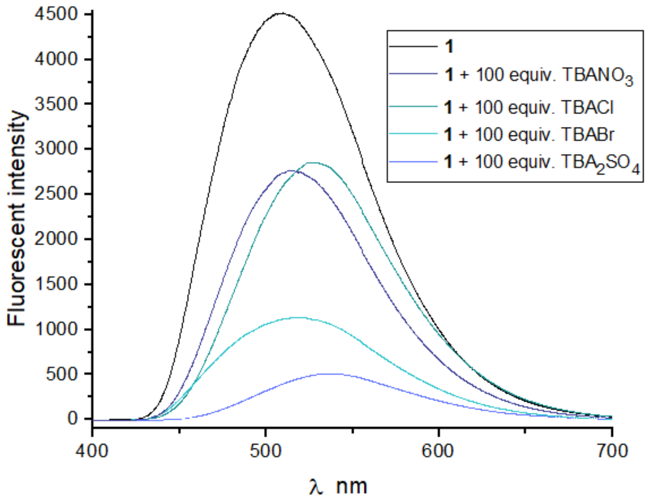

Fig. S81. Emission spectra of Receptor **1** (1.0×10^{-4}M) and upon addition of TBA salts (excitation at 327 nm) in dry $CHCl_3$.

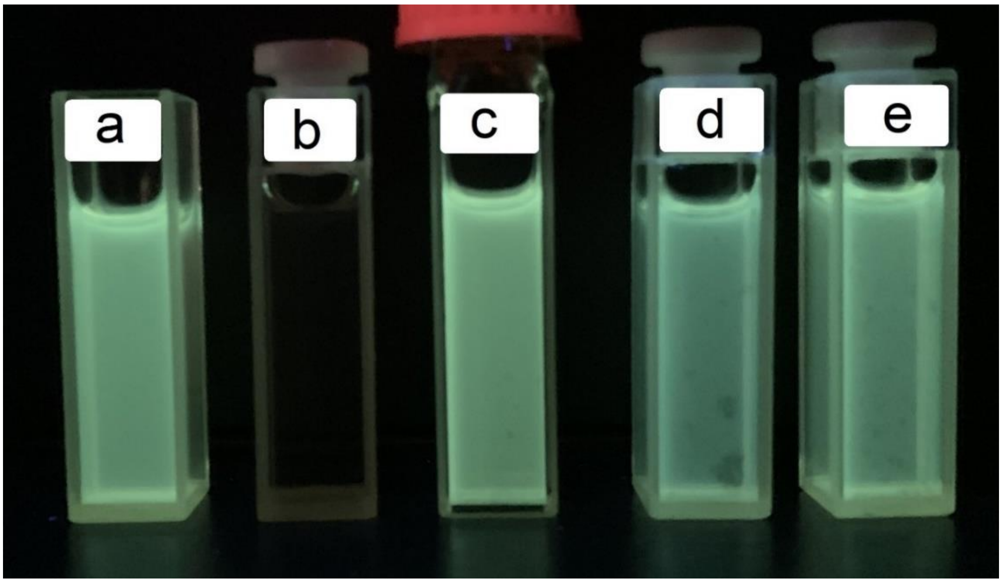

Fig. S82. Changes observed fluorescence intensity under UV light, λ = 254 nm: a - Receptor **1** (1.0 mM) in dry $CHCl_3$, b - upon the addition of TBA_2SO_4 (100 equiv.), c - upon the addition of TBACl (100 equiv.), d - upon the addition of TBABr (100 equiv.), e - upon the addition of $TBANO_3$ (100 equiv.).

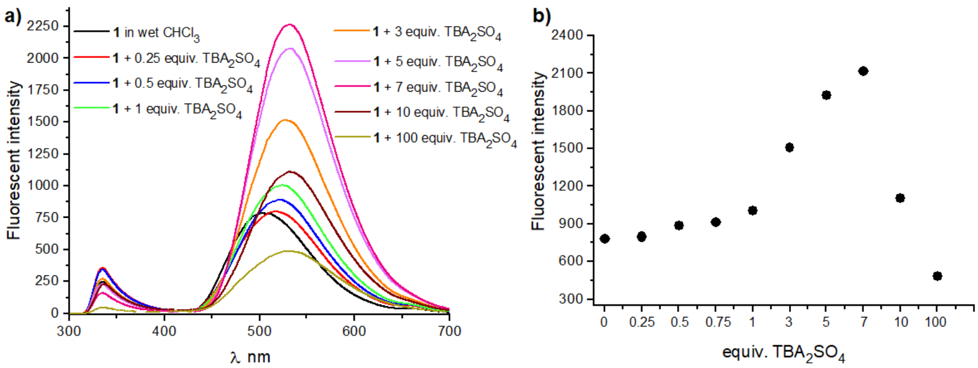

Fig. S83. a) Emission spectra of Receptor **1** (1.0×10^{-4} M) and upon addition of TBA_2SO_4 (excitation at 327 nm) in wet $CHCl_3$. b) Changes in fluorescent intensity for Receptor **1** (1.0×10^{-4} M) and upon addition of TBA_2SO_4.

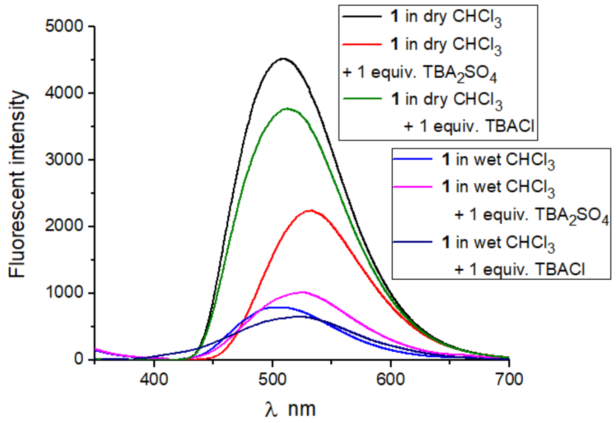

Fig. S84. Emission spectra of Receptor **1** (1.0×10^{-4} M) and upon addition of TBA salts (excitation at 327 nm) in dry and wet $CHCl_3$.

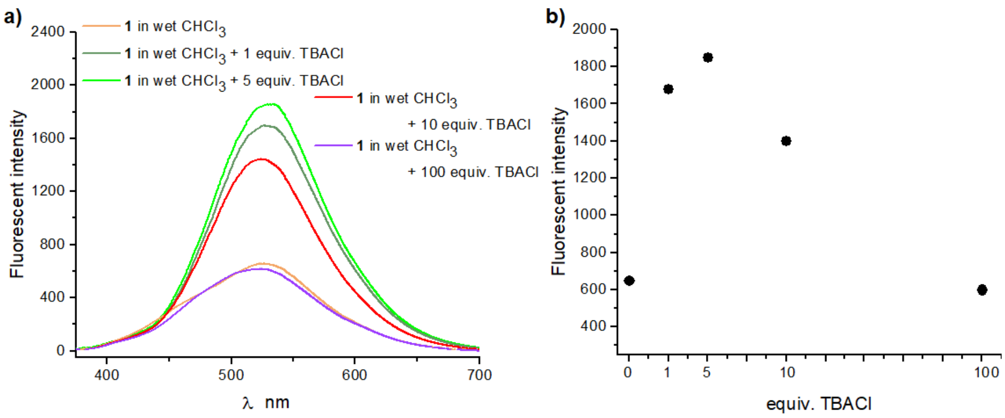

Fig. S85. a) Emission spectra of Receptor **1** (1.0×10^{-4} M) and upon addition of TBACl (excitation at 327 nm) in wet $CHCl_3$. b) Changes in fluorescent intensity for Receptor **1** (1.0×10^{-4} M) and upon addition of TBACl.

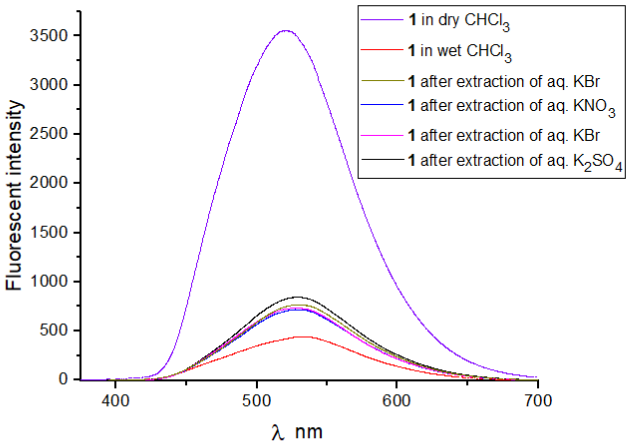

Fig. S86. Emission spectra of Receptor **1** (1.0×10^{-4} M) and after extraction with aqueous potassium salts 5 mM (excitation at 327 nm) in $CHCl_3$.

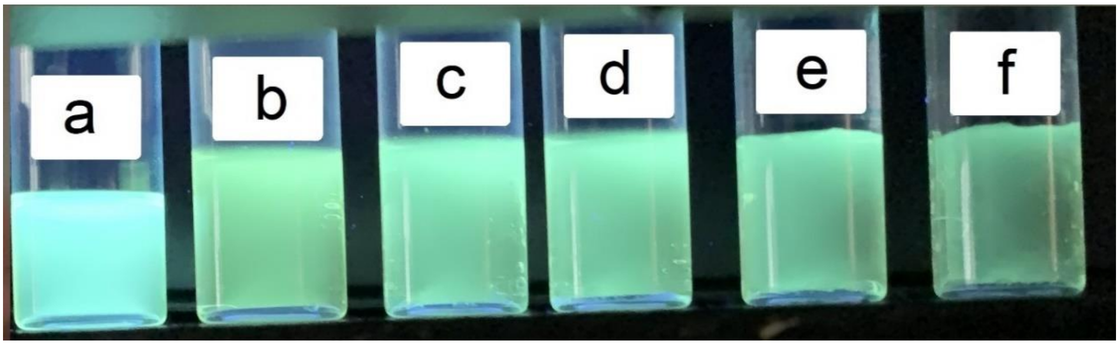

Fig. S87. Changes observed fluorescence intensity under UV light, λ = 254 nm: a - Receptor 1 (1.0 mM) in dry $CHCl_3$, b - Receptor 1 (1.0 mM) in wet $CHCl_3$, c - after extraction of aqueous solution of K_2SO_4 (5 mM), d - after extraction of aqueous solution of KCl (5 mM), e - after extraction of aqueous solution of KBr (5 mM), f - after extraction of aqueous solution of KNO_3 (5 mM).

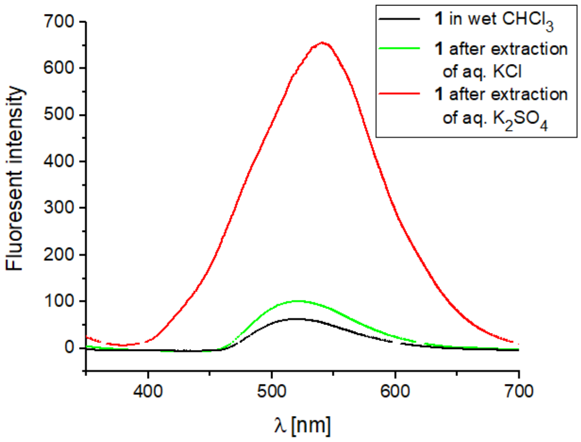

Fig. S88. Emission spectra of Receptor **1** (2.3×10^{-3} M) and after extraction with aqueous potassium salts 50 mM (excitation at 327 nm) in $CHCl_3$.

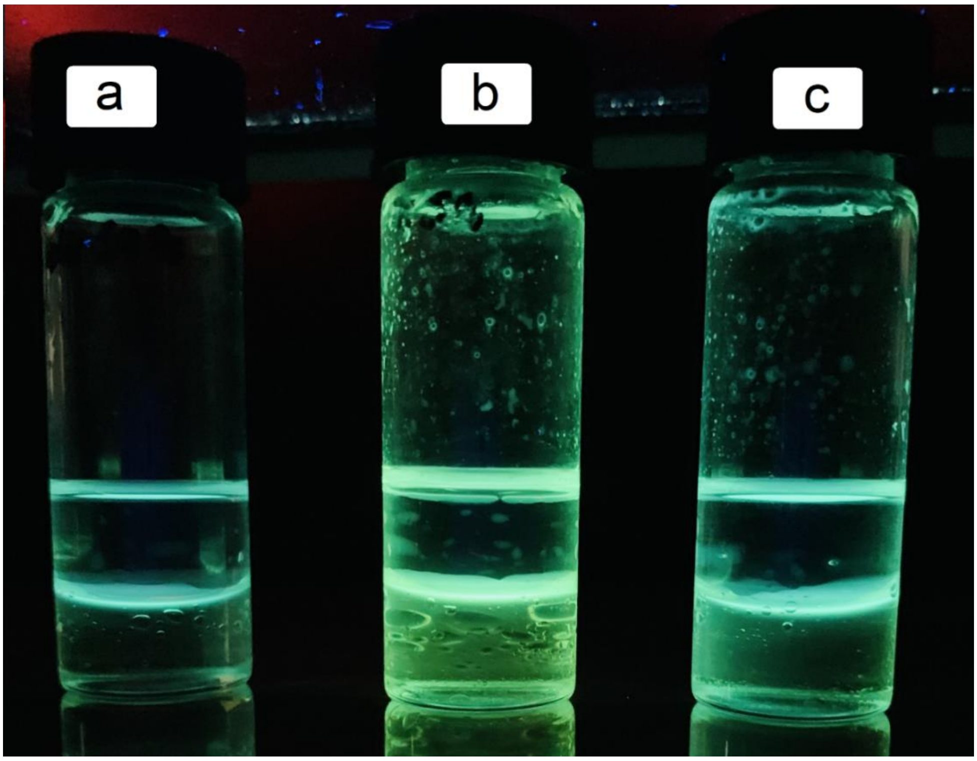

Fig. S89. Changes observed fluorescence intensity under UV light, λ = 254 nm: a - Receptor 1 (2.3 mM) in wet $CHCl_3$, b - after extraction of aqueous solution of K_2SO_4 (50 mM), c - after extraction of a aqueous solution of KCl (50mM).

Crystal data

1 + KCl/NaCl. The X-ray measurement of **1 + KCl/NaCl** was performed at 100.0(5) K on a Bruker D8 Venture PhotonII diffractometer equipped with a TRIUMPH monochromator and a MoKα fine focus sealed tube (λ = 0.71073 Å). A total of 2130 frames were collected with the Bruker APEX3 program [S2]. The frames were integrated with the Bruker SAINT software package [S3] using a narrow-frame algorithm. The integration of the data using a monoclinic unit cell yielded a total of 60775 reflections to a maximum θ angle of 25.50° (0.83 Å resolution), of which 6564 were independent (average redundancy 9.259, completeness = 99.9%, R_{int} = 4.75%, R_{sig} = 2.71%) and 5491 (83.65%) were greater than $2\sigma(F^2)$. The final cell constants of a = 13.0006(19) Å, b = 30.149(4) Å, c = 9.0389(13) Å, β = 93.713(6)°, V = 3535.4(9) Å^3, are based upon the refinement of the XYZ-centroids of 9843 reflections above 20 $\sigma(I)$ with 4.714° < 2θ < 51.22°. Data were corrected for absorption effects using the Multi-Scan method

(SADABS).[S4] The ratio of minimum to the maximum apparent transmission was 0.944. The calculated minimum and maximum transmission coefficients (based on crystal size) are 0.877 and 0.990.

The structure was solved and refined using SHELXTL Software Package[S5, S6] using the space group $P2_1/c$, with $Z = 2$ for the formula unit, $C_{71.75}H_{79.26}Cl_2K_{1.36}N_4Na_{0.64}O_{17.88}S_{1.88}$ corresponding to $2{\times}C_{34}H_{34}N_2O_8$ + 1.36×KCl + 0.64×NaCl + 1.88×DMSO. The final anisotropic full-matrix least-squares refinement on F^2 with 470 variables converged at $R1$ = 3.76%, for the observed data and $wR2$ = 7.84% for all data. The goodness-of-fit was 1.093. The largest peak in the final difference electron density synthesis was 0.317 $e^-/Å^3$ and the largest hole was -0.200 $e^-/Å^3$ with an RMS deviation of 0.044 $e^-/Å^3$. On the basis of the final model, the calculated density was 1.393 g/cm^3 and $F(000)$, 1555 e^-.

The structure in the asymmetric part contains one molecule of the receptor **1**, non-stoichiometric amount of K^+ and Na^+ ions located in close proximity in the crown ether moiety with the refined occupancy of K^+ and Na^+ yielding 0.681(6) and 0.319(6) respectively, and non-stoichiometric amount of DMSO solvent molecule with refined occupancy of 0.938(2). Due to the mixed site structure, the position of Cl^- ions is slightly different depending on the presence of the cation. For both, the cations and anions equal atomic displacement parameters were used. All major component non-H atoms including Na^+ and Cl^- moiety located at two different positions were refined anisotropically. Most hydrogen atoms were placed in calculated positions and were refined within the riding model. Temperature factors of these hydrogen atoms were not refined and were set to be either 1.2 or 1.5 times larger than U_{eq} of the corresponding heavy atom. Positions of two hydrogen atoms engaged in hydrogen bonds (all N-H groups) were freely refined including their isotropic temperature parameters. The atomic scattering factors were taken from the International Tables [6]. Molecular graphics were prepared using the program Mercury 4.1 [S8]. The details concerning crystal data and refinement parameters for 1 + KCl/NaCl are collected in Table S2. Thermal ellipsoids parameters for 1 + KCl/NaCl are presented at 50% probability level in Figure S90 a).

1 + NaBr. The X-ray measurement of 1 + NaBr was performed at 100.0(5) K on a Bruker D8 Venture PhotonII diffractometer equipped with a TRIUMPH monochromator and a MoKα fine focus sealed tube (λ = 0.71073 Å). A total of 2130 frames were collected with the Bruker APEX3 program.[S2] The crystal is composed of two components that were used during data reduction. The frames were integrated with the Bruker SAINT software package[S4] using a narrow-frame algorithm. The integration of the data using a monoclinic unit cell yielded a total of 110006 reflections to a maximum θ angle of 25.71° (0.819 Å resolution) with completeness = 100.0%, R_{int} = 8.10%, R_{sig} = 2.59%. For the structure refinement, a maximum θ angle was cut down to 25.50° giving 5786 independent reflections with 5062 (87.49%) greater than $2\sigma(F^2)$. The final cell constants of a = 12.3418(19) Å, b = 15.454(3) Å, c = 16.593(3) Å, β = 100.756(6)°, V = 3109.2(9) $Å^3$, are based upon the refinement of the XYZ-centroids of 9830 reflections above 20 $\sigma(I)$ with 4.620° < 2θ < 51.18°. Data were corrected for absorption effects using the Multi-Scan

method (TWINABS).[S9] The ratio of minimum to the maximum apparent transmission was 0.733. The calculated minimum and maximum transmission coefficients (based on crystal size) are 0.687 and 0.921. The measured crystal is composed of two slightly rotated domains with partial overlap of the reflections. Data integration was based on these two domains resulting in final data merged in HKLF4 format. The calculated domains fraction ratio yields 0.666:0.334. The structure was solved and refined using SHELXTL Software Package[S5, S6] using the space group $P2_1/c$, with $Z = 4$ for the formula unit, $C_{34}H_{34}BrN_2NaO_8$ corresponding to $C_{34}H_{34}N_2O_8$ + NaBr. The final anisotropic full-matrix least-squares refinement on F^2 with 481 variables converged at $R1 = 4.52\%$, for the observed data and $wR2 = 11.49\%$ for all data. The goodness-of-fit was 1.076. The largest peak in the final difference electron density synthesis was 0.652 $e^-/Å^3$ and the largest hole was -0.469 $e^-/Å^3$ with an RMS deviation of 0.068 $e^-/Å^3$. On the basis of the final model, the calculated density was 1.499 g/cm^3 and $F(000)$, 1448 e^-.

The structure contains in the asymmetric part a single molecule of the receptor **1** and NaBr moiety as ionic pair. The structure is disordered with two alternative positions of Na^+ and Br^- ions including also alternative positions of an aliphatic chain of the crown ether moiety in the receptor. The refined occupancy ratio of all disordered over two positions atoms yields 0.748(8):0.252(8). To preserve reasonable geometry of the lower occupancy aliphatic chain the similarity restraints were used. Due to the close proximity of the disordered fragments, a number of restraints for the atomic displacement parameters were also used. All major component non-H atoms including Br^- moiety located at two different positions were refined anisotropically. Most hydrogen atoms were placed in calculated positions and were refined within the riding model. Temperature factors of these hydrogen atoms were not refined and were set to be either 1.2 or 1.5 times larger than U_{eq} of the corresponding heavy atom. Positions of two hydrogen atoms engaged in hydrogen bonds (all N-H groups) were freely refined including their isotropic temperature parameters. The atomic scattering factors were taken from the International Tables [S7]. Molecular graphics were prepared using the program Mercury 4.1 [S8]. The details concerning crystal data and refinement parameters for 1 + NaBr are collected in Table S2. Thermal ellipsoids parameters for 1 + NaBr are presented at 50% probability level in Figure S90 b).

Table S2. Crystal data and refinement parameters for 1 + KCl/NaCl and 1 + NaBr.

**Identification code**	**1 + KCl/NaCl**	**1 + NaBr***
**Chemical formula**	$C_{71.75}H_{79.26}Cl_2K_{1.36}N_4Na_{0.64}O_{17.88}S_{1.88}$ corresponding to: 2×$C_{34}H_{34}N_2O_8$ + 1.36×KCl + 0.64×NaCl + 1.88×DMSO	$C_{34}H_{34}BrN_2NaO_8$ corresponding to: $C_{34}H_{34}N_2O_8$ + NaBr
M_x/ **g·mol**$^{-1}$	1482.55	701.53
***T*/ K**	100.0(5)	100.0(5)
***λ*/ Å**	0.71073	0.71073

**Crystal size**	0.032×0.080×0.441	0.060×0.270×0.29
**Space group**	$P2_1/c$	$P2_1/c$
**Unit cell dimensions**	a = 13.0006(19) Å<br>b = 30.149(4) Å<br>c = 9.0389(13) Å<br>β = 93.713(6)°	a = 12.3418(19) Å<br>b = 15.454(3) Å<br>c = 16.593(3) Å<br>β = 100.756(6)°
**V/ Å^3, Z**	3535.4(9), 2	3109.2(9), 4
**D_x/ g·cm^{-3}**	1.393	1.499
**μ/ mm^{-1}**	0.305	1.393
**F(000)**	1555	1448
**θ_{min}, θ_{max}**	2.36°, 25.50°	2.13°, 25.50°
**Index ranges**	$-15 \leq h \leq 15$<br>$-36 \leq k \leq 36$<br>$-10 \leq l \leq 10$	$-14 \leq h \leq 14$<br>$0 \leq k \leq 18$<br>$0 \leq l \leq 20$
**Reflections collected/ Independent**	60775/ 6564<br>R_{int}=0.0475	110006**/ 5786<br>R_{int}=0.0810**
**Data completeness**	99.9%	100.0%
**Absorption correction**	Multi-Scan	Multi-Scan
**T_{max}, T_{min}**	0.990, 0.877	0.921, 0.687
**Refinement method**	Full-matrix LSQ on F^2	Full-matrix LSQ on F^2
**Data / restraints / parameters**	6564 / 0 / 470	5786 / 76 / 481
**Gof on F^2**	1.093	1.076
**Final R indices**	5491 data; I>2σ(I)<br>$R1$ = 0.0376, $wR2$ = 0.0738<br>all data<br>R1 = 0.0505, $wR2$ = 0.0784	5062 data; I>2σ(I)<br>$R1$ = 0.0452, $wR2$ = 0.1107<br>all data<br>$R1$ = 0.0548, $wR2$ = 0.1149
**Extinction coefficient**	0.0014(2)	-
**$\Delta\rho_{max}$, $\Delta\rho_{min}$**	0.317 e·Å^{-3}, -0.200 e·Å^{-3}	0.652 e·Å^{-3}, -0.469 e·Å^{-3}

* Data reduction performed for two domains of multi-component crystal. Structure refinement based on merged reflections.

** Data corresponding to 2θ range up to 51.42°.

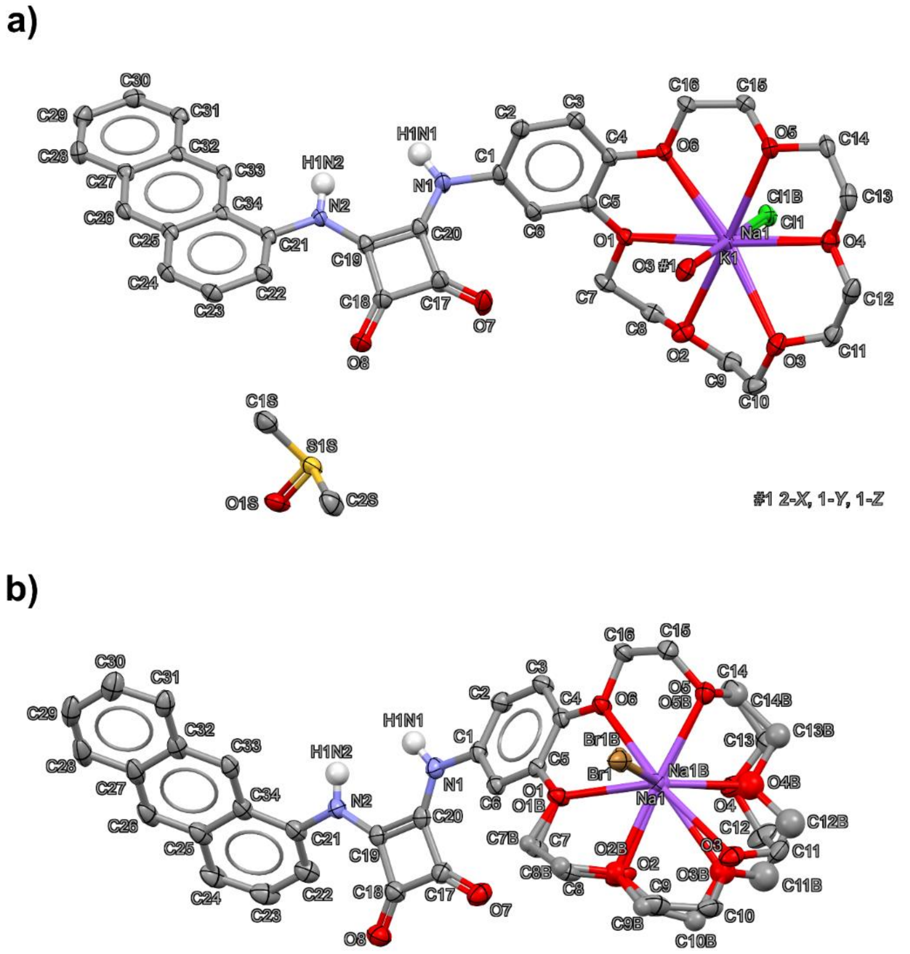

Figure S90. Thermal ellipsoids of atoms at 50% probability level for the structures of 1 + KCl/NaCl **a)** and 1 + NaBr **b)** together with a numbering scheme. All C-H hydrogen atoms were omitted for clarity. The data analysis for the obtained crystals was carried out with help of procedures described in references [S2-S9]. CCDC 2085348 and 2085349 contain the supplementary crystallographic data for this paper. These data can be obtained free of charge from The Cambridge Crystallographic Data Centre via www.ccdc.cam.ac.uk/structures.

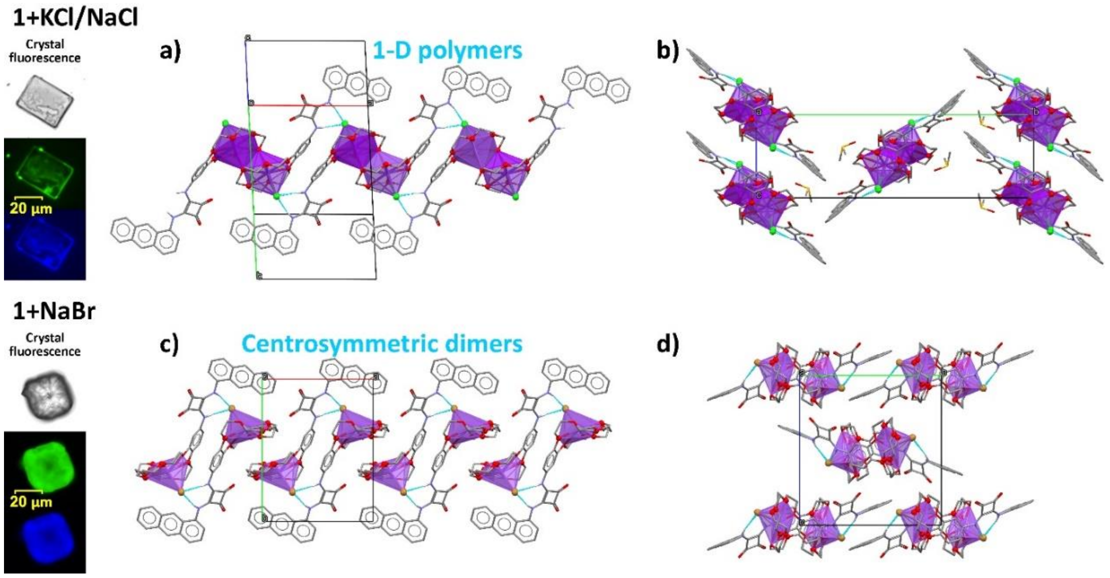

Fig. S91. Image presenting fluorescence of the crystals of **1** with KCl/NaCl and **1** with NaBr salts together with the molecular arrangements of moieties in the solid state: 1-D polymers in **1+KCl/NaCl a)**, **b)** and centrosymmetric dimmers in **1+NaBr c), d)**. All C-H hydrogen atoms were omitted for clarity.

References

[S1] Zaleskaya M, Jagleniec D, Karbarz M, Dobrzycki Ł, Romański J, Squaramide based ion pair receptors possessing ferrocene as a signaling unit, *Inorg. Chem. Front*. **2020**;7:972–983.

[S2] APEX3, *Bruker Nano Inc.* **2019.**

[S3] SAINT, *Bruker Nano Inc.* **2019**.

[S4] SADABS, *Bruker Nano Inc.* **2019**. TWINABS

[S5] Sheldrick GM. SHELXT-Integrated Space-Group and Crystal-Structure Determination, *Acta Crystallogr.* **2015;**A71:3-8

[S6] Sheldrick GM. Crystal Structure Refinement with SHELXL, *Acta Crystallogr*. **2015**; C71:3–8.

[S7] Macrae CF, Bruno IJ, Chisholm JA, Edgington PR, McCabe P, Pidcock E, Rodriguez-Monge L, Taylor R, Streek J, Wood P. A. Mercury CSD 2.0 – New Features for the Visualization and Investigation of Crystal Structures, *J. Appl. Cryst*. **2008**;41:466-470.

[S8] *International Tables for Crystallography*, Ed. Wilson, A. J. C. Kluwer: Dordrecht, **1992**, Vol.C.

[S9] TWINABS, *Bruker Nano Inc.*, **2019.**
